# Dehaloperoxidase Catalyzed Stereoselective Synthesis of Cyclopropanol Esters

**DOI:** 10.1021/acs.joc.2c02030

**Published:** 2022-12-21

**Authors:** Mary G. Siriboe, David A. Vargas, Rudi Fasan

**Affiliations:** † Department of Chemistry, University of Rochester, 120 Trustee Road, Rochester, New York14627, United States

## Abstract

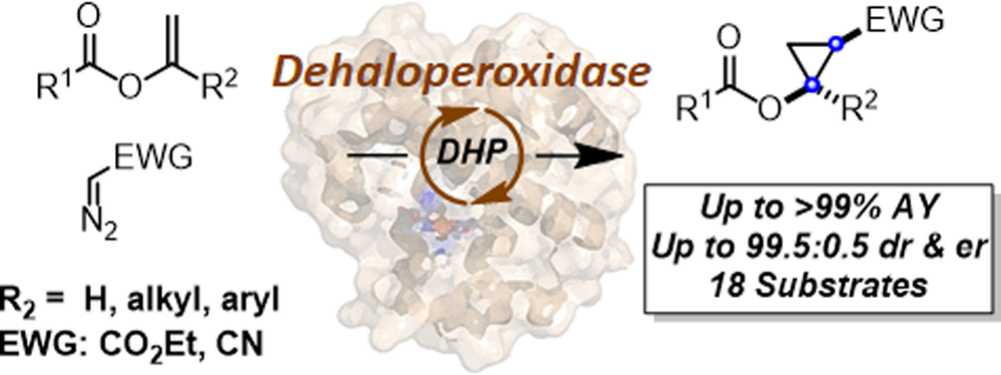

Chiral cyclopropanols are highly desirable building blocks for medicinal chemistry, but the stereoselective synthesis of these molecules remains challenging. Here, a novel strategy is reported for the diastereo- and enantioselective synthesis of cyclopropanol derivatives via the biocatalytic asymmetric cyclopropanation of vinyl esters with ethyl diazoacetate (EDA). A dehaloperoxidase enzyme from *Amphitrite ornata* was repurposed to catalyze this challenging cyclopropanation reaction, and its activity and stereoselectivity were optimized via protein engineering. Using this system, a broad range of electron-deficient vinyl esters were efficiently converted to the desired cyclopropanation products with up to 99.5:0.5 diastereomeric and enantiomeric ratios. In addition, the engineered dehaloperoxidase-based biocatalyst is able to catalyze a variety of other abiological carbene transfer reactions, including N–H/S–H carbene insertion with EDA as well as cyclopropanation with diazoacetonitrile, thus adding to the multifunctionality of this enzyme and defining it as a valuable new scaffold for the development of novel carbene transferases.

## Introduction

Asymmetric olefin cyclopropanation reactions are transformations of high relevance in organic and medicinal chemistry due to the abundance of chiral cyclopropane rings in bioactive molecules and drugs.^1^ While transition metal catalysts have been traditionally applied for asymmetric cyclopropanations,^1^ our group and others have recently shown that heme-containing proteins and enzymes, such as myoglobin^2^ and cytochromes P45s,^3^ as well as artificial enzymes,^4^ can serve as efficient biocatalysts for abiological olefin cyclopropanation reactions. These biocatalytic strategies were shown to offer key advantages over chemocatalytic methods in terms of chemo- and stereoselectivity, catalytic efficiency, and/or step economy for drug synthesis.^2b^

**Figure 1:**
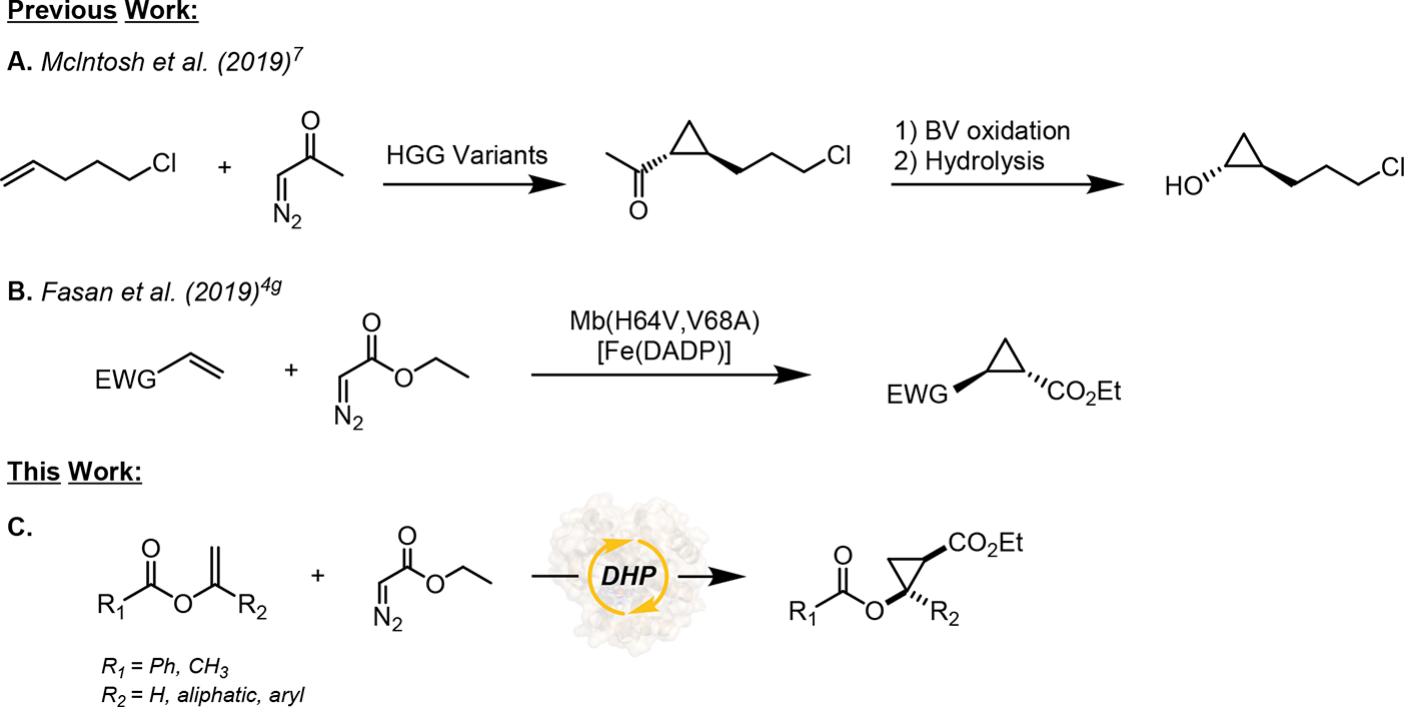
Selected biocatalytic and chemoenzymatic cyclopropanation strategies.

Among the various types of functionalized cyclopropane scaffolds, cyclopropanols are attractive building blocks in medicinal chemistry, constituting the core motif of drug molecules such as grazoprevir, an antiviral agent used for the treatment of chronic hepatitis C virus (HCV) infections.^5^ Although various cyclopropanation methodologies have been developed to prepare substituted/functionalized cyclopropanes,^1a−1d^ methods for the asymmetric synthesis of cyclopropanols have been largely missing.^6^ Recently, a chemoenzymatic approach for the generation of the cyclopropanol core of grazoprevir was reported by McIntosh et al. (Figure 1a).^7^ In this work, a stereoselective cyclopropanation of 5-chloropentene with diazoacetone (70% ee) was first carried out using an engineered variant of Hells’ Gate globin I, a hemoprotein structurally homologous to myoglobin.^2a^ The resulting cyclopropyl ketone was then subjected to Baeyer–Villiger oxidation and hydrolysis to produce the desired chiral cyclopropanol scaffold.^7^ Despite this progress, this approach involves multiple steps (3) and it was demonstrated only on a single target substrate. Prior to this work, the same scaffold was afforded through a longer synthetic sequence involving the construction of the cyclopropyl ketone intermediate via a [1,3]-phosphorus-Brook rearrangement of a phosphonate precursor.^6^

To expand opportunities for the biocatalytic synthesis of chiral cyclopropanols, we envisioned these compounds could be more readily accessed through the cyclopropanation of vinyl esters, this strategy providing a more direct means to produce cyclopropanol scaffolds in the form of protected esters (Figure 1c). One important challenge presented by this approach, however, is the poor reactivity of the electron deficient olefinic group of vinyl esters toward cyclopropanation, in particular by action of carbene transfer catalysts that operate via electrophilic metallocarbenoid species, including rhodium-based catalysts (e.g., Rh_2_(OAc)_4_) or iron-porphyrins (e.g., Fe(TPP)Cl).^4g^

**Figure 2:**
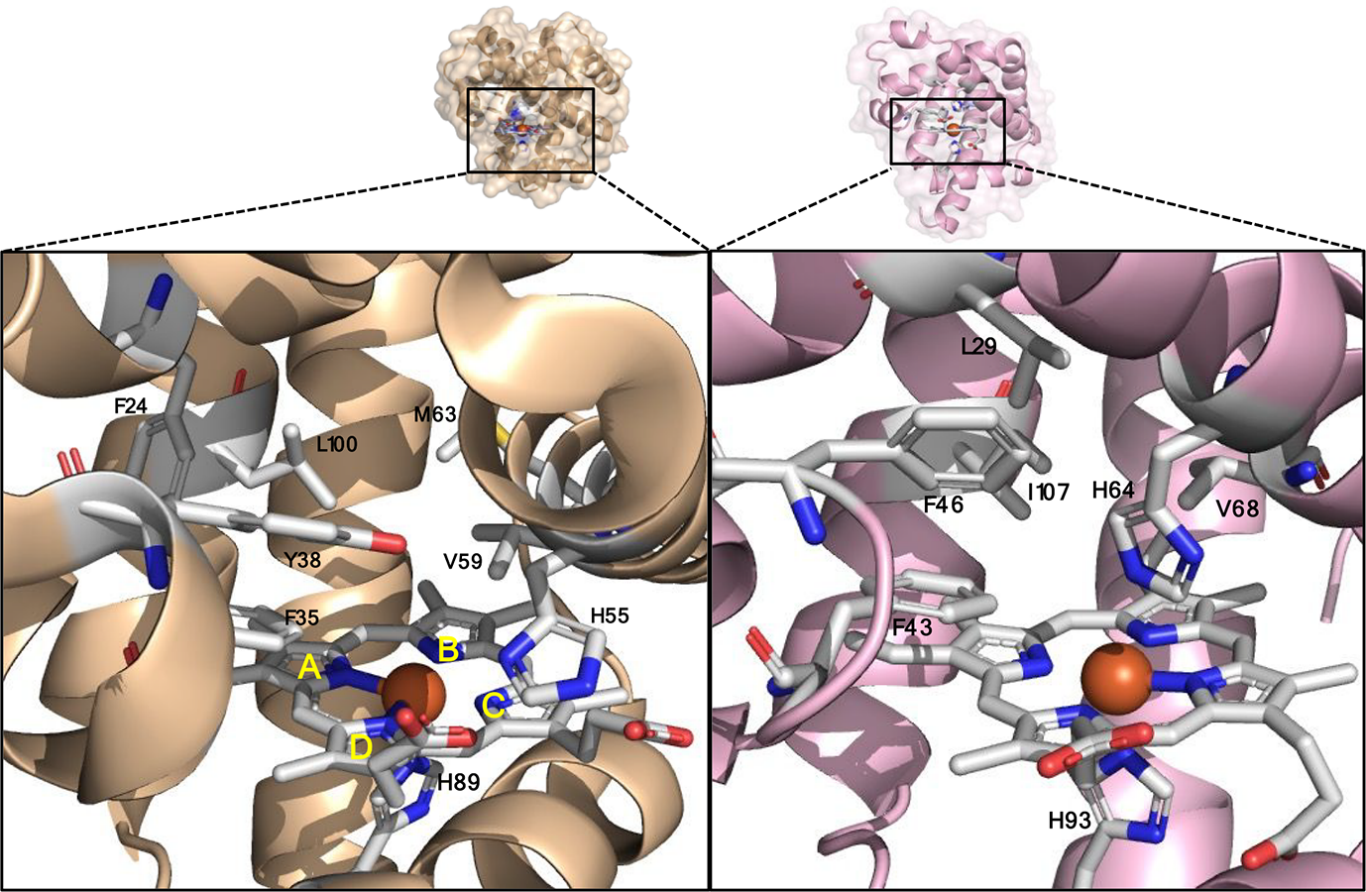
Active site of dehaloperoxidase (DHP) (left, PDB 1EW6) and active site of myoglobin (Mb) (right, PDB 1A6K). The heme cofactor and residues in close proximity to the Fe center are shown as sticks in white. Heme pyrrole rings are labeled A–D according to the standard convention.

In previous work, we found that Mb-based artificial metalloenzymes containing a non-native cofactor/axial ligand and increased (more positive) redox potential (i.e., *E*°_Fe^3+^/Fe^2+^_ = 146 mV vs 47 mV for Mb) exhibit a peculiar reactivity toward the cyclopropanation of electron deficient olefins (Figure 1b).^4g^ Building upon this finding, we hypothesized that naturally occurring hemoproteins possessing a relative high redox potential (i.e., *E*°_Fe^3+^/Fe^2+^_ > 150–200 mV) could provide a viable catalyst for the cyclopropanation of electrodeficient vinyl esters. Accordingly, we selected *Amphitrite ornata* dehaloperoxidase (DHP), which is a small enzyme (15.5 kDa) containing a histidine-ligated heme cofactor (Figure 2).^8^ As part of its native function, this enzyme exhibits multifunctional activity by catalyzing the hydrogen peroxide (H_2_O_2_)-dependent dehalogenation of halophenol pollutants into quinone products as well as the oxidation of other aromatic compounds via peroxidase and oxidase activity.^8a,9^ Importantly, the enzyme displays a relatively high redox potential (221 mV), which is 100–150 mV higher than Mb (54 mV) and the average values of other globins.^10^ Herein, we report the engineering and application of DHP-based engineered variants as efficient and stereoselective biocatalysts for the cyclopropanation of vinyl esters with diazoacetate and diazoacetonitrile as the carbene donors (Figure 1c). This work provides a biocatalytic strategy for the stereoselective synthesis of cyclopropanol motifs and it provides a first, proof-of-principle demonstration of the use of a dehaloperoxidase enzyme as biocatalyst for abiotic carbene transfer reactions.

## Results and Discussion

### Design and Screening of DHP-Catalyzed Cyclopropanation

In order to test the functionality of DHP as a carbene transferase, we began by testing its activity in a cyclopropanation reaction with styrene (**1a**) and ethyl α-diazoacetate (EDA, **2**) in the presence of a reductant (sodium dithionite) under anaerobic conditions. Promisingly, DHP exhibited activity in this cyclopropanation reaction (35% assay yield (GC)), producing ethyl *trans*-2-phenylcyclopropane-1-carboxylate **(±)-3a** as the major product with good diastereoselectivity (91:9 diastereomeric ratio (dr)) but no enantioselectivity (Entry 2, Table 1). This level of activity is comparable to that of wild type Mb, which catalyzes this reaction in 36% yield, similar *trans*-selectivity (93:7 dr) and very low enantioselectivity (53:47 enantiomeric ratio (er)) (Entry 1, Table 1).^2a^ Given our prior success in improving the carbene transferase activity of Mb via active site mutagenesis,^2a^ we investigated the possibility to improve the catalytic activity and stereoselectivity of DHP via mutagenesis of amino acid residues located in close proximity to the heme cofactor, as identified based on the available crystal structure of this enzyme (PDB: 1EW6, Figure 2).^8b^ Specifically, residues His55, Val55, Met63, Try38, Leu100, Phe24, and Phe35 were mutated to alanine in order to generate an “alanine-scanning” library for initial structure–activity relationship analyses.

**Table 1:**
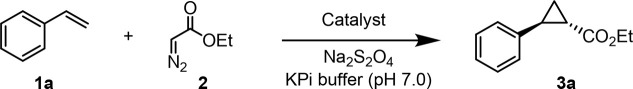
Activity and Selectivity of Dehaloperoxidase Active Site Variants toward Styrene Cyclopropanation with Ethyl Diazoacetate^a^

entry	catalyst	AY (%)^c^	dr_trans_^d^	er_(1*S*,2*S*)_	TON
1^b^	Mb WT	36	93:7	53:47	180
2	DHP WT	35	91:9	50:50	175
3	DHP(H55A)	48	91:9	52:48	239
4	DHP(V59A)	4	93:7	84:16	18
5	DHP(Y38A)	42	92:8	49:51	209
6	DHP(L100A)	37	91:9	49:51	187
7	DHP(M63A)	2	79:21	70:30	8
8	DHP(F35A)	51	92:8	48:52	256
9	DHP(F24A)	51	92:8	49:51	254
10	DHP(H55A, V59A)	>99	99.5:0.5	99.5:0.5	500

a Reaction conditions: 20 μM protein, 10 mM styrene, 20 mM EDA, 10 mM dithionite, KPi buffer (50 mM, pH 7), 5% EtOH, anaerobic conditions, 4 h.

b As reported in ref (2a).

c Assay yields (AY) were determined by GC analysis using calibration curves generated with isolated products (*n* ≥ 2; SE < 10%).

d Diastereoselectivity and enantioselectivity were determined via chiral SFC and GC chromatography by comparison with authentic standards.

Screening of this DHP variant library showed an improvement in cyclopropanation activity upon alanine substitution of residues His55, Tyr38, Phe24, and Phe35 compared to the parent DHP (from 35 to 42–51% yield; Table 1). For most of these mutations, however, the activity improvement was accompanied by no improvement in the diastereo- or enantioselectivity. As an exception, the V59A and M63A mutations produced a noticeable increase in enantioselectivity, corresponding to 84:16 dr and 70.5:29.5 er for the formation of the (1*S*,2*S*) stereoisomer **3a**, respectively, compared to 0% enantioselectivity for the parent enzyme, albeit this improvement was accompanied by a loss in activity (2–4% yield; Table 1, Entries 4 and 7). Altogether, these results illustrated the plasticity of DHP to be tuned for carbene transferase activity. Additionally, the SAR data gathered from this initial set of variants showed that mutation of the residues in proximity to pyrrole ring B of the heme cofactor (i.e., Val59 and Met63) can influence the stereoselectivity of the cyclopropanation reaction, whereas mutation of residues above rings A, C, and D (i.e., His55, Try38, Phe24, and Phe35) tend to influence catalytic activity.

Based on these results, we sought to combine the activity-enhancing effect of the H55A mutation with the improved enantioselectivity induced by the V59A mutation. Gratifyingly, the corresponding double variant DHP(H55A, V59A) was found to catalyze the cyclopropanation of styrene to **3a** in quantitative yield and with excellent levels of diastereo- and enantiocontrol (99.5:0.5 dr and er) (Entry 10, Table 1). Based on the results for the single site variants, it is clear that the two beneficial mutations had a synergistic effect on both reactivity and stereoselectivity. Interestingly, a similarly high level of *trans*-(1*S*,2*S*) stereoselectivity in this reaction was previously obtained by our group using the engineered Mb variant Mb(H64V, V68A),^2a^ which shared a similar set of large-to-small mutations at the level of the distal histidine (His64 in Mb; His55 in DHP) and same alanine mutation at Val68 (corresponding to Val59 in DHP; Figure 1). The high stereocontrol exhibited by Mb(H64V, V68A) in this reaction was linked to the effect of the V68A mutation in creating a cavity above ring B of the porphyrin ring that better accommodates the ester group of the heme-bound carbene, facilitating high pro-(1*S*,2*S*) attack by the styrene substrate.^11^ Thus, despite the difference in the amino acid composition of the heme pocket in DHP vs Mb (Figure 2), the active site mutations in DHP(H55A, V59A) seem to recapitulate the effect of those in Mb(H64V, V68A), likely following a similar mechanism for exerting stereocontrol in this reaction.

After establishing the cyclopropanation reactivity of DHP and its variants, we tested their performance in the cyclopropanation of the more challenging substrate vinyl benzoate (**4a**) in the presence of EDA (Table 2). Unlike hemin, DHP showed detectable catalytic activity (2% AY) in this reaction producing the desired cyclopropyl benzoate **5a** with low diastereoselectivity (60:40 dr) but promising enantioselectivity (77:23 er) (Entry 2, Table 2). Most of the single-site alanine-scanning variants show comparably low activity (<3% AY) and modest diastereoselectivity (from 58:42 to 72:28 dr). In stark contrast, and mirroring the results with the styrene reaction, the DHP(H55A) variant showed enhanced reactivity (2 → 15% AY), whereas DHP(V59A) showed significantly improved diastereoselectivity and enantioselectivity (69:31 → 96:4 dr and 80:20 → 99.5:0.5 er) (Entry 4, Table 2). Reflecting the combined beneficial effect of these mutations, DHP(H55A, V59A) showed further improved activity as well as excellent diastereo- and enantioselectivity (99.5:0.5 dr and er) (Table 2, Entry 10). This DHP variant was further engineered by replacing His55 with either Val or Gly, as inspired by the beneficial effect of similar mutations at the level of the distal histidine in Mb toward enhancing its cyclopropanation activity.^2e,12^ The corresponding variants DHP(H55V, V59A) and DHP(H55G, V59A), however, were found to show markedly reduced activity compared to DHP(H55A, V59A) (Table S1), thus defining the latter as a superior biocatalyst for this reaction.

**Table 2:**
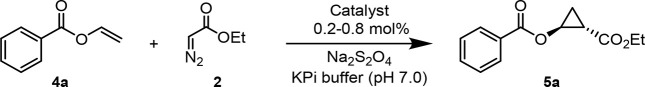
Activity and Selectivity of Dehaloperoxidase toward Cyclopropanation of Vinyl Benzoate with Ethyl Diazoacetate (EDA)^a^

entry	catalyst	AY (%)	dr_trans_	er_trans_	TON
1	Hemin^b^	–	–	–	–
2	DHP WT	2	60:40	77:23	10
3	DHP(H55A)	15	69:31	80:20	76
4	DHP(V59A)	3	96:4	99.5:0.5	13
5	DHP(Y38A)	1	61:9	n.d.	73
6	DHP(L100A)	2	59:41	36	86
7	DHP(M63A)	<1	72:28	n.d.	78
8	DHP(F35A)	1	62:38	n.d.	50
9	DHP(F24A)	1	60:40	n.d.	49
10	DHP(H55A, V59A)	35	99.5:0.5	99.5:0.5	175
11	DHP(H55A, V59A)^c^	80	99.5:0.5	99.5:0.5	100
12	DHP(H55A, V59A)^d^	>99	99.5:0.5	99.5:0.5	125
13	DHP(H55A, V59A)^e^	>99	99.5:0.5	99.5:0.5	1000
14	DHP(H55A, V59A)^f^	21	99.5:0.5	99.5:0.5	–

a Reaction conditions: 20 μM protein, 10 mM vinyl benzoate, 20 mM EDA, 10 mM dithionite, 5% EtOH, anaerobic, 4 h.

b 0.2 mol % hemin dissolved in DMSO.

c 2.5 mM vinyl benzoate, 5 mM EDA, 10% MeOH.

d 2.5 mM vinyl benzoate, 10 mM EDA, 10% MeOH.

e 5 μM protein, 5 mM vinyl benzoate, 20 mM EDA, 10% MeOH.

f Whole cells OD_600_ = 60, 2.5 mM vinyl benzoate, 10 mM EDA.

Next, we sought to optimize the performance of the DHP(H55A, V59A)-catalyzed reaction through optimization of the reaction conditions. Using 10% (v/v) methanol as cosolvent in place of 5% (v/v) ethanol led to a noticeable improvement in the yield of the cyclopropanation reaction (35 → 80%) without affecting stereoselectivity (99.5:0.5 dr and er; Table 2 and Table S2). Furthermore, using these conditions in combination with a larger excess of the diazo compound over the olefin substrate (4:1 vs 2:1 ratio) resulted in quantitative conversion of vinyl benzoate **4a** into the desired cyclopropanation product **5a** with excellent diastereo- and enantioselectivity (99.5:0.5 dr and er; Table 2, Entry 12). Notably, the same results could be achieved using a ∼10-fold lower catalyst loading of 0.1 mol %, indicating that DHP(H55A, V59A) is able to support over 1000 turnovers (TON) in this reaction under optimized conditions (Table 2, Entry 13). The DHP(H55A, V59A)-catalyzed reaction was determined to proceed rapidly, reaching 80% and >95% conversion over 10 and 30 min, respectively (Figure S2), with an initial rate of 35 turnovers per minute. Albeit in more modest yield, **5a** could be produced with high stereoselectivity (99.5:0.5 dr and er) also using cells expressing DHP(H55A, V59A), thus demonstrating the compatibility of this enzymatic transformation with whole-cell biotransformations (Table 2). Finally, using purified protein, a reaction with 15 mg of **4a** was carried out resulting in the isolation of **5a** in enantiopure form and in 46% isolated yield, demonstrating the scalability of this reaction.

### Substrate Scope

**Figure 3:**
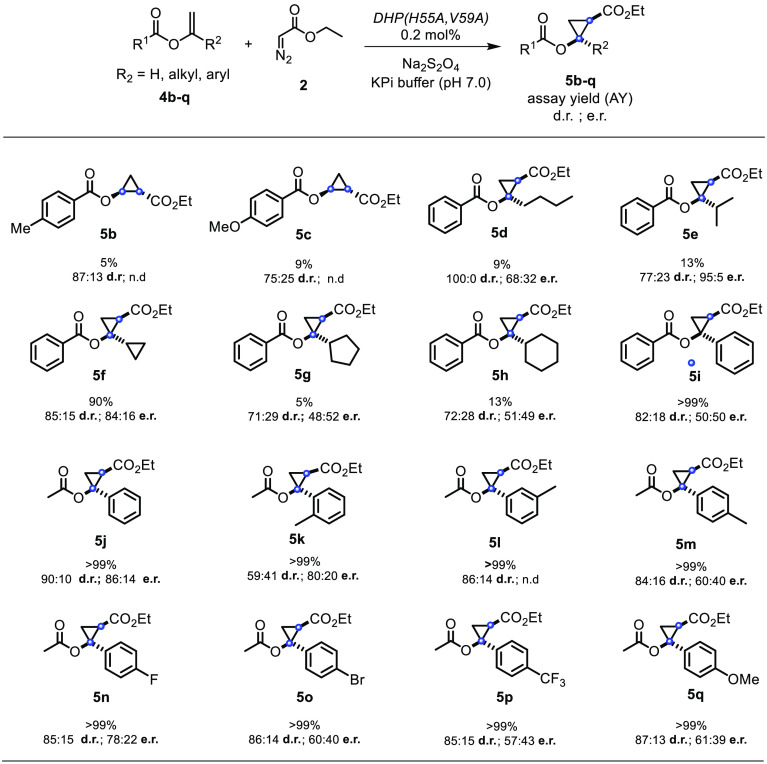
Substrate scope for DHP(H55A, V59A)-catalyzed cyclopropanation of vinyl esters. Reaction conditions: 20 μM enzyme, 2.5 mM substrate, 10 mM EDA, 10 mM dithionite, 10% MeOH, KPi buffer (50 mM, pH 7), anaerobic, 4 h. Assay yield, diastereomeric ratio (dr), and enantiomeric ratio (er) were determined by chiral GC-FID and SFC analysis using calibration curves with authentic (racemic) standards. For compounds **5d**–**5q**, only relative configuration was assigned. See the Experimental Section for further details on stereochemical assignments.

In the interest of examining the substrate scope of the DHP(H55A, V59A) variant, a range of different vinyl benzoates (**4b**–**4i**) were tested in the DHP(H55A, V59A)-catalyzed cyclopropanation reaction in the presence of EDA (Figure 3). The target substrates were prepared using modifications of reported procedures. Specifically, the benzoate derivatives **4b**–**4c** were synthesized via palladium(II) acetate catalyzed synthesis with carboxylic acid;^13^ α,α-disubstituted vinyl benzoates (**4d**–**4i**) were prepared by ruthenium-catalyzed addition of carboxylic acids to alkynes;^14^ and substituted vinyl acetates were synthesized via enolization of lithium enolate starting from acetophenones.^15^ Notably, all of these substrates could be successfully converted to the corresponding cyclopropanation products **5b**–**5i**, albeit in variable yields. Since the modest yield for **5b** and **5c** (5–9% AY) indicated a limited tolerance of the enzyme to substitutions at the level of the benzene ring in the olefin substrate, the benzoate group was kept invariant while varying the other substituent of the olefin. Accordingly, various α,α-disubstituted vinyl benzoates containing linear, branched, or cyclic alkyl groups at the α-position (**4d**–**4h**) were investigated. In general, the aliphatic substituents were tolerated to a moderate extent as indicated by the 5–13% yields obtained with **5d**–**5g**. As an exception, the cyclopropyl-substituted substrate **4f** was converted with high efficiency to the desired product **5f** (90% AY). In general, the DHP(H55A, V59A)-catalyzed reactions involving substrates with a linear, branched, and small cycloalkyl group proceeded with a good to high level of diastereoselectivity, ranging from 77:23 to >99:1 dr and from 68:32 to 95:5 er. The presence of larger cycloalkyl groups (**5g**, **5h**), on the other hand, showed good diastereoselectivity but no enantioselectivity. These results indicated that while the biocatalyst has a rather broad substrate scope, its stereoselectivity is dependent on the substrate.

Next, we tested the α-phenyl substituted vinyl benzoate **5i**, which was converted to the corresponding product **5i** with high efficiency (>99% AY) and good diastereoselectivity (82:12 dr), albeit with no enantioselectivity (Figure 3). Considering the stereochemical model previously reported for Mb-catalyzed styrene cyclopropanation with EDA,^11^ we reasoned that, in stark contrast to the high enantioselectivity observed for **4a**, the lack of enantioselectivity in the DHP-catalyzed reaction with α-phenylvinyl benzoate may arise from the presence of two α substituents of similar size (i.e., Ph vs OCOPh) across the double bond, which may prevent enzyme-mediated control on the facial selectivity of olefin attack to the heme-bound carbene. Based on these considerations, this substrate was “re-engineered” into α-phenylvinyl acetate **4j**, in which the benzoate group is replaced by a smaller yet functionally equivalent acetate group, thereby differentiating the size of the appended groups across the double bonds. Gratifyingly, DHP(H55A, V59A)-catalyzed cyclopropanation of **4j** led to the formation of the desired product **5j** in quantitative yield, higher diastereoselectivity (90:10 dr vs 82:18 dr), and significantly higher enantioselectivity (86:14 er vs 50:50 er) compared to **5i** (Figure 3), thus validating our substrate engineering strategy to enhance the enantioselectivity of this reaction.

On the basis of these results, a series of variously substituted 1-phenylvinyl acetate derivatives (**4k**–**4q**) were then investigated to further probe the substrate scope of the enzyme. *Ortho*-, *meta*-, and *para*-substituted derivatives (**4k**, **4l**, **4m**) were all efficiently processed by the enzyme (>99% AY), producing cyclopropanes **5k**–**5m** with moderate diastereo- and/or enantioselectivity. While **5l** was produced with good diastereoselectivity (86:14 dr), its enantiomeric ratio could not be determined due to difficulties in resolving the enantiomers with chiral gas or liquid chromatography. Lastly, the results from the reactions with **4n**–**4p** showed that both electron-withdrawing and electron-donating substitution on the phenyl ring are well tolerated by the enzyme, as judged by their quantitative conversion to **5n**–**5q**. In addition, these reactions were found to proceed with good diastereoselectivity (∼85:15 dr) but with moderate enantioselectivity (60:40 to 78:22 er; Figure 3), regardless of the electronic properties of the *para* substituent. Upon comparison with **5j** (and **5m**), these results also indicate that steric factors more than electronic factors are primarily responsible for the *para* substituent effect on enantioselectivity.

**Figure 4:**
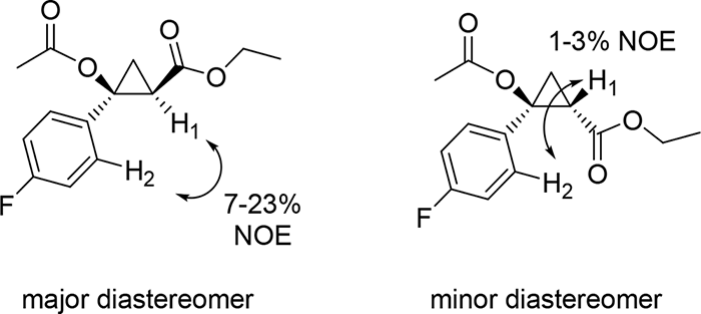
NOE experiments for analysis of stereochemical configuration.

Using **5n** as model compound, the diastereopreference of the enzyme was investigated via nuclear Overhauser effect (NOE) experiments (Figures 4 and S4). For the major diastereomer isolated from the enzymatic reaction, NOEs corresponding to 7% and 23% were observed between the H atom in alpha to the ester group (H_1_) and the H atom in the *ortho* position of the *p*-fluoro-phenyl group (H_2_) upon irradiation of H_1_ or H_2_, respectively. In contrast, much weaker NOEs (1–3%) were observed from the same experiments performed on the minor diastereomer (Figure 4 and S4). These results confirmed the *trans* relationship between the aryl group and the ethyl ester group across the cyclopropane ring (H_1_···H_2_ distance of 2.4–2.6 Å for *trans* isomer vs 3.6–3.7 Å for *cis* isomer based on molecular models), which is in line with the *trans* selectivity exhibited by the enzyme in the cyclopropanation of styrene with EDA (Table 1).

**Figure 5:**
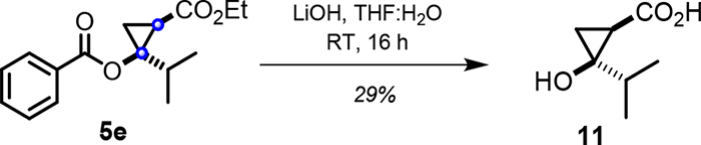
Hydrolysis of cyclopropanol ester **5e** to afford the cyclopropanol product.

Compounds **5a**–**5q** represent useful building blocks for further transformation, including the synthesis of unprotected cyclopropanols. As illustrated in Figure 5, cyclopropanol ester **5e** was readily hydrolyzed to yield the desired cyclopropanol product **11**.

### Mechanistic Investigations

**Figure 6:**
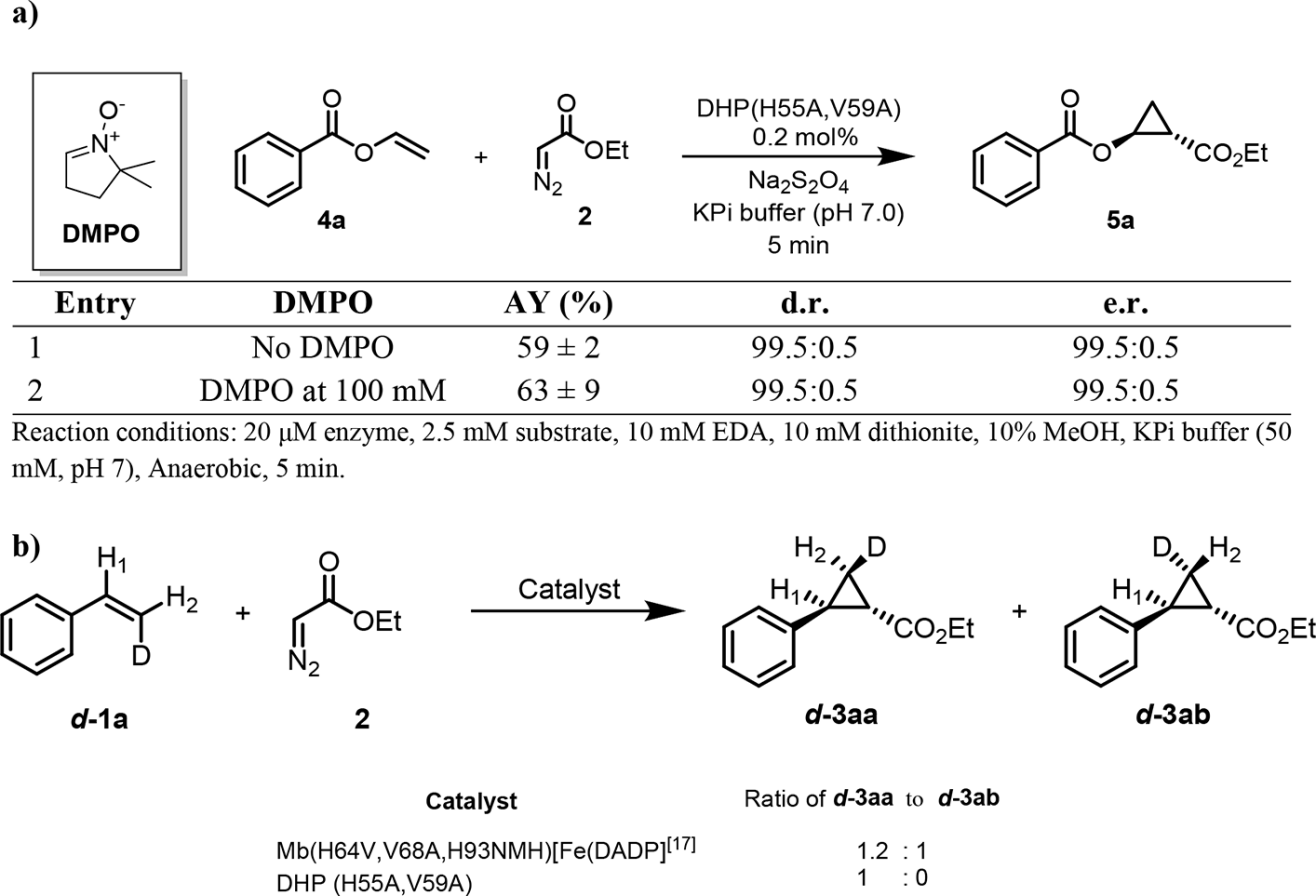
Radical spin trap (a) and isomerization (b) experiments toward the investigation of the mechanism of the DHP(H55A, V59A)-catalyzed cyclopropanation reaction. Reaction conditions: 20 μM enzyme, 2.5 mM substrate, 10 mM EDA, 10 mM dithionite, 10% MeOH, KPi buffer (50 mM, pH 7), anaerobic, 5 min.

We previously determined that the Mb-catalyzed cyclopropanation of styrene with EDA proceeds via a concerted insertion of the EDA-derived carbenoid into the olefinic double bond.^16^ In contrast, the artificial metalloenzyme Mb(H64V, V68A, H93NMH)[Fe(DADP)], which features a higher (more positive) redox potential and higher reactivity toward electron deficient olefins, was found to catalyze the same reaction via a radical-based, stepwise mechanism.^4g^ To gain insights into the mechanism of the DHP(H55A, V59A)-catalyzed cyclopropanation, we conducted the reaction with **4a** in the presence and in the absence of the radical spin trapping agent 5,5-dimethyl-1pyrroline *N*-oxide (DMPO), which we previously found to be useful for probing the radical mechanism of cyclopropanation reactions.^4g,16^ These experiments showed no inhibition of the reaction in the presence of DMPO, suggesting that a long-lived radical intermediate species is not formed (Figure 6A). To further investigate this point, we conducted the cyclopropanation reaction with the isotopically labeled *cis*-β-deuterostyrene and EDA. Previous studies from our group have shown that, in this reaction, enzymatic cyclopropanations involving a radical mechanism results in a *Z → E* rearrangement of the D label (Scheme S1).^4g^ In the presence of DHP(H55A, V59A), the reaction was found to proceed stereospecifically, yielding exclusively the isomer *d***-3aa** as determined by ^2^H NMR (Figure 6B). Thus, in agreement with the radical spin trapping experiments, these results support the notion that the DHP(H55A, V59A)-catalyzed cyclopropanation reaction involves a concerted carbene insertion into the olefin (Scheme S1), which is analogous to that of the engineered myoglobin variant Mb(H64V, V68A)^16^ but distinct from that of the cofactor-engineered artificial metalloenzyme Mb(H64V, V68A, H93NMH)[Fe(DADP)].^4g^ To evaluate the impact of the enzyme redox potential on the present cyclopropanation activity, we measured the redox potential (*E*°_Fe^3+^/Fe^2+^_) of wild-type DHP and the two most representative variants, i.e., DHP(H55A) and DHP(H55A, V59A), using a spectrophotochemical method.^17^ Wild-type DHP was determined to display a *E*°_Fe^3+^/Fe^2+^_ of 216 mV (Figure S1A), which is in excellent agreement with the literature value of 221 mV.^10^ For both engineered DHP variants, however, negligible heme reduction was observed even in the presence of dyes with the most positive redox potential compatible with this method (e.g., Bindschedler’s green (*E*_m_ = +224 mV); Figure S1B,C), indicating that the *E*°_Fe^3+^/Fe^2+^_ of these enzymes must be >300 mV and thus even higher than that of wild-type DHP. From these analyses and the mechanistic studies described above, we conclude that a more positive redox potential, but not a radical carbene transfer mechanism, is useful and beneficial for mediating cyclopropanation activity on this class of electrodeficient olefins.

### Other Carbene Transfer Reactions

**Figure 7:**
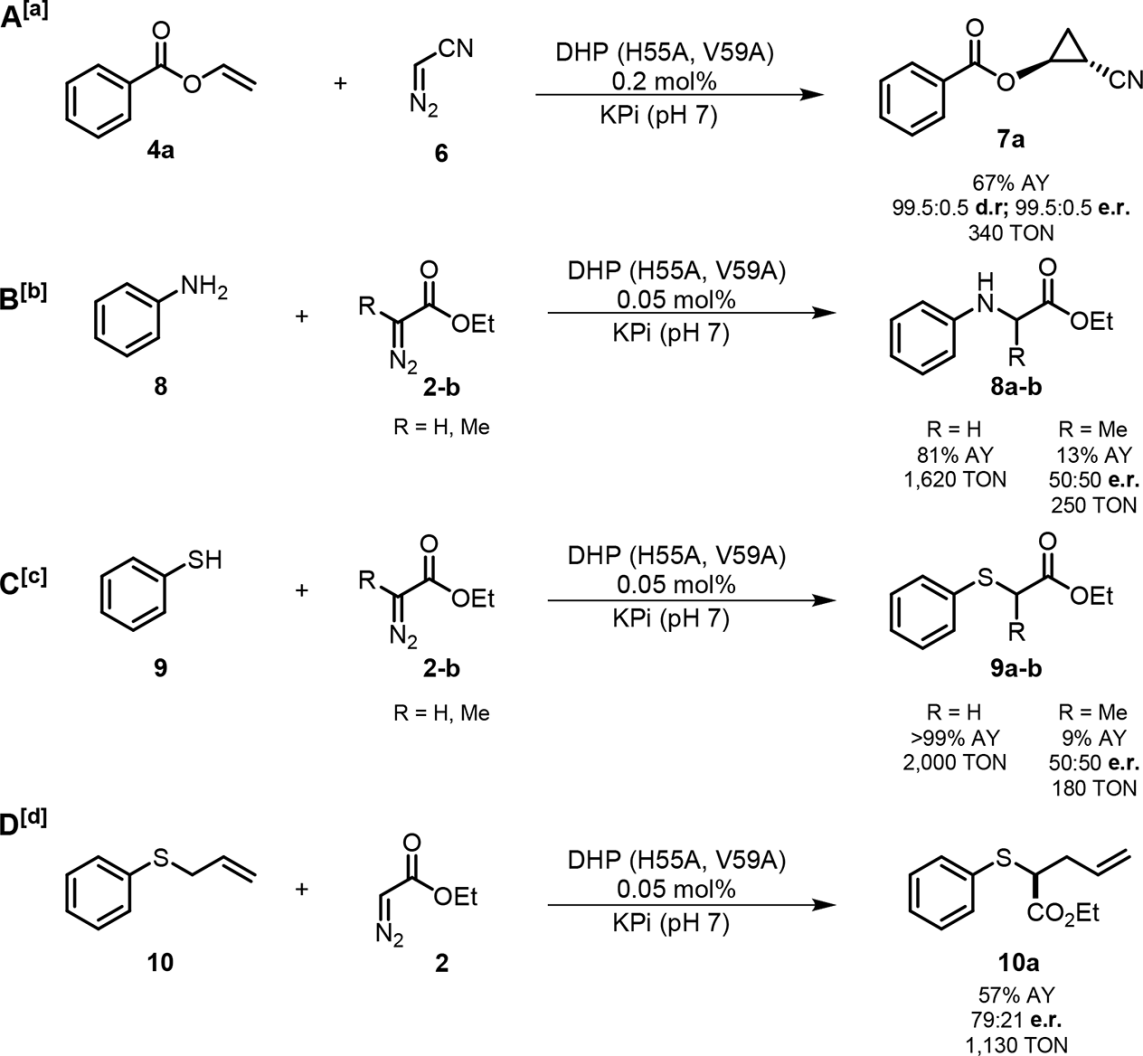
Catalytic activity and selectivity of DHP(H55A, V59A) in different carbene transfer reactions. Reaction conditions: ^[a]^20 μM enzyme, 10 mM substrate, 20 mM diazoacetonitrile. ^[b]^5 μM enzyme, 10 mM substrate, 10 mM diazo compound. ^[c]^5 μM enzyme, 10 mM substrate, 20 mM diazo compound. ^[d]^5 μM enzyme, 10 mM substrate, 20 mM EDA. All reactions were conducted with 10 mM Na_2_S_2_O_4_, in KPi buffer (50 mM, pH = 7.0), 10% MeOH, anaerobic, 6 h. Assay yield, diastereomeric ratio (dr), and enantiomeric ratio (er) were determined by chiral GC-FID and SFC analysis using calibration curves with authentic (racemic) standards.

Having established the functionality of DHP as cyclopropanation biocatalyst, we performed additional experiments to assess its reactivity in the context of other carbene transfer reactions, namely cyclopropanation with diazoacetonitrile as well as N–H carbene insertion,^18^ S–H carbene insertion,^19^ and Doyle–Kirmse reaction^20^ in the presence of EDA. In the presence of diazoacetonitrile (**6**) as the carbene source,^2c^ DHP(H55A, V59A) was found to catalyze the cyclopropanation of **4a** in high yield and with excellent diastereo- and enantioselectivity (67% AY, 99.5:0.5 dr and er) (Figure 7). Notably, organometallic catalysts such as Rh(OAc)_2,_ Cu(OTf)_2_, Fe(TPP)Cl, and CoTPP were unable to catalyze this reaction, highlighting the superior reactivity DHP in this challenging transformation. The cyano group enzymatically installed in **7a** can be useful for further diversification of the cyclopropane product.^2c^ Furthermore, using whole cells, 200 mg of **4a** was converted to give **7a** in 53% isolated yield, demonstrating the scalability of this reaction. As summarized in Figure 7, the DHP variant was found to have high activity in other carbene transfer reactions. In a reaction with aniline (**8**) and EDA (**2**), DHP(H55A, V59A) was found to produce the N–H insertion product **8a** in high yield (81%), supporting 1620 catalytic turnovers (TON) at a catalyst loading of merely 0.05 mol %. The DHP variant was also able to catalyze the alkylation of aniline in the presence of ethyl 2-diazopropanoate (EDP) as the carbene donor, resulting in the formation of **8b** with 13% yield and 250 TTN, albeit with no enantioselectivity. Similarly, the same biocatalyst is able to catalyze the S–H carbene insertion of both EDA and EDP with thiophenol to produce **9a** in quantitative yield and **9b** in 9% yield and with a TON value of 1620 and 180, respectively. Lastly, we tested DHP(H55A, V59A)’s ability to catalyze a Doyle–Kirmse reaction with allyl sulfide (**10**) and EDA (**2**). Notably, DHP(H55A, V59A) was found to produce **10a** in quantitative yield, with 1130 TON, and 79:21 er. The latter compares well with the enantioselectivity previously achieved with an engineered Mb variant, Mb(L29S, H64V, V68F), optimized for this reaction (86:14 er).^20^ Altogether, these findings demonstrate the functionality and versatility of DHP as a biocatalyst for different carbene transfer transformations.

## Conclusion

In summary, we have developed a biocatalytic strategy for the stereoselective synthesis of cyclopropanol esters, providing a new approach to access this valuable class of compounds. This methodology was made accessible by leveraging, for the first time, a dehaloperoxidase (DHP) enzyme as carbene transferase. Our results demonstrate that DHP is amenable to protein engineering to improve and fine-tune its catalytic activity and stereoselectivity in cyclopropanation reactions involving not only styrene but also more challenging vinyl ester substrates. In addition to cyclopropanations, our study shows that DHP is capable of supporting a variety of other carbene transfer reactions with good efficiency and selectivity, therefore adding to its multifunctional nature^9c,21^ and expanding the range of hemoproteins useful for these abiological transformations. We envision this metalloenzyme can provide a valuable scaffold for the development of other valuable biocatalytic transformations.

## Experimental Section

### General Information

All the chemicals and reagents were purchased from commercial suppliers (Sigma-Aldrich, Alfa Aesar, ACS Scientific, Acros, Ambeed, Combi-blocks) and used without any further purification. All dry reactions were carried out under argon in flame-dried glassware with magnetic stirring using standard gastight syringes, cannula, and septa. ^1^H and ^13^C NMR spectra were measured on Bruker DPX-500 (operating at 500 MHz for ^1^H and 125 MHz for ^13^C) or Bruker DPX-400 (operating at 400 MHz for ^1^H and 100 MHz for ^13^C), and ^19^F was measured on Bruker DPX-400 (operating at 375 MHz). Tetramethylsilane (TMS) (0 ppm) and/or CDCl_3_ (7.26 ppm) served as the internal standard for ^1^H NMR, CDCl_3_ was used as the internal standard (77.0 ppm) for ^13^C NMR, and trifluorotoluene served as the internal standard (−63 ppm) for ^19^F NMR. HRMS analyses were performed using a Q Exactive Plus mass spectrometer at the Proteomics Facility of the University of Rochester. Silica gel chromatography purifications were carried out using AMD silica gel 60 230–400 mesh. Thin layer chromatography (TLC) was carried out using Merck Millipore TLC silica gel 60 F254 glass plates.

### Molecular Cloning

pET22b(+) vector (Novagen) was used as the recipient plasmid vector for expression of all of the DHP variants. The DHP gene was prepared synthetically (Genscript) and cloned into pET22 vector with a C-terminal hexahistidine tag and under the control of an IPTG-inducible T7 promoter. Site-directed mutagenesis was performed using a QuickChange mutagenesis protocol as described previously.^1^ KOD Hot Start DNA Polymerase from Merck was employed, and chemically competent *E. coli* DH5α cells were used for plasmid amplification.

### Protein Expression and Purification

Engineered DHP variants were expressed in *E. coli* BL21(DE3) cells as follows. Briefly, cells were grown in TB medium (ampicillin, 100 mg/L) at 37 °C (170 rpm) until OD_600_ reached 0.9–1.2. Cells were then induced with 0.3 mM γ-aminolevulinic acid (ALA). After induction, cultures were shaken at 170 rpm and 37 °C and harvested after 18–20 h by centrifugation at 4000 rpm at 4 °C. After cell lysis by sonication, the proteins were purified by Ni-affinity chromatography. The lysate was transferred to a Ni-NTA column equilibrated with Ni-NTA Lysis Buffer. The resin was washed with 50 mL of Ni-NTA Lysis Buffer and then 50 mL of Ni-NTA Wash Buffer (50 mM KPi, 250 mM, NaCl, 20 mM imidazole, pH 8.0). Proteins were eluted with Ni-NTA Elution Buffer (50 mM KPi, 250 mM, NaCl, 250 mM histidine, pH 7.0). After elution, the proteins were buffer exchanged against 50 mM KPi buffer (pH 7.0 or 8.0) using 10 kDa Centricon filters. Dehaloperoxidase concentration was determined using an extinction coefficient (Fe(III)) ε_410_ = 116 mM^–1^ cm^–1^.

### Purified Protein Reactions

Reactions conducted during protein evolution were carried out at a 400 μL scale using BL21(DE3) whole cells expressing the DHP variant. In a typical procedure, the required volume of cells needed to produce a solution with the desired optical density (OD) was added to the crimp vial followed by the addition of potassium phosphate buffer (KPi, 50 mM, pH 7.0), producing a 400 μL cell solution. The reactions were initiated by addition of 20 μL of the vinyl ester (from a 100 mM stock solution in MeOH) and 20 μL of EDA (from an 400 mM stock solution in MeOH). The vials were capped and left under magnetic agitation for 3–16 h at room temperature. Upon completion of the desired time, the reactions were then analyzed outside of the chamber following the Product Analysis protocol shown below.

### Anaerobic Reactions

Analytical reactions were conducted with whole cells or purified with DHP variant (20 μM), 2.5 mM vinyl ester compound, 10 mM EDA and 10 mM sodium dithionite (Na_2_S_2_O_4_) using crimp vials producing a final volume of 400 μL. In a typical procedure, the selected vessel containing the corresponding amount of whole cells or purified DHP were introduced to an anaerobic chamber. Then, a corresponding amount of degassed potassium phosphate buffer (KPi, 50 mM, pH 7.0) was added to the vessel producing a 20 μM myoglobin solution followed by the addition of 40 μL of a freshly prepared sodium dithionite solution (100 mM stock solution) in KPi (50 mM, pH 7.0). The reactions were initiated by addition of 20 μL vinyl ester compound (from a 100 mM stock solution in MeOH), 20 μL EDA (from a 400 mM stock solution in MeOH). The vessels were capped and left under magnetic agitation for 3–16 h at room temperature. The reactions were then analyzed outside of the chamber following the Product Analysis protocol shown below.

### Product Analysis

After completion of the desired reaction times, the vessel (crimp vials) of the reaction was open to air. The reactions were then analyzed by addition of 20 μL of internal standard (50 mM benzodioxole in EtOH) to the reaction mixture, followed by extraction with 400 μL of CH_2_Cl_2_. After strong mixing, the solutions were spun down for 1 min at 14 000 rpm. The organic layer was extracted via pipet, placed in a GC vial containing a glass insert, and capped tight. The GC vials were analyzed by chiral GC-FID using a Shimadzu GC-2010 gas chromatograph equipped with an FID detector, and a chiral Cyclosil-B column (30 m × 0.25 mm × 0.25 μm film). **GC Separation Method 1**: 2 μL injection, injector temperature: 240 °C, detector temperature: 300 °C. Gradient: column temperature set at 130 °C for 2 min, then to 140 °C for 0.80 °C/min then 150 for 0.8 °C/min then at 180 for 0.60 °C/min then to 245 °C at 25 °C/min with a 3 min hold. Total run time: 82.6 min. **GC Separation Method 2**: 1 μL injection, injector temperature: 200 °C, detector temperature: 300 °C. Gradient: column temperature set at 100 °C for 3 min, then to 140 °C for 0.40 °C/min then to 245 °C at 25 °C/min with a 3 min hold. Total run time: 109.2 min. **GC Separation Method 3**: 1 μL injection, injector temperature: 240 °C, detector temperature: 300 °C. Gradient: column temperature set at 120 °C for 3 min, then to 150 °C for 0.80 °C/min then to 245 °C at 25 °C/min with a 2 min hold. Total run time: 46.3 min. Stereoselectivity determination was performed via chiral GC-FID. Calibration curves for quantification of the different cyclopropanation products were constructed with authentic standards prepared using Rh(OAc)_2_ as described in the synthetic procedures. All measurements were performed at least in duplicate. For each experiment, negative control samples containing no protein were included. Stereochemical assignments for **3a** and **5a** were made based on comparison of enzymatic products with authentic standards as described previously.^2b^^4g^ Stereochemical assignments for **5b**–**c** were made based on analogy with **5a**. For compounds **5d**–**5q**, only relative absolute configuration was determined. For compounds **5j**–**5q**, the relative configuration of the major enzymatic product was assigned based on NOE experiments with **5n**. For compounds **5d**–**5i**, the relative configuration of the major enzymatic product was tentatively assigned based on the results with **5a**.

### Reduction Potential Determination

These experiments were carried out using a slightly modified version of the UV–vis spectrochemical method reported by Raven and co-workers.^17^ Reactions were carried out on a 1 mL scale in a solution of KPi (50 mM, pH7) containing xanthine (30 mM stock solution), protein, dye, catalase (10 mg/mL stock solution), a mixture of glucose (1 M stock solution)/glucose oxidase (175 μ M stock solution), and xanthine oxidase (175 μ M stock solution). In a sealed vial, a solution of buffer containing glucose (5 mM final concentration), glucose oxidase (50 μ g/mL final concentration), and xanthine (300 μ M final concentration) was degassed by bubbling argon for 3 min. A buffered solution containing the DHP variant and dye was carefully degassed in a similar manner in a sealed cuvette (the concentration of the dye was adjusted by titration to give an absorbance which is approximately equal to that of the highest absorbance band in the protein spectra). The two solutions were then mixed via cannula, and then catalase (5 μ g/mL final concentration) and xanthine oxidase (50 nM final concentration) were added to initiate the two-electron oxidation of xanthine to uric acid and the corresponding reduction of protein and dye. The reactions were monitored by UV–vis spectrophotometry, and the data were plotted. The reduction potential was determined by adding the standard reduction potential of dye to the value of the *y* intercept obtained by fitting the data to the Nernst equation (eq 1).
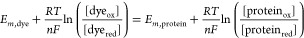
1

The absorbance values corresponding to the protein (based on the Soret band of the oxidized form) and the dye (Figure S1) were used to determine the ratio of concentrations of the oxidized (ox) to reduced (red) forms of both protein and dye at each stage of the experiment (eq 2).
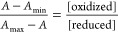
2

### Synthesis of Substituted Vinyl Benzoates (**4b**–**4c**) via Palladium(II) Acetate and Carboxylic Acids (Procedure A)

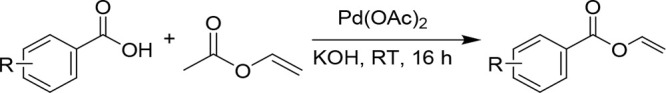



In a 100 mL flame-dried round-bottom flask containing a dry magnetic stir bar, benzoic acids (2.1 mmol, 1.0 equiv), Pd(OAc)_2_ (0.36 mmol, 0.16 equiv), and KOH (0.42 mmol, 0.2 equiv) were added followed by 20 mL of vinyl acetate. The reaction mixture was left to stir at room temperature for 16 h. After 16 h, the reaction mixture was filtered using Celite and the solvent was then removed under reduced pressure, and the crude reaction mixture was purified via flash column chromatography, using silica gel and a 2% EtOAc/hexanes solvent system.

### Synthesis of Substituted Vinyl Benzoates (**4d**–**4i**) via Ru-Catalyzed Addition of Carboxylic Acids to Alkynes (Procedure B)

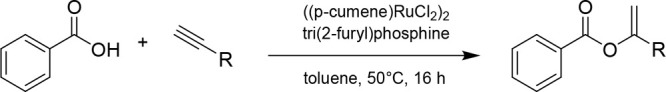



In a 100 mL flame-dried round-bottom flask containing a dry magnetic stir bar, benzoic acid (5 mmol, 1.0 equiv), ((*p*-cumene) RuCl_2_)_2_ (0.02 mmol, 0.004 equiv), tri(2-furyl) phosphine (0.04 mmol, 0.008 equiv), and sodium carbonate (0.08 mmol, 0.016 equiv) were added followed by 20 mL of toluene and the alkyne (6.5 mmol, 1.3 equiv). The reaction mixture was heated to 50 °C in an oil bath for 16 h. The solvent was then removed under reduced pressure, and the crude reaction mixture was purified via flash column chromatography, using silica gel and a 2% EtOAc/hexanes solvent system.

### Synthesis of Substituted Vinyl Acetates (**4j**–**4q**) via Enolization of Lithium Enolate (Procedure C)

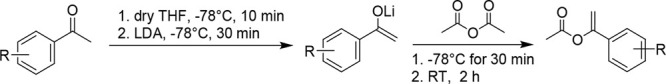



To a flame-dried 2 neck round-bottom flask containing a dry magnetic stir bar under argon atmosphere, a solution of acetophenone (3 mmol, 1.0 equiv) in anhydrous THF was added using a gastight syringe to the flask and left to cool to −78 °C for 10 min. LDA (2 M in THF) was added dropwise using gastight syringe. Afterwards, the mixture was left to stir at −78 °C for 30 min. Acetic anhydride was added to the resulting mixture and was left to stir for an additional 30 min at −78 °C. After 30 min, the resulting mixture was left to stir at room temperature for 45 min. Saturated NaHCO_3_ (100 mL) was poured into the reaction mixture and extracted with EtOAc (60 mL). The combined organic layers were washed with brine and dried over NaSO_4_, and the solvent was removed under reduced pressure. The crude mixture was purified by column chromatography on silica gel with a 50% DCM/hexanes solvent system.

### Synthesis of Racemic Standards (Procedure D)

In a 10 mL flame-dried round-bottom flask, Rh_2_(OAc)_4_ (1.4 × 10^–5^ mol) and substrate (3.4 × 10^–3^ mol) were dissolved in anhydrous CH_2_Cl_2_ (2.0 mL) under argon. A solution of EDA (6.8 × 10^–4^ mol) in CH_2_Cl_2_ (4.0 mL) was added dropwise via syringe pump over 1 h. Upon full addition of EDA, the reaction was stirred at room temperature overnight. The solvent was then removed under reduced pressure, and the crude reaction mixture was purified by flash chromatography (silica gel, hexane:EtOAc 95:5). The cyclopropanation products were obtained as their racemic *cis* and *trans* isomers.

### Synthesis of (1*S*,2*R*)-2-Cyanocyclopropyl Benzoate (Procedure E)

To a 250 mL round-bottom flask, whole cell DHP(H55A, V59A) was added followed by the addition of 1.35 mmol vinyl benzoate and 2.7 mmol diazoacetonitrile. The reaction was left to stir for 4 h. The organic layer was extracted using CH_2_Cl_2_ and dried over NaSO_4_. The solvent was removed under reduced pressure, and the crude mixture was purified by column chromatography on silica gel with a hexane:EtOAc 95:5 solvent system.

### Synthesis of (1*R*,2*R*)-2-Hydroxy-2-isopropylcyclopropane-1-carboxylic Acid

Cyclopropane product **5e** (0.047 mmol) was dissolved in 1:1 THF:H_2_O and added with 2.2 equiv of LiOH. The reaction mixture was stirred for 16 h. The reaction was quenched with 3 M HCl, and the organic later was extracted using EtOAc. The solvent was removed under reduced pressure, and the crude mixture was purified by column chromatography on silica gel first with 25 mL of 1:1 hexane:EtOAc solvent system and then with 100 mL 1:1 hexane:EtOAc and 1 mL acetic acid.

## Data Availability

The data underlying this study are available in the published article and its online Supporting Information.

## Supporting Information Available

Supplementary tables and figures, chiral GC and SFC chromatograms, synthetic procedures, compound characterization data, NMR spectra (PDF)
