# Supplementary material for: Dehaloperoxidase Catalyzed Stereoselective Synthesis of Cyclopropanol Esters
Source: J Org Chem. 2022 Dec 21;88(12):7630–40. doi: 10.1021/acs.joc.2c02030 (PMC10277220; doi:10.1021/acs.joc.2c02030)
Supplement: Supplementary file 1 — jo2c02030_si_001.pdf [file jo2c02030_si_001.pdf]

Supporting Information for

Dehaloperoxidase Catalyzed Stereoselective Synthesis of Cyclopropanol Esters

Mary G. Siriboe,[a] David A. Vargas,[a,b] and Rudi Fasan*,[a]

[a] *Department of Chemistry, University of Rochester, 120 Trustee Road, Rochester, New York, 14627, United States*

[b] *Current affiliation: Process Research and Development, Merck & Co., Inc., Rahway, NJ, 07065, USA.*

Correspondence should be addressed to R.F. (rfasan@ur.rochester.edu)

Table of Contents

Supplementary Tables	S2-S4
Supplementary Figures	S5-S24
Supplementary Scheme	S25
Compound Characterization	S26-S39
NMR Spectra	S40-S79
Reference	S80

Table S1. Tables with Whole Cells Reaction with DHP mutants H55X, V59A

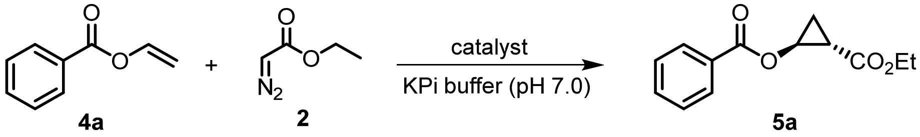

Entry	Catalyst	OD_{600}	AY (%)	dr_{trans}	er_{trans}
1	DHP (H55A,V59A)	10	12	99.5:0.5	99.5:0.5
2	DHP (H55A,V59A)	20	14	99.5:0.5	99.5:0.5
3	DHP (H55A,V59A)	40	17	99.5:0.5	99.5:0.5
4	DHP (H55A,V59A)	60	21	99.5:0.5	99.5:0.5
5	DHP (H55A,V59A)	80	16	99.5:0.5	99.5:0.5
6	DHP (H55A,V59A)	100	18	99.5:0.5	99.5:0.5
7	DHP (H55G,V59A)	20	1%	99.5:0.5	n.d
8	DHP (H55G,V59A)	40	3%	99.5:0.5	n.d
9	DHP (H55G,V59A)	60	4%	99.5:0.5	n.d
10	DHP (H55V,V59A)	20	0%	-	-

[a] Reaction Conditions: 20 mM Catalyst, 10 mM vinyl benzoate, 20 mM EDA, 10 mM dithionite, 5% EtOH, anaerobic 4 h

Table S2. Optimization of the reaction conditions for DHP(H55A,V59A) catalyzed cyclopropanation of vinyl benzoate with EDA. Reaction Conditions: 5% organic co-solvent in KPi buffer (pH 7), 10 mM sodium dithionite, anaerobic conditions, 4 hours.

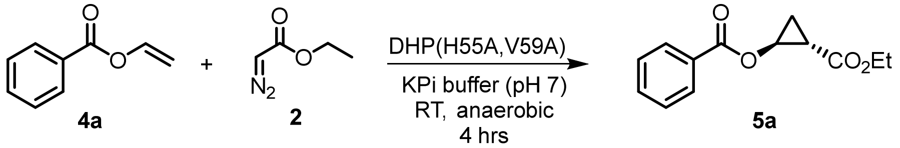

No.	Cat. Loading	Sub. Con. (mM)	EDA Con.(mM)	Organic Co-solvent	AY (%)	pH	dr_{trans}	er_{trans}
1	20	10	20	EtOH	35	7	99.5:0.5	99.5:0.5
2	20	5	10	EtOH	36	7	99.5:0.5	99.5:0.5
3	20	2.5	5	EtOH	53	7	99.5:0.5	99.5:0.5
4	20	10	20	EtOH	26	6	99.5:0.5	99.5:0.5
5	20	10	20	EtOH	25	8	99.5:0.5	99.5:0.5
6[a]	20	10	20	EtOH	30	9	99.5:0.5	99.5:0.5
7	40	10	20	EtOH	29	7	99.5:0.5	99.5:0.5
8	60	10	20	EtOH	26	7	99.5:0.5	99.5:0.5
9	20	10	20	EtOH	23	7	99.5:0.5	99.5:0.5
10	20	5	10	EtOH	24	7	99.5:0.5	99.5:0.5
11	20	1.25	2.5	EtOH	51	7	99.5:0.5	99.5:0.5
12	20	5	2.5	EtOH	55	7	99.5:0.5	99.5:0.5
13	20	2.5	5	EtOH	17	7	99.5:0.5	99.5:0.5
14	20	2.5	5	MeCN	40	7	99.5:0.5	99.5:0.5
15	20	2.5	5	DMSO	44	7	99.5:0.5	99.5:0.5
16	20	2.5	5	MeOH	67	7	99.5:0.5	99.5:0.5
17[c]	20	2.5	5	MeOH	45	7	99.5:0.5	99.5:0.5
18[c]	20	1.25	2.5	MeOH	53	7	99.5:0.5	99.5:0.5
19	20	5	2.5	MeOH	66	9	99.5:0.5	99.5:0.5
20	20	2.5	5	MeOH	19	9	99.5:0.5	99.5:0.5
21	20	2.5	5	MeOH	27	7	99.5:0.5	99.5:0.5
22[d]	20	2.5	5	MeOH	80	7	99.5:0.5	99.5:0.5
23	20	2.5	5	Isopropanol	30	7	99.5:0.5	99.5:0.5
24	20	2.5	5	THF	72	7	99.5:0.5	99.5:0.5
25	20	2.5	5	DMF	17	7	99.5:0.5	99.5:0.5
26[d]	20	2.5	5	MeOH	80	7	99.5:0.5	99.5:0.5
27[d]	20	2.5	10	MeOH	>99	7	99.5:0.5	99.5:0.5

[a]Sodium borate buffer at pH 9. [b] at 0°C [c] slow addition of EDA [d] 10% organic co-solvent

Table S3. Sequence of the oligonucleotides used for the preparation of the dehaloperoxidase variants.

Primer	Sequence
DHP Wt Fwd	AAAAACATATGGGTTTCAAGCAAGACATCGCGA
DHP Wt Rev	AAAAACTCGAGTTTCATACCCGCGCTGCT
XhoI	GGCTTTGTTAGCAGCCGGAT
DHP F24A Fwd	CATTTTCCTGGCGGCGCTGAATAAATAC
DHP F35A Fwd	CGTCGTTACGCGAAGAACTATG
DHP Y38A Fwd	CTTCAAGAACGCGGTGGGTAAAAG
DHP H55A Fwd	GAAATTCGGCGATGCCACCGAGAAG
DHP M63A Fwd	GTTTAACCTGGCGATGGAAGTTG
DHP V59A Fwd	CACCGAGAAGGCGTTTAACCTG
DHP L100A Fwd	CTTCGAGAAAGCGTTTGTGGCG

Figure S1. Spectrophotometric redox potential determination for wild-type DHP and the DHP variants. Representative UV-vis spectra during the determination of the $Fe^{3+/2+}$ reduction and Nerst plot.

A) Wild-type DHP (Dye: Bindschedler's green). UV-vis spectra (top) and Nernst plot generated from UV-vis spectra (bottom). The calculated redox potential is 216 ± 5 mV (n =3).

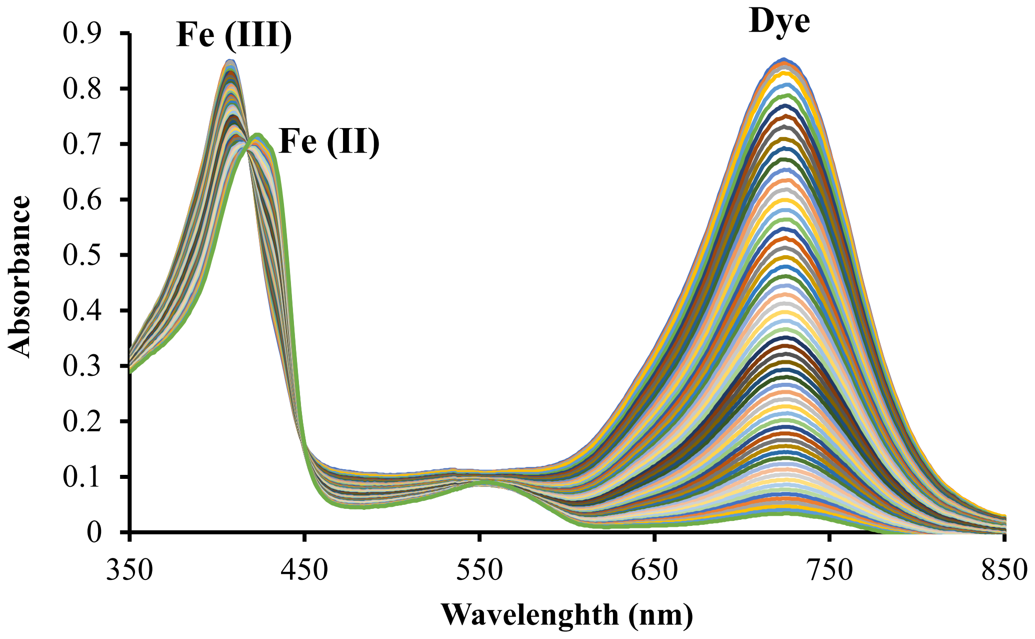

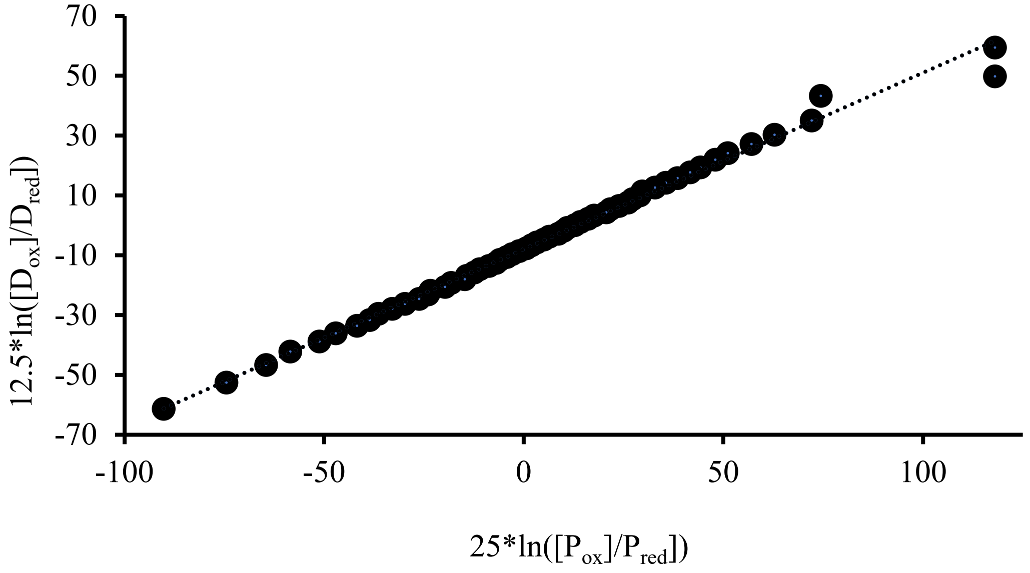

B) DHP(H55A) variant (Dye: Bindschedler's green). No reduction of the protein was observed while the dye was reduced, indicating that the E° Fe^{3+}/Fe^{2+} of the metalloprotein must be >300 mV.

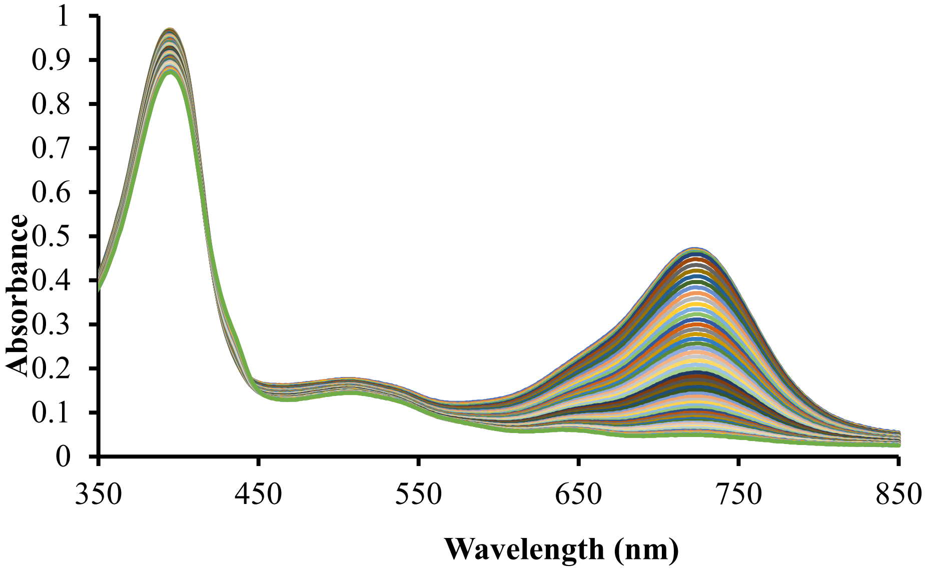

C) DHP(H55A,V59A) variant (Dye: Bindschedler's green). No reduction of the protein was observed while the dye was reduced, indicating that the E° Fe^{3+}/Fe^{2+} of the metalloprotein must be >300 mV.

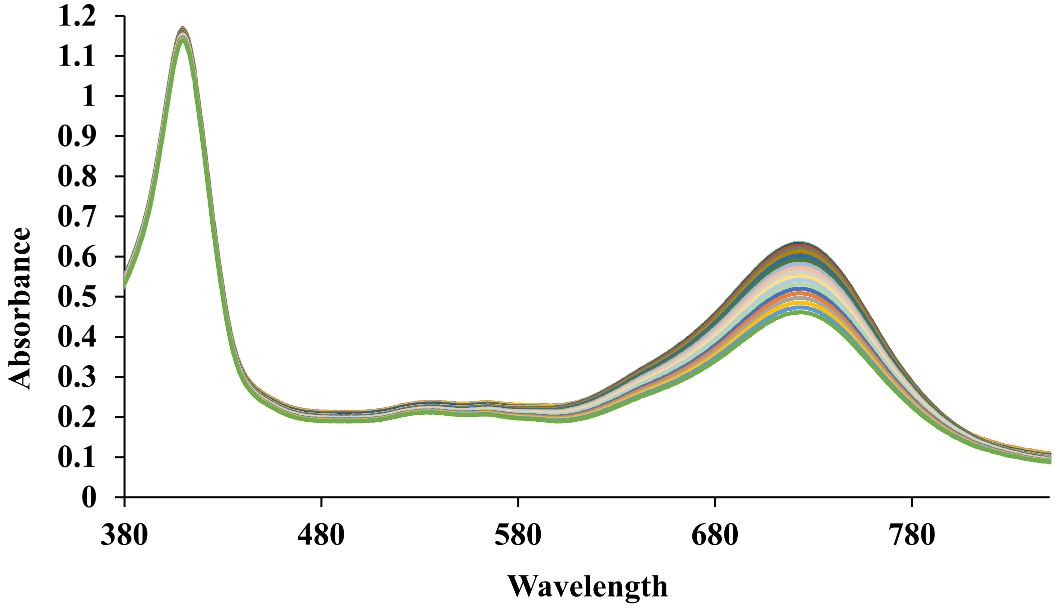

Figure S2. Kinetic analysis of DHP(H55A,V59A)-catalyzed cyclopropanation of vinyl benzoate with EDA. Reaction Conditions: 20 µM enzyme, 2.5 mM substrate, 10 mM EDA, 10 mM dithionite, 10% MeOH, KPi buffer (50 mM, pH 7), Anaerobic. Assay yield, were determined by chiral GC-FID analysis using calibration curves with authentic (racemic) standards

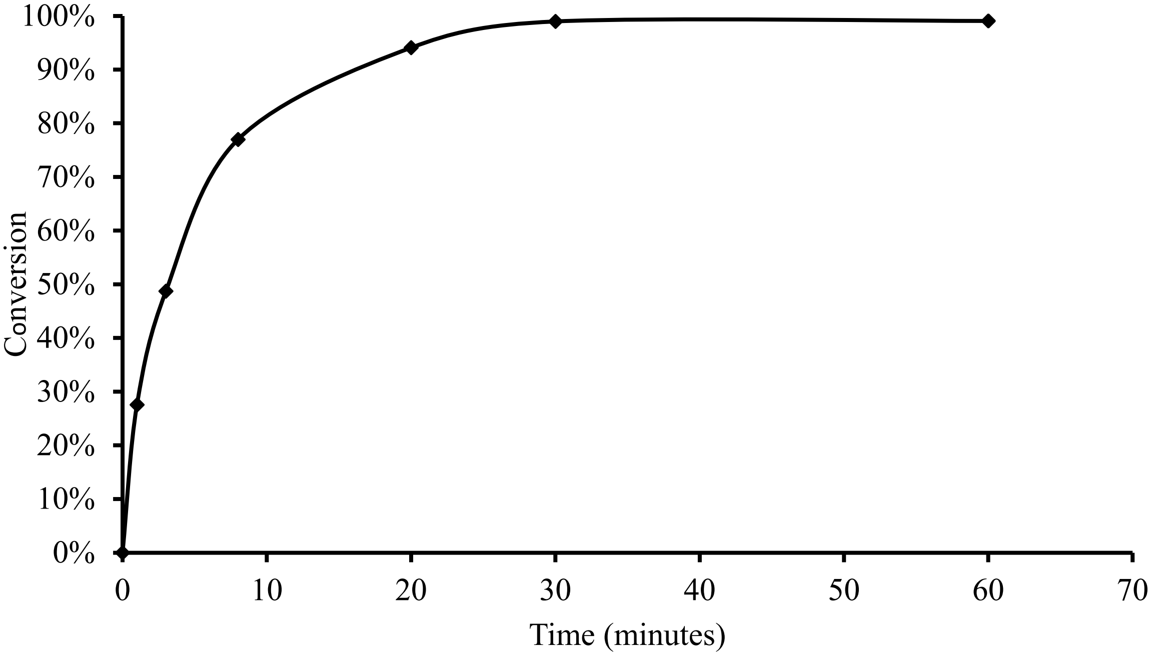

Figure S3. Inhibition Experiments with DMPO in the cyclopropanation reaction catalyzed by DHP(H55A,V59A). Reaction Conditions: 20 μM enzyme, 2.5 mM vinyl benzoate 10 mM EDA, 10 mM Na_2S_2O4, 5 mins, anaerobic, and room temperature. [a] with 100mM DMPO. All reactions were performed in triplicates.

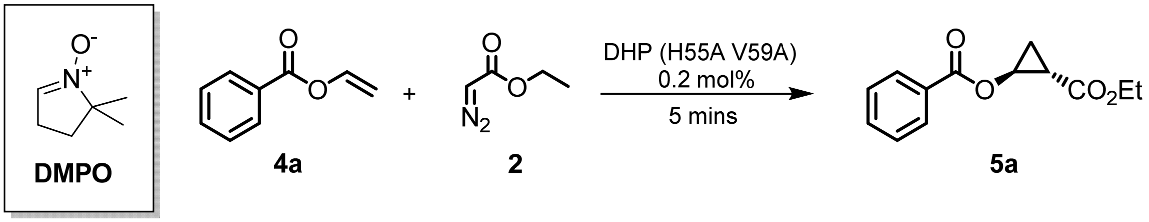

Entry	Variant	DMPO	AY (%)	de_{trans} (%)	ee_{trans} (%)
1	DHP (H55A V59A)	No	59(±0.02)	99	99
2[a]	DHP (H55A V59A)	Yes	63(±0.09)	99	99

Figure S4. NOE experiment with ethyl 2-acetoxy-2-(4-fluorophenyl)cyclopropane-1-carboxylate (**5n**). a) ^{1}H NMR spectrum of ethyl (1R,2S)-2-acetoxy-2-(4-fluorophenyl) cyclopropane-1-carboxylate; b) NOE spectrum of **5n** (major diastereomer) measured after irradiation of H_2 proton. c) NOE spectrum of **5n** (major diastereomer) measured after irradiation of the H_1 proton. NOE difference spectra yielded a NOE of 7-23% for the major diastereomer of **5n** and an NOE of 1-3% for the minor diastereomer of **5n**, consistent with *trans* relationship between the aryl group and the ethyl ester group across the cyclopropane ring.

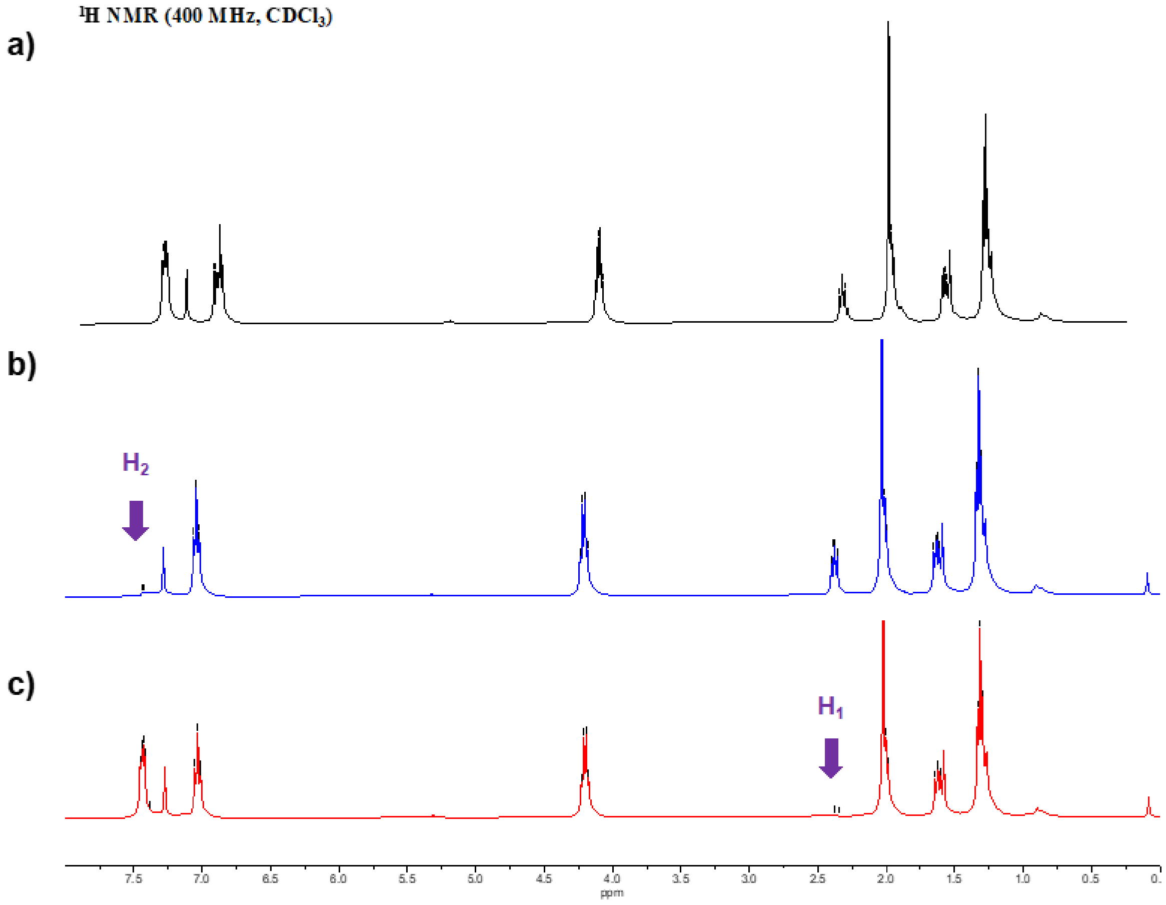

Figure S5. ^{2}H-NMR spectra indicating d-3a-a product produced from DHP(H55AV59A)

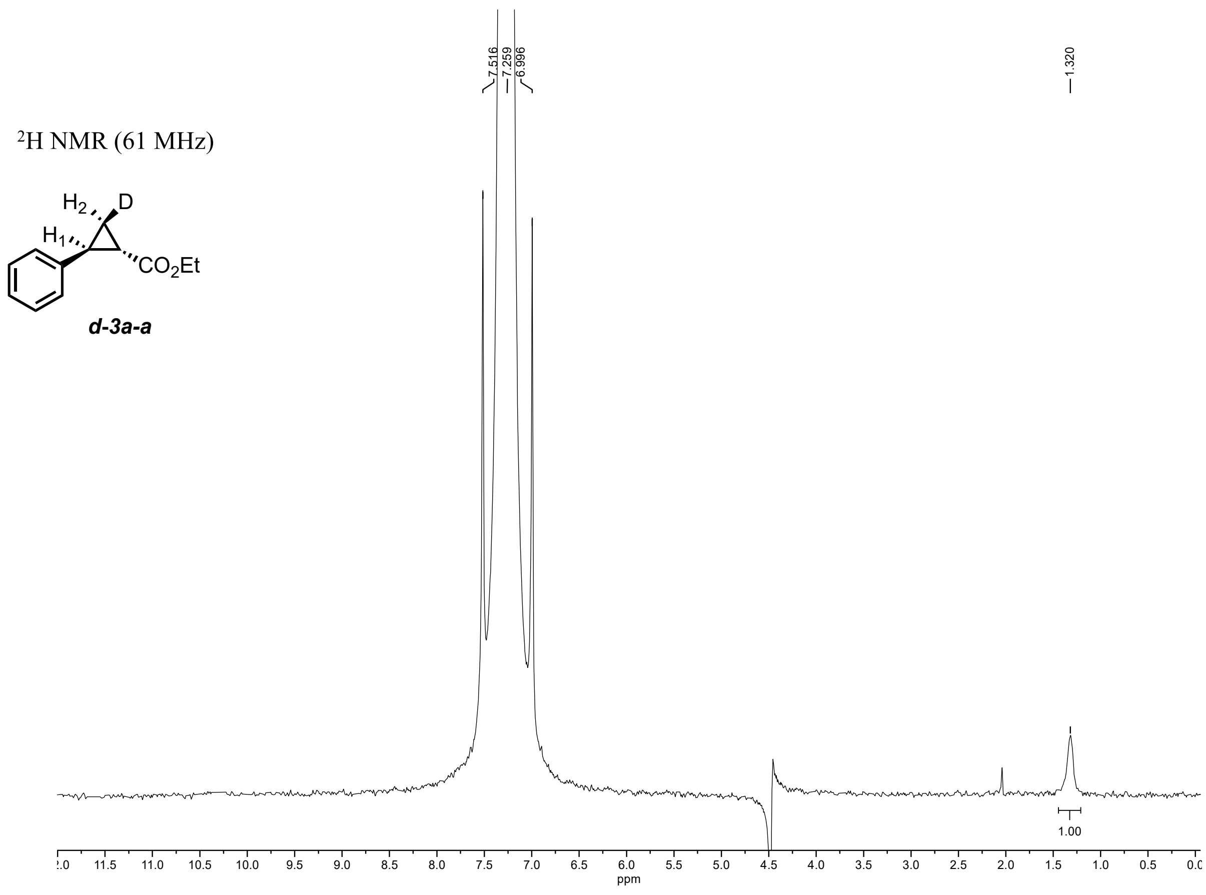

Figure S6. GC and SFC chromatograms for determination of diastereomeric and enantiomeric excess in the cyclopropanation reactions of vinyl benzoate and acetate substrates catalyzed by DHP(H55A,V59A). Reference racemic samples were prepared using $Rh_2(OAc)_4$ as catalyst described in the experimental procedures. GC method used for separation is described for each substrate, see **Product Analysis** section for details.

a) **ethyl (1S,2S)-2-phenylcyclopropane-1-carboxylate** (**3a**) GC analysis for diastereomeric and enantiomeric determination of compound **3a** using GC Separation Method 3. Racemic (*top*) and enzymatically (*bottom*) generated products are shown below

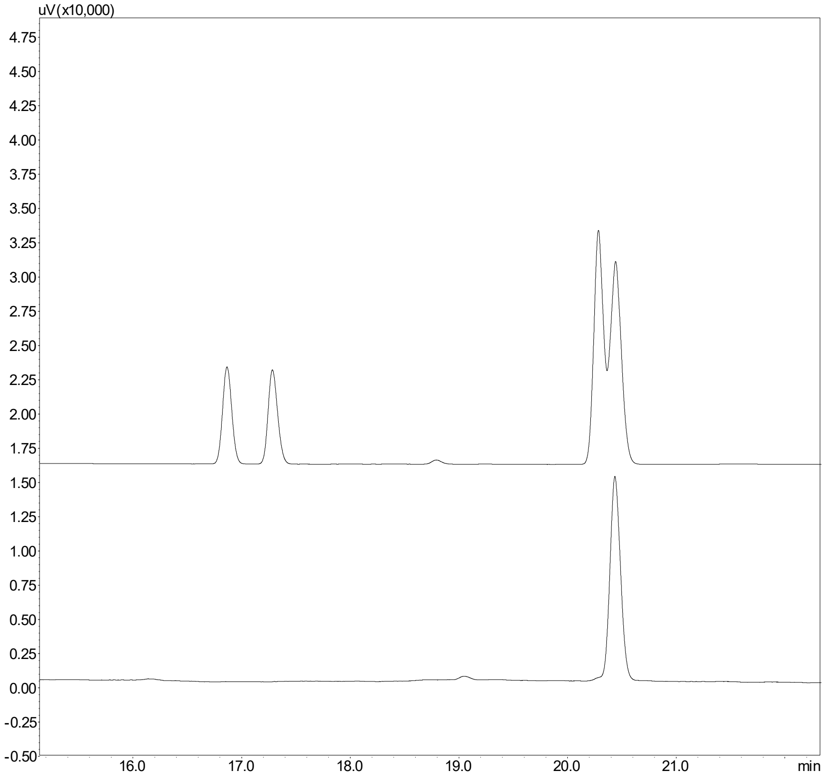

b) **(1S,2S)-2-(ethoxycarbonyl)cyclopropyl benzoate (5a)** SFC analysis of trans and cis isomers **5a** (left) and enzymatically produced **5a** product. Compound **5a** was analyzed using Daicel Chiralpak IF column and 2% of iPrOH

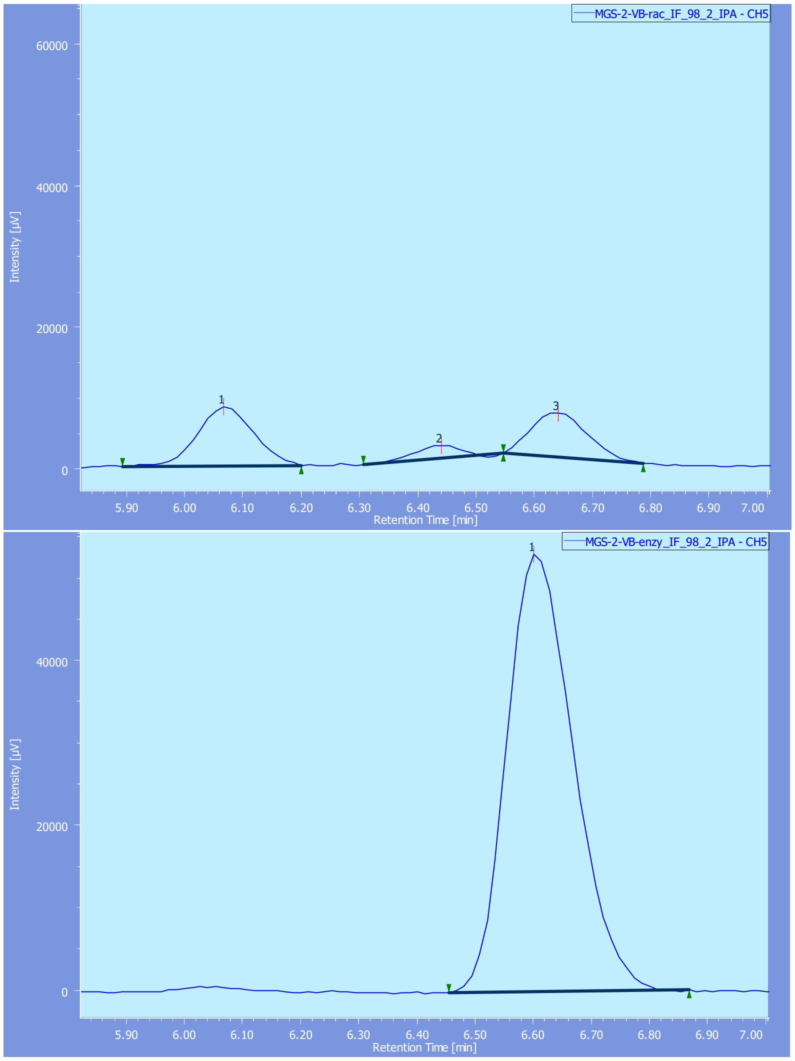

c) **(1*S*,2*R*)-1-butyl-2-(ethoxycarbonyl)cyclopropyl benzoate (5d).** GC analysis for diastereomeric and enantiomeric determination of compound **5d** using GC Separation Method 1. Racemic (*top*) and enzymatically (*bottom*) generated products are shown below.

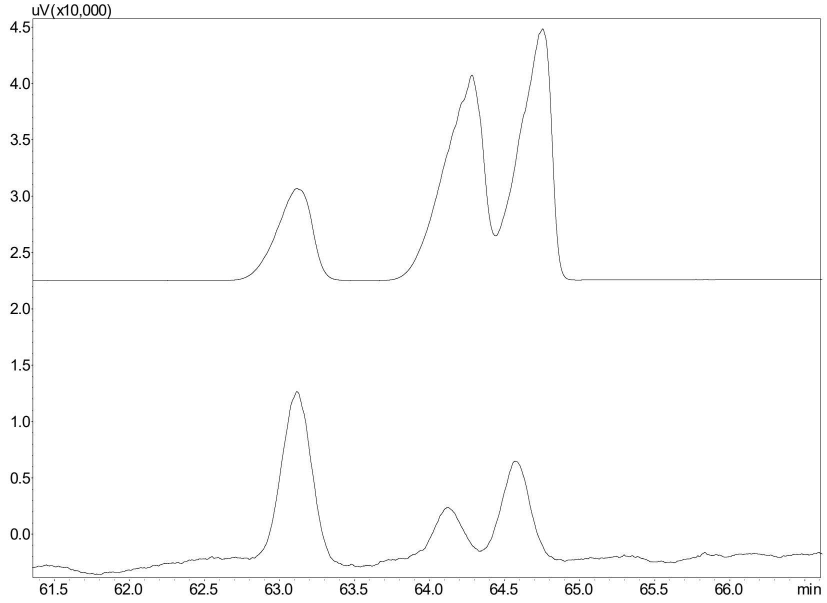

d) **(1*R*,2*R*)-2-(ethoxycarbonyl)-1-isopropylcyclopropyl benzoate (5e).** GC analysis for diastereomeric and enantiomeric determination of compound **5e** using GC Separation Method 1. Racemic (*top*) and enzymatically (*bottom*) generated products are shown below.

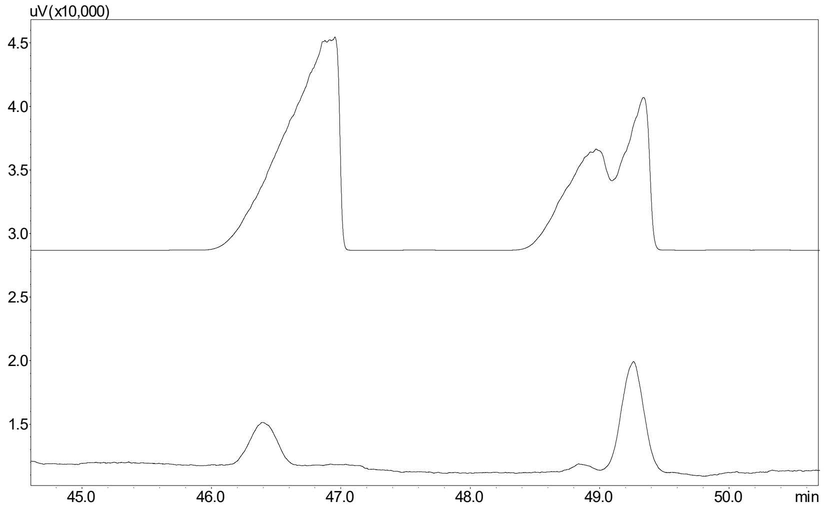

e) **(1*R*,2*R*)-2-(ethoxycarbonyl)-[1,1'-bi(cyclopropan)]-1-yl benzoate (5f).** GC analysis for diastereomeric and enantiomeric determination of compound **5h** using GC Separation Method 1. Racemic (*top*) and enzymatically (*bottom*) generated products are shown below.

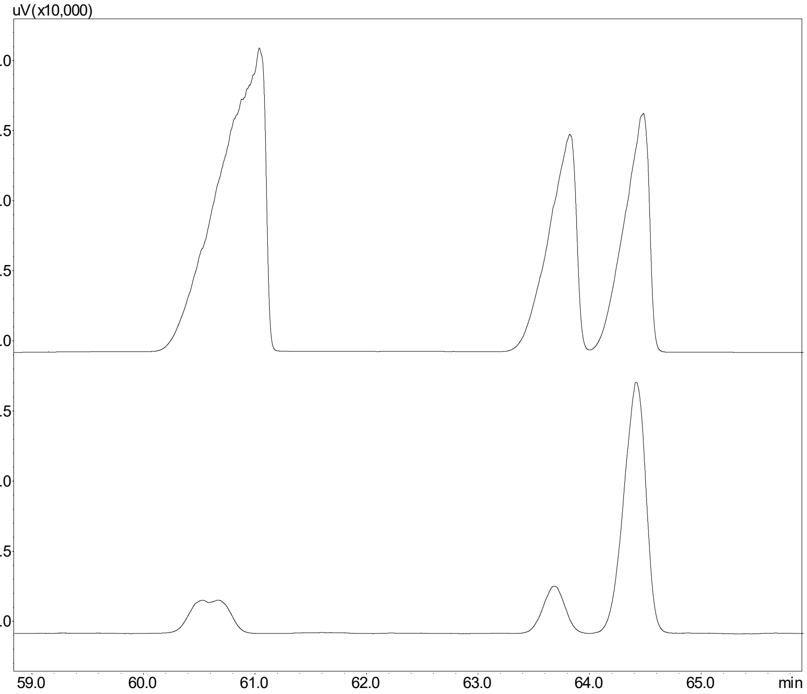

f) **(1R,2R)-1-cyclopentyl-2-(ethoxycarbonyl)cyclopropyl benzoate (5g)** GC analysis for diastereomeric and enantiomeric determination of compound **5g** using GC Separation Method 1. Racemic (*top*) and enzymatically (*bottom*) generated products are shown below.

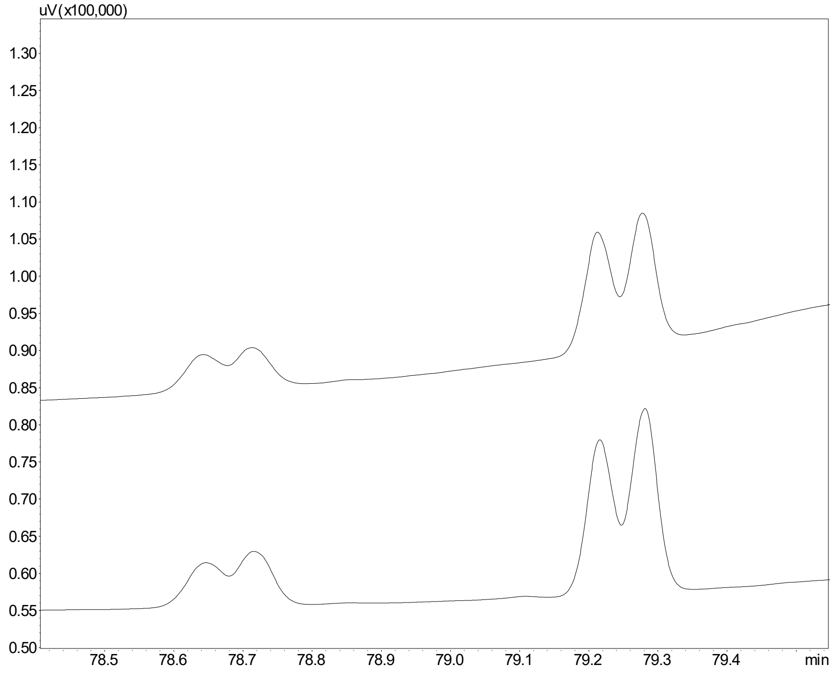

g) **(1S,2R)-2-(ethoxycarbonyl)-1-phenylcyclopropyl benzoate (5i)**. SFC analysis for diastereomeric and enantiomeric determination of compound **5i**. Racemic (*top*) and enzymatically (*bottom*) generated products are shown below. Compound **5i** was analyzed using Daicel Chiralpak IF column and 10% of iPrOH

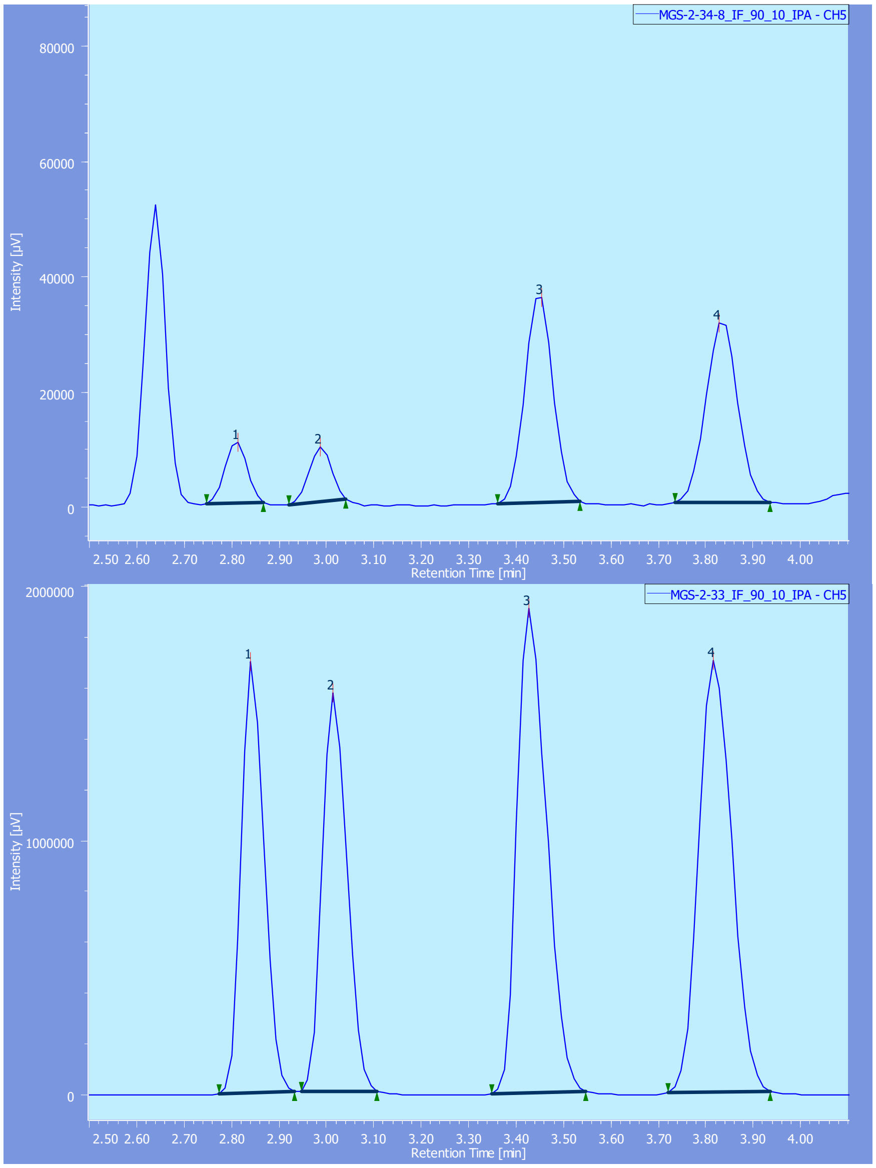

h) **Ethyl (1R,2S)-2-acetoxy-2-phenylcyclopropane-1-carboxylate (5j)** GC analysis for diastereomeric and enantiomeric determination of compound **5j** using GC Separation Method 2. Racemic (*top*) and enzymatically (*bottom*) generated products are shown below.

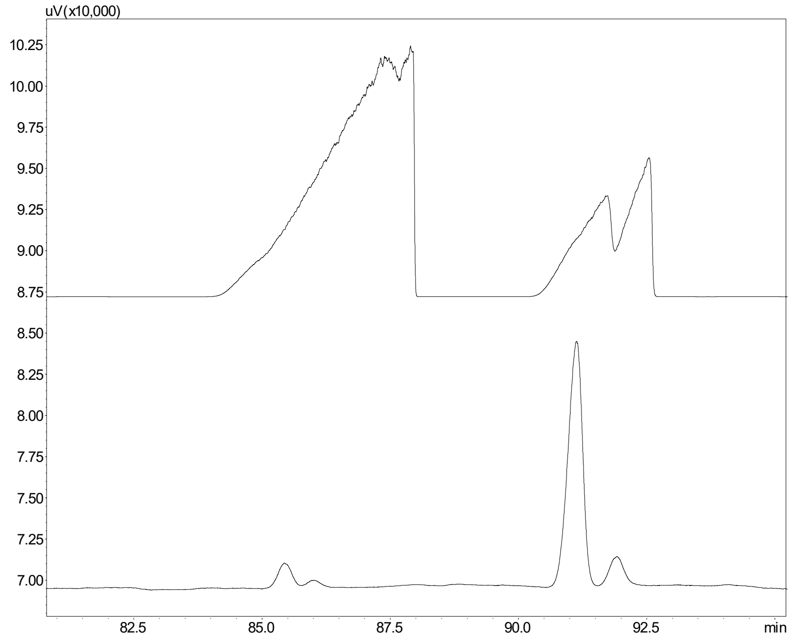

i) **Ethyl (1R,2S)-2-acetoxy-2-(o-tolyl)cyclopropane-1-carboxylate (5k)**. SFC analysis for diastereomeric and enantiomeric determination of compound **5k**. Racemic (*top*) and enzymatically (*bottom*) generated products are shown below. Compound **5k** was analyzed using Daicel Chiralpak IF column and 2% of iPrOH

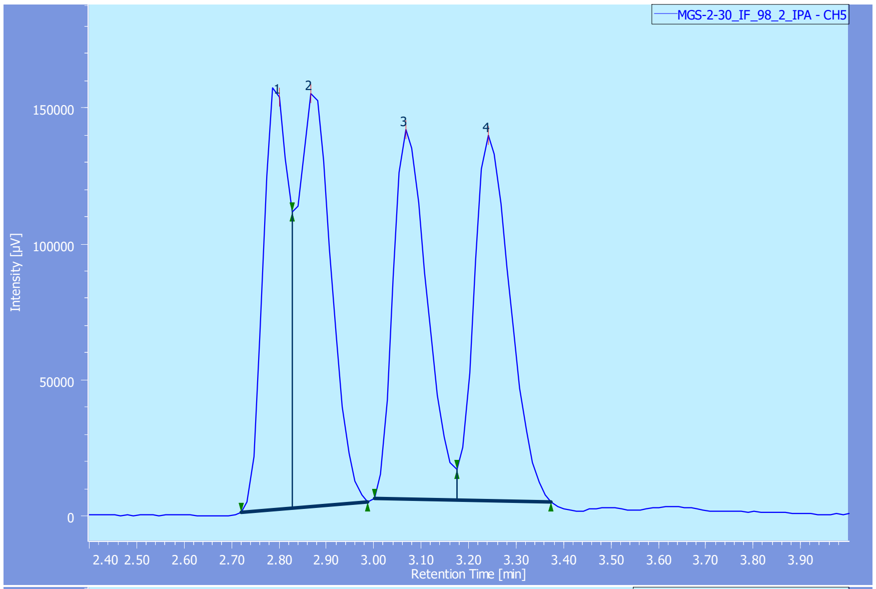

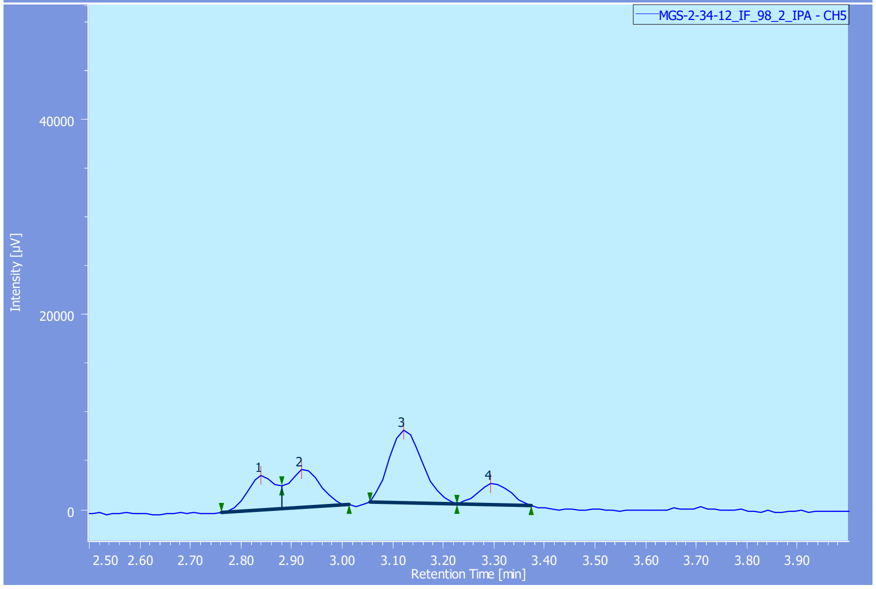

j) **Ethyl (1*R*,2*S*)-2-acetoxy-2-(*p*-tolyl)cyclopropane-1-carboxylate (5m)** GC analysis for diastereomeric and enantiomeric determination of compound **5m** using GC Separation Method 2. Racemic (*top*) and enzymatically (*bottom*) generated products are shown below.

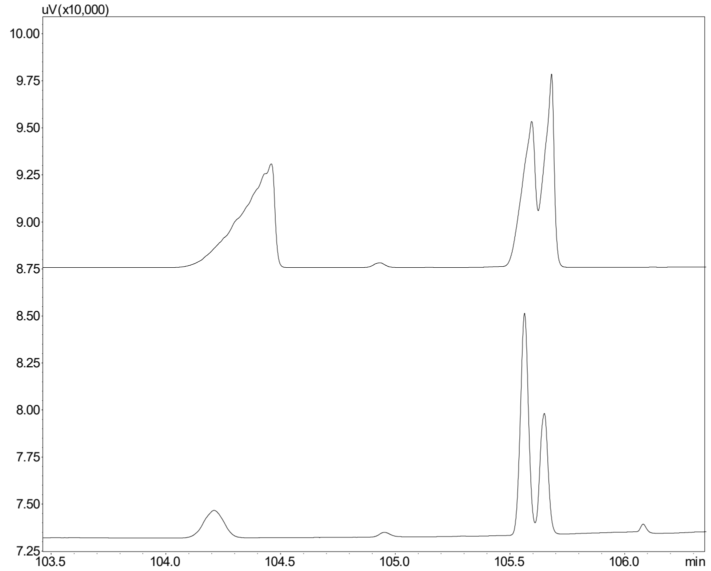

k) **Ethyl (1R,2S)-2-acetoxy-2-(4-fluorophenyl)cyclopropane-1-carboxylate** (**5n**). GC analysis for diastereomeric and enantiomeric determination of compound **5n** using GC Separation Method 1. Racemic (top) and enzymatically (bottom) generated products are shown below.

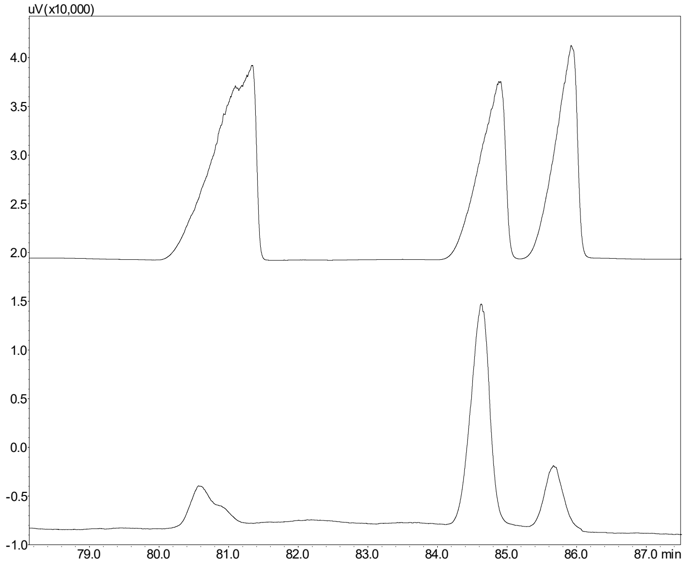

l) **Ethyl (1R,2S)-2-acetoxy-2-(4-bromophenyl) cyclopropane-1-carboxylate (5o).** SFC analysis for diastereomeric and enantiomeric determination of compound **5o**. Racemic (*top*) and enzymatically (*bottom*) generated products are shown below. Compound **5o** was analyzed using Daicel Chiralpak IA column and 5% of iPrOH

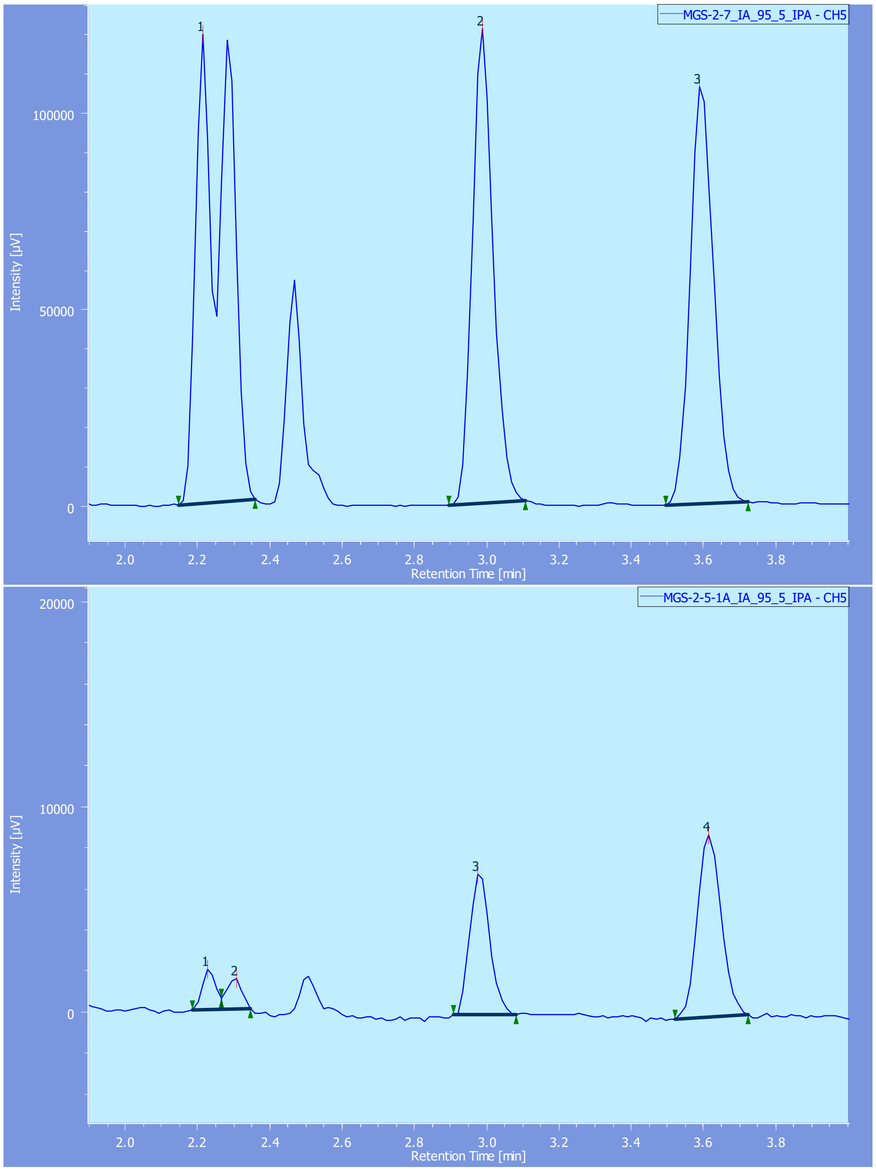

m) **Ethyl (1*R*,2*S*)-2-acetoxy-2-(4-(trifluoromethyl)phenyl)cyclopropane-1-carboxylate (5p).** GC analysis for diastereomeric and enantiomeric determination of compound **5p** using GC Separation Method 2. Racemic (*top*) and enzymatically (*bottom*) generated products are shown below.

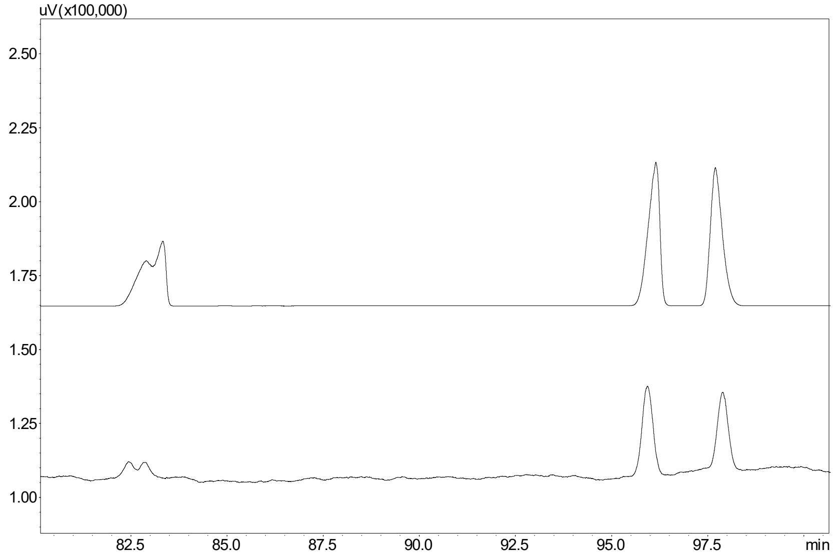

n) **Ethyl (1R,2S)-2-acetoxy-2-(4-methoxyphenyl) cyclopropane-1-carboxylate (5q).** SFC analysis for diastereomeric and enantiomeric determination of compound **5q**. Racemic (*top*) and enzymatically (*bottom*) generated products are shown below. Compound **5q** was analyzed using Daicel Chiralpak IA column and 5% of iPrOH

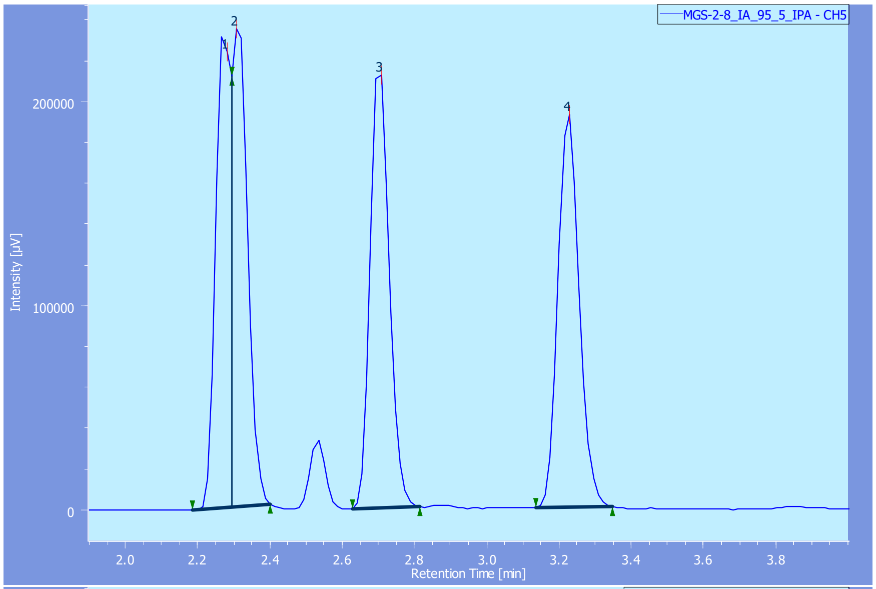

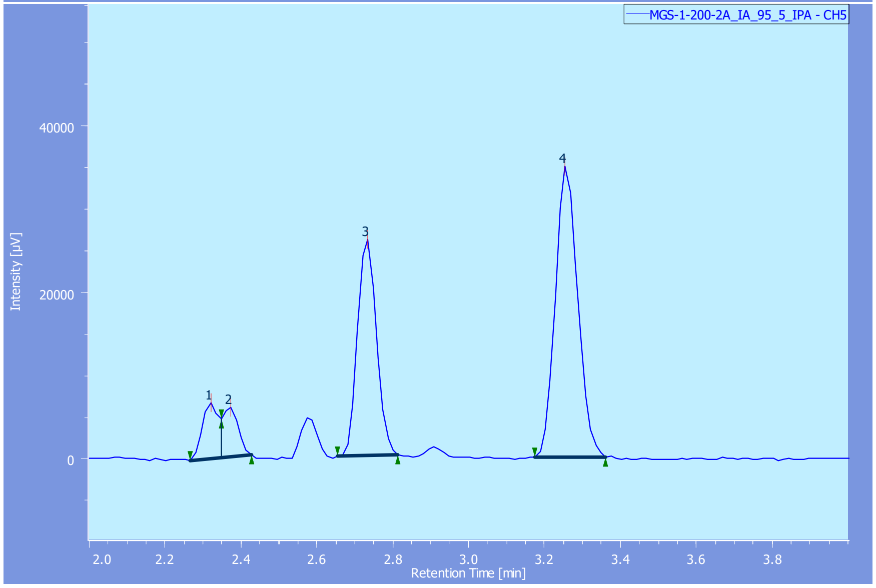

o) **(1*S*,2*R*)-2-cyanocyclopropyl benzoate (7a).** GC analysis for diastereomeric and enantiomeric determination of compound **5p** using GC Separation Method 1. WT DHP (*top*) and (DHP H55A, V59A) (*bottom*) generated products are shown below.

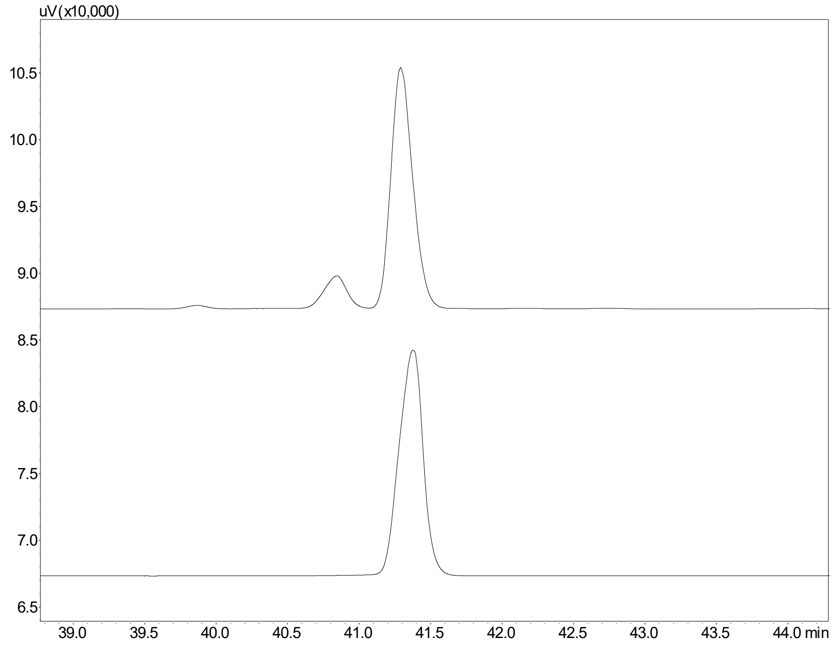

p) **Ethyl phenylalaninate (8b).** GC analysis for diastereomeric and enantiomeric determination of compound **8b**. GC Separation Method 3. Racemic (*top*) and enzymatically (*bottom*) generated products are shown below.

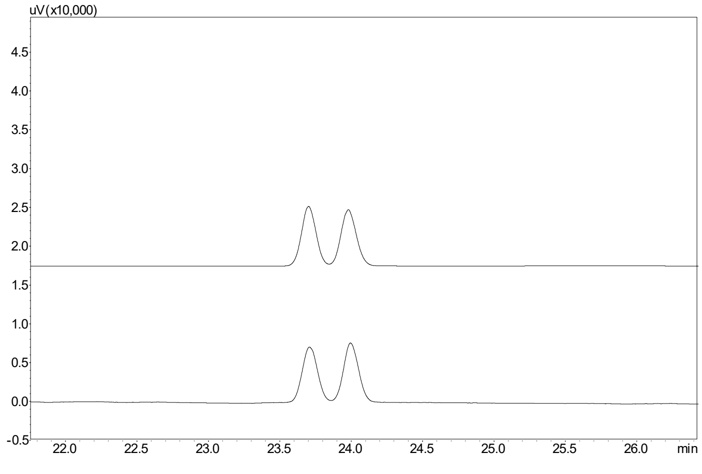

q) **Ethyl (*S*)-2-(phenylthio)pent-4-enoate (10a).** GC analysis for diastereomeric and enantiomeric determination of compound **5p** GC Separation Method 3. Racemic (*top*) and enzymatically (*bottom*) generated products are shown below.

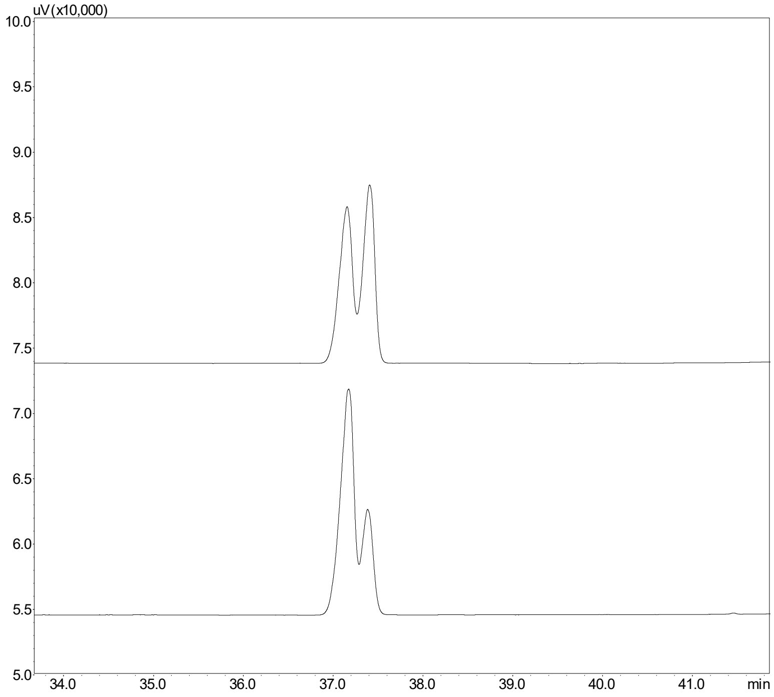

Scheme 1. Mechanistic proposal for the radical stepwise vs concerted carbene transfer pathway in DHP-catalyzed cyclopropanation of β-cis-deutero-styrene with EDA.

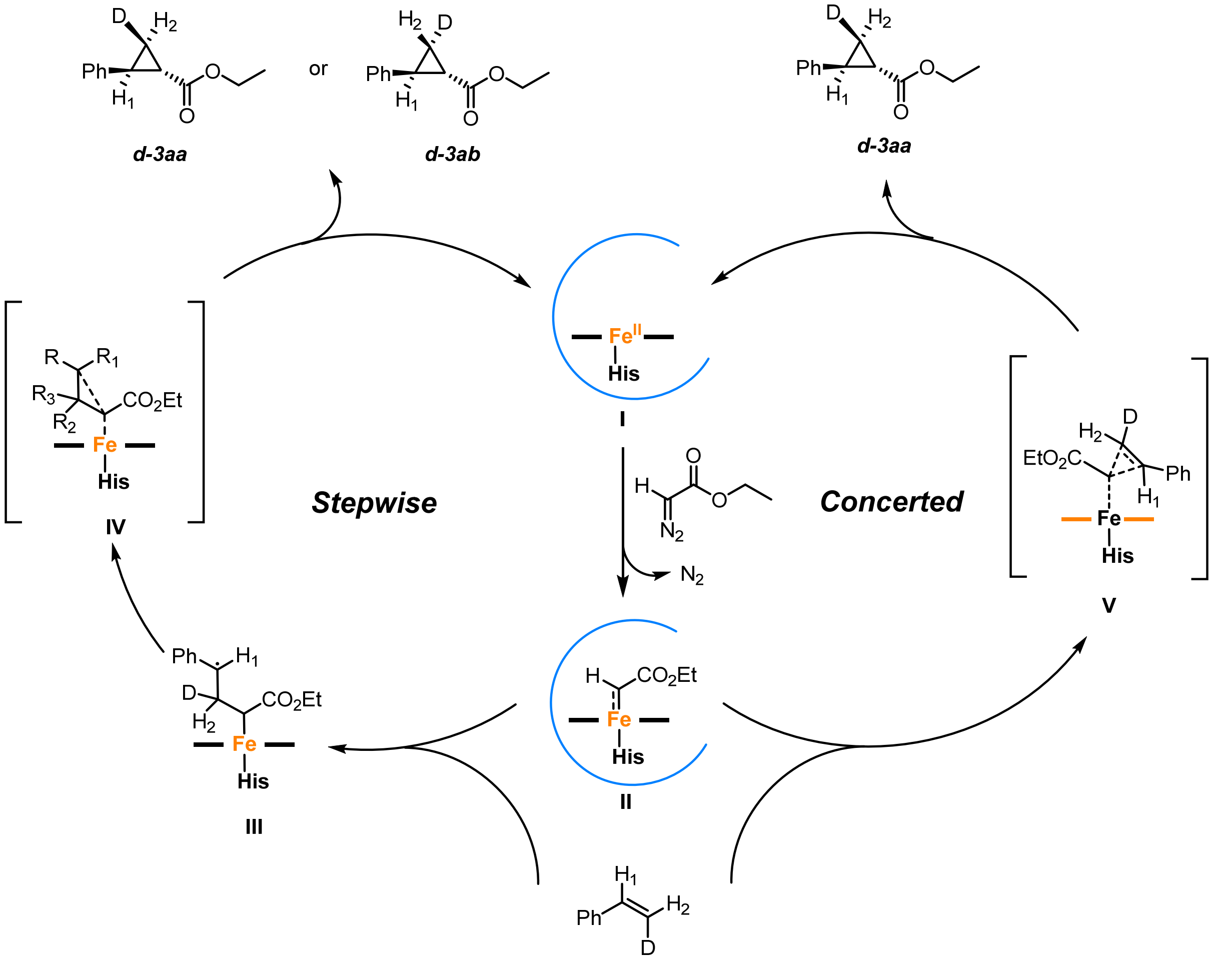

Compound Characterization

Vinyl 4-methylbenzoate (4b)

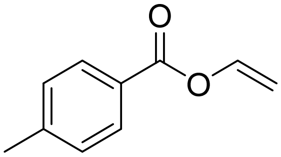

Following the general procedure A, **4b** was isolated as a colorless oil. (211 mg, 62% yield).

^{1}H NMR (500 MHz, $CDCl_3$) δ 7.99 (d, J = 8.3 Hz, 2H), 7.51 (dd, J = 13.9, 6.2 Hz, 1H), 7.26 (d, J = 7.6 Hz, 2H), 5.05 (dd, J = 13.9, 1.6 Hz, 1H), 4.68 (dd, J = 6.3, 1.6 Hz, 1H), 2.42 (s, 3H) ppm.

Vinyl 4-methoxybenzoate (4c)

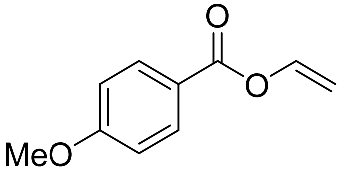

Following the general procedure A, **4c** was isolated as a white solid. (271 mg, 73% yield).

^{1}H NMR (500 MHz, $CDCl_3$**)** δ 8.06 (d, *J* = 9.1 Hz, 2H), 7.50 (ddd, *J* = 14.0, 6.3, 1.2 Hz, 1H), 6.95 (d, *J* = 9.0 Hz, 2H), 5.03 (dd, *J* = 14.0, 1.6 Hz, 1H), 4.67 (dd, *J* = 6.3, 1.6 Hz, 1H), 3.88 (s, 3H) ppm.

Hex-1-en-2-yl benzoate (4d)

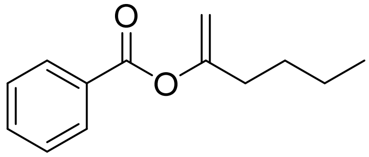

Following the general procedure B, **4d** was isolated as a colorless oil. (582 mg, 67% yield).

^{1}H NMR (500 MHz, $CDCl_3$) δ 8.12 – 8.06 (m, 2H), 7.62 – 7.56 (m, 1H), 7.47 (t, *J* = 7.9 Hz, 2H), 4.87 – 4.81 (m, 2H), 2.34 (t, *J* = 7.6 Hz, 2H), 1.58 – 1.46 (m, 2H), 1.44 – 1.34 (m, 2H), 0.92 (t, *J* = 7.4 Hz, 3H).

3-Methylbut-1-en-2-yl Benzoate (4e)

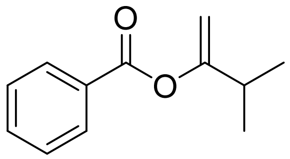

Following the general procedure B, **4e** was isolated as a colorless oil. (406 mg, 43% yield).

^{1}H NMR (500 MHz, $CDCl_3$) δ 8.10 (dd, *J* = 8.2, 1.5 Hz, 2H), 7.62 – 7.56 (m, 1H), 7.47 (t, *J* = 7.8 Hz, 2H), 4.88 (dd, *J* = 1.9, 1.0 Hz, 1H), 4.84 (d, *J* = 1.8 Hz, 1H), 2.64 – 2.51 (m, 1H), 1.16 (d, *J* = 6.9 Hz, 6H). ***^{13}C{1H}* NMR** (126 MHz, $CDCl_3$) δ 165.6, 162.2, 133.9, 130.7, 130.7, 129.2, 100.2, 33.2, 20.9 ppm. **HRMS** (ESI) *m/z* calculated for $C_{12}H_{15}O_2^+$ $[M+H]^+$ 191.1072, found 191.1064.

1-Cyclopropylvinyl Benzoate (4f)

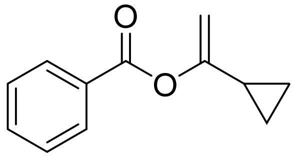

Following the general procedure B, **4f** was isolated as a colorless oil. (518 mg, 55% yield).

^{1}H NMR (500 MHz, $CDCl_3$) δ 8.11 – 8.04 (m, 2H), 7.63 – 7.56 (m, 1H), 7.51 – 7.42 (m, 2H), 4.90 (dd, *J* = 1.8, 0.7 Hz, 1H), 4.82 (d, *J* = 1.7 Hz, 1H), 1.71 – 1.61 (m, 1H), 0.78 – 0.69 (m, 2H), 0.72 – 0.65 (m, 2H). ***^{13}C{1H}* NMR** (126 MHz, $CDCl_3$) δ 165.5, 157.5, 134.1, 130.7, 130.5, 129.2, 100.7, 14.7, 6.4 ppm.

1-Cyclopentylvinyl Benzoate (4g)

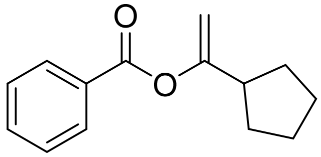

Following the general procedure B, **4g** was isolated as a colorless oil. (16 mg, 3% yield).

^{1}H NMR (500 MHz, $CDCl_3$) δ 8.13 – 8.04 (m, 2H), 7.62 – 7.55 (m, 1H), 7.47 (t, *J* = 7.8 Hz, 2H), 4.90 (d, *J* = 1.4 Hz, 1H), 4.84 (d, *J* = 1.7 Hz, 1H), 2.82 – 2.72 (m, 1H), 1.95 – 1.83 (m, 2H), 1.70 (dttd, *J* = 11.8, 8.7, 6.0, 3.5 Hz, 2H), 1.63 – 1.52 (m, 4H). ***^{13}C{1H}* NMR** (126 MHz, $CDCl_3$) δ 165.6, 159.9, 133.9, 130.8, 130.7, 129.2, 100.7, 44.6, 31.3, 25.7 ppm. **HRMS** (ESI) *m/z* calculated for $C_{14}H_{17}O_2^+$ $[M+H]^+$ 217.1228, found 217.1220.

1-Cyclohexylvinyl Benzoate (4h)

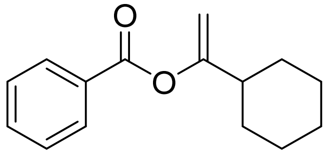

Following the general procedure B, **4h** was isolated as a colorless oil. (254 mg, 25% yield).
^{1}**H NMR** (500 MHz, $CDCl_3$) δ 8.13 – 8.06 (m, 2H), 7.63 – 7.55 (m, 1H), 7.47 (dd, *J* = 8.7, 6.9 Hz, 2H), 4.84 (d, *J* = 1.4 Hz, 1H), 4.82 (d, *J* = 1.8 Hz, 1H), 2.24 (tt, *J* = 10.6, 3.4 Hz, 1H), 2.03 – 1.92 (m, 2H), 1.82 – 1.71 (m, 2H), 1.71 – 1.63 (m, 1H), 1.26 (ddt, *J* = 9.7, 7.8, 4.9 Hz, 4H).
^{13}**C{1H} NMR** (126 MHz, $CDCl_3$) δ 165.6, 161.5, 133.9, 130.7, 130.7, 129.2, 100.4, 42.6, 31.4, 26.8, 26.7 ppm.

1-Phenylvinyl Benzoate (4i)

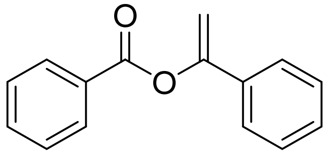

Following the general procedure B, **4i** was isolated as a colorless oil.
^{1}**H NMR** (500 MHz, $CDCl_3$) δ 8.24 – 8.17 (m, 2H), 7.64 (ddt, *J* = 8.8, 7.0, 1.3 Hz, 1H), 7.57 – 7.48 (m, 4H), 7.39 – 7.30 (m, 3H), 5.60 (d, *J* = 2.2 Hz, 1H), 5.17 (d, *J* = 2.3 Hz, 1H). ^{13}**C{1H} NMR** (126 MHz, $CDCl_3$) δ 165.6, 153.9, 135.0, 134.3, 130.9, 130.2, 129.7, 129.4, 129.3, 125.7, 103.1, 77.7, 30.5 ppm.

1-phenylvinyl acetate (4j)

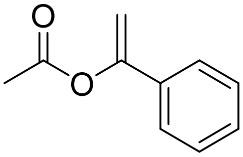

Following the general procedure C, **4j** was isolated as a light yellow oil. (79 mg, 6% yield).
^{1}**H NMR** (500 MHz, $CDCl_3$) δ 7.49 – 7.44 (m, 2H), 7.38 – 7.30 (m, 3H), 5.48 (d, *J* = 2.2 Hz, 1H), 5.03 (d, *J* = 2.2 Hz, 1H), 2.28 (s, 3H).

1-(*o*-tolyl)vinyl acetate (4k)

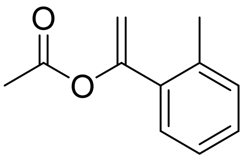

Following the general procedure C, **4k** was isolated as a light yellow oil.

^{1}H NMR (400 MHz, $CDCl_3$) δ 7.37 (d, *J* = 7.2 Hz, 1H), 7.28 – 7.19 (m, 2H), 7.18 (d, *J* = 6.9 Hz, 2H), 5.18 (d, *J* = 1.6 Hz, 1H), 5.02 (d, *J* = 1.5 Hz, 1H), 2.41 (s, 3H), 2.14 (s, 3H).

1-(*m*-tolyl)vinyl acetate (4l)

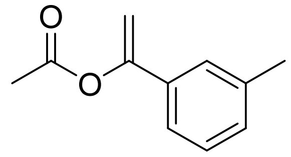

Following the general procedure C, **4l** was isolated as a light yellow oil.
^{1}H NMR (500 MHz, $CDCl_3$) δ 7.31 – 7.18 (m, 3H), 7.14 (d, *J* = 7.1 Hz, 1H), 5.45 (s, 1H), 5.00 (s, 1H), 2.35 (s, 3H), 2.28 (s, 3H).

1-(*p*-tolyl)vinyl acetate (4m)

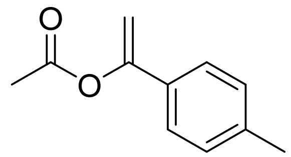

Following the general procedure C, **4m** was isolated as a light yellow oil.
^{1}H NMR (500 MHz, $CDCl_3$) δ 7.35 (d, *J* = 6.3 Hz, 1H), 7.15 (d, *J* = 7.3 Hz, 2H), 5.42 (d, *J* = 1.9 Hz, 1H), 4.96 (d, *J* = 2.0 Hz, 1H), 2.34 (s, 2=3H), 2.27 (s, 3H).

1-(4-fluorophenyl)vinyl acetate (4n)

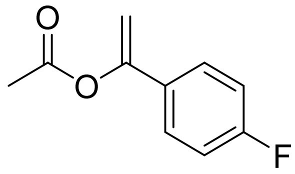

Following the general procedure C, **4n** was isolated as a light yellow oil. (216 mg, 33% yield).
^{1}H NMR (500 MHz, $CDCl_3$) δ 7.47 – 7.40 (m, 2H), 7.03 (t, *J* = 8.7 Hz, 2H), 5.40 (d, *J* = 2.3 Hz, 1H), 5.01 (d, *J* = 2.3 Hz, 1H), 2.27 (s, 3H).

1-(4-bromophenyl)vinyl acetate (4o)

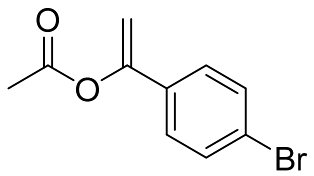

Following the general procedure C, **4o** was isolated as a light yellow oil. (177 mg, 25% yield).

^{1}H NMR (500 MHz, $CDCl_3$) δ 7.47 (dd, *J* = 8.8, 1.9 Hz, 2H), 7.36 – 7.29 (m, 2H), 5.46 (d, *J* = 2.4 Hz, 1H), 5.05 (d, *J* = 2.2 Hz, 1H), 2.27 (s, 3H).

1-(4-(trifluoromethyl)phenyl)vinyl acetate (4p)

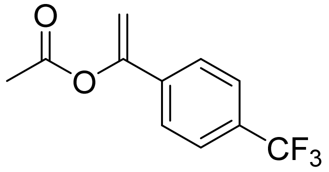

Following the general procedure C, **4p** was isolated as a light yellow oil.

^{1}H NMR (500 MHz, $CDCl_3$) δ 7.63 – 7.59 (m, 2H), 7.59 – 7.55 (m, 2H), 5.57 (d, *J* = 2.4 Hz, 1H), 5.16 (d, *J* = 2.5 Hz, 1H), 2.29 (s, 3H).

1-(4-methoxyphenyl)vinyl acetate (4q)

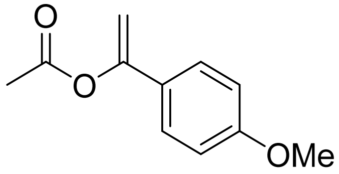

Following the general procedure C, **4q** was isolated as a white solid. (197 mg, 31% yield).

^{1}H NMR (500 MHz, $CDCl_3$) δ 7.40 – 7.35 (m, 2H), 6.85 – 6.80 (m, 2H), 5.33 (d, *J* = 2.2 Hz, 1H), 4.89 (d, *J* = 2.2 Hz, 1H), 3.73 (s, 3H), 2.22 (s, 3H).

(1S,2S)-2-(ethoxycarbonyl)cyclopropyl benzoate (5a)

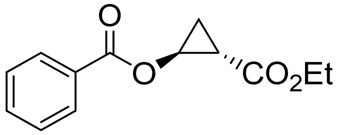

Following the general procedure D, *trans* isomer of **5a** was isolated as colorless oil. (11 mg, 47% yield)

^{1}H NMR (500 MHz, $CDCl_3$) δ 7.99 (dd, *J* = 9.6, 1.3 Hz, 2H), 7.60 – 7.55 (m, 1H), 7.44 (dd, *J* = 15.7, 1.6 Hz, 2H), 4.66 (ddd, *J* = 6.8, 4.2, 2.3 Hz, 1H), 4.18 (q, *J* = 7.1 Hz, 2H), 2.02 (ddd, *J* = 9.9, 6.3, 2.3 Hz, 1H), 1.54 (q, *J* = 6.4 Hz, 1H), 1.43 (dq, *J* = 6.1, 4.3 Hz, 2H), 1.29 (t, *J* = 7.1 Hz, 3H).

(1S,2S)-2-(ethoxycarbonyl)cyclopropyl 4-methylbenzoate (5b)

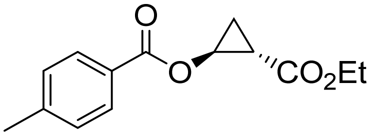

Following the general procedure D, *trans* isomer of **5b** was isolated as colorless oil. (10 mg, 13% yield).

¹H NMR (500 MHz, CDCl₃) δ 7.89 (dd, *J* = 8.3, 1.8 Hz, 2H), 7.22 (d, *J* = 8.1 Hz, 2H), 4.50 (td, *J* = 6.9, 4.7 Hz, 1H), 4.15 – 4.08 (m, 2H), 2.40 (s, 3H), 2.05 (dt, *J* = 8.9, 7.1 Hz, 1H), 1.73 (td, *J* = 6.8, 4.6 Hz, 1H), 1.35 (dt, *J* = 8.9, 6.6 Hz, 1H), 1.19 (t, *J* = 7.1 Hz, 3H). ***^{13}C{1H}* NMR** (126 MHz, $CDCl_3$) δ 144.7, 130.5, 129.8, 61.5, 53.5, 30.4, 22.4, 20.8, 14.9, 12.9 ppm. **HRMS** (ESI) *m/z* calculated for $C_{14}H_{17}O_4^+$ $[M+H]^+$ 249.1127, found 249.1117.

(1*S*,2*S*)-2-(ethoxycarbonyl)cyclopropyl 4-methoxybenzoate (5c)

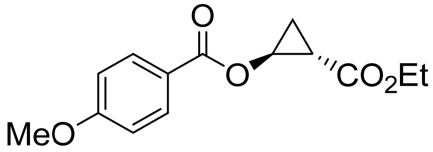

Following the general procedure D, *trans* isomer of **5c** was isolated as colorless oil (5 mg, 7% yield).

^{1}H NMR (500 MHz, $CDCl_3$) δ 7.95 – 7.92 (m, 2H), 6.92 – 6.90 (m, 2H), 4.63 – 4.60 (ddd, J = 6.7, 4.2, 2.3 Hz, 1H), 4.18 – 4.15 (q, J = 7.2 Hz, 2H), 3.86 (d, J = 1.6 Hz, 3H), 1.99 (ddd, J = 9.8, 6.3, 2.3 Hz, 1H), 1.54 – 1.49 (m, 1H), 1.41 (ddd, J = 10.1, 6.2, 4.3 Hz, 1H), 1.29 (t, J = 7.2 Hz, 3H). ***^{13}C{1H}* NMR** (126 MHz, $CDCl_3$) δ 172.4, 164.4, 132.4, 122.4, 114.4, 61.6, 56.2, 55.3, 30.4, 21.5, 15.1, 14.9 ppm. **HRMS** (ESI) *m/z* calculated for $C_{14}H_{17}O_5^+$ $[M+H]^+$ 265.1076, found 265.1066.

(1*S*,2*R*)-1-butyl-2-(ethoxycarbonyl)cyclopropyl benzoate (5d)

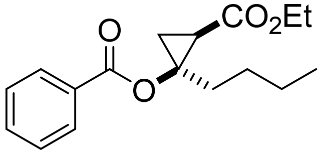

Following the general procedure D, *trans* isomer of **5d** was isolated as colorless oil. (18 mg, 13% yield). The isolated compound contains a minor amount (<5%) of a second diastereomer.

^{1}H NMR (500 MHz, $CDCl_3$) δ 8.02 – 7.93 (m, 2H), 7.58 – 7.50 (m, 1H), 7.46 – 7.37 (m, 2H), 4.19 (q, *J* = 7.1 Hz, 2H), 2.16 – 2.05 (m, 2H), 1.99 (ddd, *J* = 15.2, 10.5, 4.8 Hz, 1H), 1.51 – 1.42 (m, 3H), 1.39 – 1.25 (m, 5H), 0.84 (t, *J* = 7.2 Hz, 3H). ***^{13}C{1H}* NMR** (126 MHz, $CDCl_3$) δ 171.8, 166.3, 133.9, 130.8, 130.3, 129.1, 66.6, 61.6, 29.5, 28.9, 26.9, 23.1, 20.5, 15.0, 14.8 ppm. **HRMS** (ESI) *m/z* calculated for $C_{17}H_{23}O_4^+$ $[M+H]^+$ 291.1596, found 291.1584.

(1R,2R)-2-(ethoxycarbonyl)-1-isopropylcyclopropyl benzoate (5e)

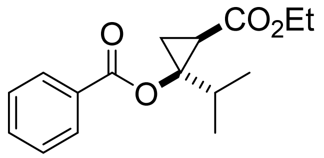

Following the general procedure D, *trans* isomer of **5e** was isolated as colorless oil. (5 mg, 14% yield).

^{1}H NMR (500 MHz, $CDCl_3$) δ 8.00 – 7.95 (m, 2H), 7.56 – 7.51 (m, 1H), 7.44 – 7.38 (m, 2H), 4.08 (qq, *J* = 10.8, 7.1 Hz, 2H), 2.21 (hept, *J* = 6.6 Hz, 1H), 2.03 (dd, *J* = 9.1, 7.1 Hz, 1H), 1.79 (t, *J* = 6.9 Hz, 1H), 1.39 (dd, *J* = 9.1, 6.7 Hz, 1H), 1.16 (t, *J* = 7.1 Hz, 3H), 1.01 (dd, *J* = 15.2, 6.8 Hz, 6H). ***^{13}*C*{1H}* NMR** (126 MHz, $CDCl_3$) δ 170.7, 166.8, 133.7, 131.0, 130.4, 129.0, 77.9, 69.2, 61.5, 33.3, 24.7, 19.5, 19.3, 17.6, 14.9. **HRMS** (ESI) *m/z* calculated for $C_{16}H_{21}O_4^+$ $[M+H]^+$ 277.1435, found 277.1429.

(1R,2R)-2-(ethoxycarbonyl)-[1,1'-bi(cyclopropan)]-1-yl benzoate (5f)

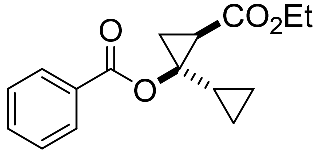

Following the general procedure D, *trans* isomer of **5f** was isolated as colorless oil. (8 mg, 20% yield). The isolated compound contains a minor amount (<5%) of a second diastereomer.

^{1}H NMR (400 MHz, $CDCl_3$) δ 8.00 (dt, *J* = 7.0, 1.4 Hz, 2H), 7.60 – 7.53 (m, 1H), 7.44 (t, *J* = 7.8 Hz, 2H), 4.23 (qd, *J* = 7.1, 1.5 Hz, 2H), 2.20 (dd, *J* = 9.8, 7.2 Hz, 1H), 1.65 (tt, *J* = 8.4, 6.0 Hz, 1H), 1.45 (t, *J* = 6.9 Hz, 1H), 1.38 (dd, *J* = 9.8, 6.5 Hz, 1H), 1.32 (t, *J* = 7.1 Hz, 3H), 0.67 (dddd, *J* = 10.0, 8.2, 5.5, 3.1 Hz, 1H), 0.62 – 0.49 (m, 3H). ***^{13}*C*{1H}* NMR** (126 MHz, $CDCl_3$) δ 170.4, 167.0, 133.7, 130.9, 130.4, 129.0, 66.6, 61.5, 26.5, 17.9, 16.8, 14.9, 4.4, 4.3 ppm. **HRMS** (ESI) *m/z* calculated for $C_{16}H_{19}O_4^+$ $[M+H]^+$ 275.1283, found 275.1274.

(1R,2R)-1-cyclopentyl-2-(ethoxycarbonyl)cyclopropyl benzoate (5g)

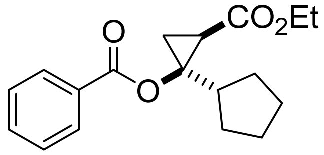

Following the general procedure using styrene, *trans* isomer of **5g** was isolated as colorless oil.

^{1}H NMR (500 MHz, CDCl3) δ 7.98 – 7.94 (m, 2H), 7.56 – 7.50 (m, 1H), 7.41 (t, J = 7.6 Hz, 2H), 4.08 (qq, J = 10.8, 7.1 Hz, 2H), 2.74 – 2.63 (m, 1H), 2.05 (dd, J = 9.1, 7.1 Hz, 1H), 1.78 (dt, J = 13.6, 7.7 Hz, 3H), 1.63 (dh, J = 13.3, 3.4 Hz, 2H), 1.55 (qt, J = 7.6, 2.8 Hz, 2H), 1.38 (dd, J = 9.1, 6.6 Hz, 1H), 1.17 (t, J = 7.1 Hz, 4H). **^{13}C*{1H}* NMR** (126 MHz, $CDCl_3$) ^{13}C NMR (126

MHz, $CDCl_3$) δ 170.7, 166.8, 133.6, 131.1, 130.3, 129.0, 67.6, 61.5, 43.9, 30.0, 29.6, 25.7, 25.1, 17.3, 14.9. **HRMS** (ESI) *m/z* calculated for $C_{18}H_{23}O_4^+$ $[M+H]^+$ 303.1596, found 303.1586.

(1R,2R)-1-cyclohexyl-2-(ethoxycarbonyl)cyclopropyl benzoate (5h)

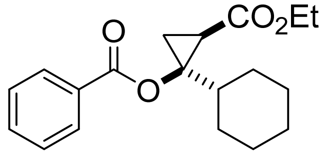

Following the general procedure D, *trans* isomer of **5h** was isolated as colorless oil. (5 mg, 4% yield).

^{1}H NMR (500 MHz, $CDCl_3$) δ 7.97 (d, *J* = 7.7 Hz, 2H), 7.54 (t, *J* = 7.5 Hz, 1H), 7.46 – 7.35 (m, 2H), 4.07 (tddd, *J* = 16.2, 12.1, 7.6, 4.2 Hz, 2H), 2.08 – 1.99 (m, 1H), 1.94 – 1.80 (m, 3H), 1.76 (dq, *J* = 10.6, 3.2 Hz, 3H), 1.65 (d, *J* = 13.1 Hz, 1H), 1.39 (ddd, *J* = 9.1, 6.6, 2.4 Hz, 1H), 1.28 – 1.19 (m, 2H), 1.16 (td, *J* = 7.2, 2.3 Hz, 3H), 1.12 – 0.95 (m, 2H). ***^{13}C{1H}* NMR** (126 MHz, $CDCl_3$) δ 170.7, 166.8, 133.6, 131.0, 130.4, 129.0, 68.8, 61.5, 54.2, 42.9, 29.9, 29.7, 26.8, 26.8, 26.7, 24.5, 17.6, 14.9 ppm. **HRMS** (ESI) *m/z* calculated for $C_{19}H_{25}O_4^+$ $[M+H]^+$ 317.1753, found 317.1743.

2-(ethoxycarbonyl)-1-phenylcyclopropyl benzoate (5i)

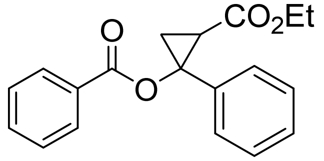

Following the general procedure D, **5i** was isolated as a *mixture of cis and trans isomers*, yellow oil (54 mg, 40% yield).

We were unable to separate the diastereomers, resulting in a mixture of cis and trans with ratios of 1.4:1(*trans:cis*). **^{1}H NMR (500 MHz, $CDCl_3$)** δ 8.00 (ddd, *J* = 13.0, 8.4, 1.4 Hz, 3.59H), 7.63 – 7.58 (m, 1.53H), 7.55 (dddd, *J* = 10.9, 5.3, 2.7, 1.4 Hz, 1.65H), 7.46 – 7.37 (m, 5.57H), 7.36 – 7.31 (m, 2.83H), 7.31 – 7.27 (m, 1.38H), 4.17 (dddd, *J* = 18.0, 10.8, 7.2, 3.7 Hz, 2H), 3.96 – 3.90 (m, 1.48H), 2.57 (dd, *J* = 9.6, 7.4 Hz, 0.76H), 2.48 (dd, *J* = 9.2, 7.5 Hz, 1.01H), 2.32 (t, *J* = 7.2 Hz, 0.84H), 2.18 (dd, *J* = 7.6, 6.5 Hz, 1.03H), 1.83 (dd, *J* = 9.2, 6.6 Hz, 1.08H), 1.76 (dd, *J* = 9.6, 6.9 Hz, 0.81H), 1.23 (t, *J* = 7.1 Hz, 3.12H), 1.00 (t, *J* = 7.1 Hz, 2.41H). **HRMS** (ESI) *m/z* calculated for $C_{19}H_{19}O_4^+$ $[M+H]^+$ 311.1283, found 311.1271.

Ethyl (1R,2S)-2-acetoxy-2-phenylcyclopropane-1-carboxylate (5j)

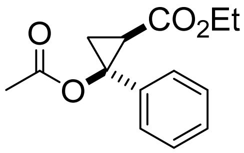

Following the general procedure D, *trans* isomer of **5j** was isolated as colorless oil. (36 mg, 24% yield). The isolated compound contains a minor amount (<5%) of a second diastereomer.

^{1}H NMR (500 MHz, CDCl$_3$) δ 7.40 – 7.35 (m, 2H), 7.33 (ddd, *J* = 7.7, 6.7, 1.3 Hz, 2H), 7.30 – 7.25 (m, 1H), 4.19 (qd, *J* = 7.2, 0.9 Hz, 2H), 2.40 (dd, *J* = 9.2, 7.4 Hz, 1H), 2.04 (s, 3H), 2.01 (dd, *J* = 7.5, 6.4 Hz, 1H), 1.66 (dd, *J* = 9.3, 6.5 Hz, 1H), 1.29 (t, *J* = 7.2 Hz, 3H). ***^{13}*C*{1H}* NMR** (126 MHz, CDCl$_3$) δ 171.0, 170.1, 139.1, 129.2, 128.8, 127.6, 64.6, 61.7, 28.6, 21.7, 21.2, 14.9 ppm. **HRMS** (ESI) *m/z* calculated for $C_{14}H_{17}O_4^+$ $[M+H]^+$ 249.1127, found 249.1117.

Ethyl 2-acetoxy-2-(*o*-tolyl)cyclopropane-1-carboxylate (5k)

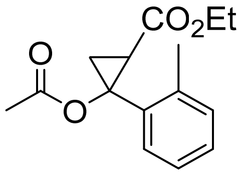

Following the general procedure D, a *mixture of cis and trans isomers* as colorless oil. (57 mg, 27% yield).

We were unable to separate the diastereomers, resulting in a mixture of cis and trans with ratios of 1.81:1(*trans:cis)* **^{1}H NMR (400 MHz, CDCl$_3$)** δ 7.61 (d, *J* = 6.5 Hz, 0.51H), 7.54 (dd, *J* = 7.5, 1.7 Hz, 0.51H), 7.26 – 7.06 (m, 3.34H), 4.30 – 4.16 (m, 2H), 3.96 – 3.83 (m, 1.13H), 2.51 (s, 1.59H), 2.44 (s, 2.12H), 2.33 (dd, *J* = 9.3, 7.3 Hz, 0.57H), 2.19 – 2.12 (m, 0.66H), 1.99 (t, *J* = 6.8 Hz, 0.70H), 1.94 (s, 0.39H), 1.64 (dd, *J* = 9.6, 6.8 Hz, 0.51H), 1.61 – 1.55 (m, 0.57H), 1.32 (t, *J* = 8.1 Hz, 3.77H), 0.97 (t, *J* = 7.2 Hz, 1.69H). **HRMS** (ESI) *m/z* calculated for $C_{15}H_{18}NaO_4^+$ $[M+Na]^+$ 285.1105.1127, found 285.1091.

Ethyl (1R,2S)-2-acetoxy-2-(m-tolyl)cyclopropane-1-carboxylate (5l)

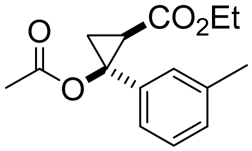

Following the general procedure D, *trans* isomer of **5l** was isolated as colorless oil. (5 mg, 2% yield).

^{1}H NMR (400 MHz, CDCl$_3$) δ 7.25 – 7.20 (m, 1H), 7.20 – 7.16 (m, 2H), 7.09 (d, *J* = 7.2 Hz, 1H), 4.19 (qt, *J* = 7.1, 1.4 Hz, 2H), 2.44 – 2.34 (m, 2H), 2.34 (s, 3H), 2.04 (s, 3H), 1.99 (ddd, *J* = 7.5, 6.4, 1.1 Hz, 1H), 1.65 (ddd, *J* = 9.3, 6.4, 1.1 Hz, 1H), 1.30 (t, *J* = 6.6 Hz, 3H). ***^{13}*C*{1H}* NMR** (126 MHz, CDCl$_3$)δ 171.1, 170.2, 139.0, 138.9, 129.5, 129.1, 128.2, 124.7, 64.6, 61.7,

28.5, 22.2, 21.7, 21.2, 14.9 ppm. **HRMS** (ESI) *m/z* calculated for $C_{15}H_{18}NaO_4^+$ $[M+Na]^+$ 285.1105.1127, found 285.1093.

Ethyl (1R,2S)-2-acetoxy-2-(p-tolyl)cyclopropane-1-carboxylate (5m)

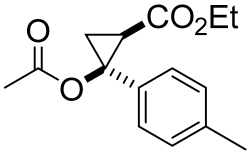

Following the general procedure D, *trans* isomer of **5m** was isolated as colorless oil. (9 mg, 4% yield).

^{1}H NMR (400 MHz, $CDCl_3$) δ 7.39 (d, *J* = 8.0 Hz, 2H), 7.11 (d, *J* = 7.8 Hz, 2H), 3.92 (q, *J* = 7.1 Hz, 2H), 2.38 (dd, *J* = 9.5, 7.3 Hz, 1H), 2.31 (s, 3H), 2.14 (t, *J* = 7.1 Hz, 1H), 1.95 (s, 2H), 1.60 (dd, *J* = 9.5, 6.7 Hz, 1H), 1.02 (t, *J* = 7.1 Hz, 3H). ***^{13}C{1H}* NMR** (126 MHz, $CDCl_3$) δ 171.1, 170.2, 138.7, 136.2, 130.5, 129.8, 127.9, 64.6, 61.9, 61.7, 28.3, 21.8, 21.7, 20.9, 14.9, 14.8 ppm. **HRMS** (ESI) *m/z* calculated for $C_{15}H_{18}NaO_4^+$ $[M+Na]^+$ 285.1105.1127, found 285.1090.

Ethyl (1R,2S)-2-acetoxy-2-(4-fluorophenyl)cyclopropane-1-carboxylate (5n)

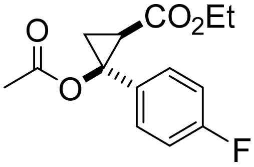

Following the general procedure D, *trans* isomer of **5n** was isolated as colorless oil. (7 mg, 10% yield).

^{1}H NMR (400 MHz, $CDCl_3$) δ 7.47 – 7.38 (m, 2H), 7.07 – 6.97 (m, 2H), 4.24 – 4.14 (m, 2H), 2.35 (ddd, *J* = 9.3, 7.2, 4.9 Hz, 1H), 2.01 (s, 3H), 2.00 – 1.95 (m, 1H), 1.66 – 1.55 (m, 1H), 1.30 (t, *J* = 6.1 Hz, 3H). ***^{13}C{1H}* NMR** (126 MHz, $CDCl_3$) δ 171.1, 170.0, 130.4 (d, *J* = 8.4 Hz), 116.1 (d, *J* = 21.7 Hz), 99.9, 64.2, 61.8, 28.1, 21.6, 20.8, 14.9 (quaternary C alpha to F and to cyclopropane not observed). **^{19}F NMR (376 MHz, $CDCl_3$)** δ -113.61 (q, *J* = 3.0 Hz). **HRMS** (ESI) *m/z* calculated for $C_{14}H_{16}FO_4^+$ $[M+H]^+$ 267.1032, found 267.1021.

NOE Enhancements

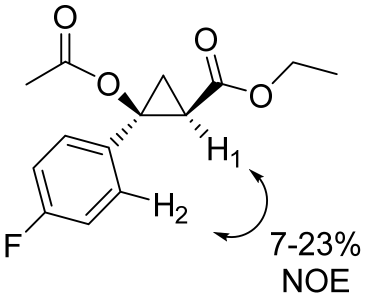

Major diastereomer

^{1}H NMR (400 MHz, CDCl$_3$) δ 7.03 (t, *J* = 8.5 Hz, 3H), 4.20 (q, *J* = 7.1 Hz, 2H), 2.47 – 2.24 (m, 1H), 2.01 (d, *J* = 9.0 Hz, 4H), 1.62 (dd, *J* = 9.3, 6.5 Hz, 1H), 1.31 (t, *J* = 7.1 Hz, 3H).

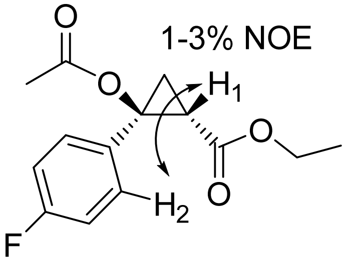

Minor diastereomer

^{1}H NMR (400 MHz, CDCl$_3$) δ 7.01 (t, *J* = 8.5 Hz, 2H), 3.94 (q, *J* = 7.1 Hz, 2H), 2.41 (t, *J* = 8.4 Hz, 1H), 2.14 (t, *J* = 7.1 Hz, 1H), 1.97 (s, 3H), 1.64 (dd, *J* = 9.6, 6.8 Hz, 1H), 1.04 (t, *J* = 7.1 Hz, 3H).

Ethyl (1R,2S)-2-acetoxy-2-(4-bromophenyl)cyclopropane-1-carboxylate (5o)

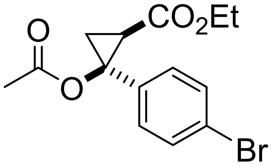

Following the general procedure D, *trans* isomer of **5o** was isolated as a yellow oil.

^{1}H NMR (400 MHz, CDCl$_3$) δ 7.46 (d, *J* = 8.6 Hz, 2H), 7.27 (d, *J* = 7.8 Hz, 2H), 4.18 (qd, *J* = 6.5, 3.4 Hz, 2H), 2.36 (dd, *J* = 9.3, 7.4 Hz, 1H), 2.07 – 1.95 (m, 4H), 1.61 (dd, *J* = 9.3, 6.5 Hz, 1H), 1.29 (t, *J* = 7.1 Hz, 3H). ***^{13}*C*{1H}* NMR** (126 MHz, CDCl$_3$) δ 171.0, 169.8, 138.2, 132.3, 129.6, 122.9, 64.1, 61.9, 28.4, 21.6, 20.9, 14.9. **HRMS** (ESI) *m/z* calculated for $C_{14}H_{16}BrO_4^+$ [M+H]$^+$ 327.0232, found 327.0219.

Ethyl (1R,2S)-2-acetoxy-2-(4-(trifluoromethyl)phenyl)cyclopropane-1-carboxylate (5p)

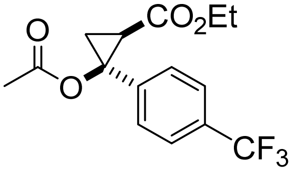

Following the general procedure D, *trans* isomer of **5p** was isolated as colorless oil. (6 mg, 9% yield).

^{1}H NMR (400 MHz, CDCl$_3$) δ 7.60 (d, *J* = 8.2 Hz, 2H), 7.46 (d, *J* = 8.1 Hz, 2H), 4.20 (q, *J* = 7.1 Hz, 2H), 2.48 – 2.39 (m, 1H), 2.11 – 2.03 (m, 4H), 1.68 (ddd, *J* = 9.3, 6.6, 1.0 Hz, 1H), 1.30

(t, J = 7.1 Hz, 3H). ^{13}**C{1H} NMR** (126 MHz, $CDCl_3$) δ 171.0, 169.6, 143.1, 130.9 (d, J = 32.7 Hz), 127.6, 126.2 (q, J = 3.7 Hz), 63.8, 61.9, 30.4, 28.9, 21.5, 21.4, 14.9 (quaternary C alpha to -CF3 not observed). **^{19}F {^{1}H} NMR (376 MHz, $CDCl_3$)** δ -62.87 ppm. **HRMS** (ESI) *m/z* calculated for $C_{15}H_{16}F_3O_4^+$ [M+H]$^+$ 317.0996, found 317.0987.

Ethyl (1R,2S)-2-acetoxy-2-(4-methoxyphenyl)cyclopropane-1-carboxylate (5q)

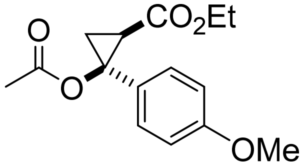

Following the general procedure D, *trans* isomer of **5q** was isolated as colorless oil. (9 mg, 6% yield).

^{1}H NMR (400 MHz, $CDCl_3$) δ 7.39 (d, J = 8.0 Hz, 2H), 6.86 (d, J = 8.6 Hz, 2H), 4.24 – 4.11 (m, 2H), 3.79 (s, 3H), 2.34 (ddd, J = 10.4, 8.0, 2.4 Hz, 1H), 2.03 – 1.92 (m, 4H), 1.64 – 1.54 (m, 1H), 1.30 (t, J = 7.1 Hz, 3H). ^{13}**C{1H} NMR** (126 MHz, $CDCl_3$) δ 171.1, 170.3, 160.2, 131.3, 130.0, 114.5, 64.6, 61.7, 56.0, 28.0, 21.7, 20.7, 14.9 ppm. **HRMS** (ESI) *m/z* calculated for $C_{15}H_{19}O_5^+$ [M+H]$^+$ 279.1232, found 279.1220.

(1S,2R)-2-cyanocyclopropyl benzoate (7a)

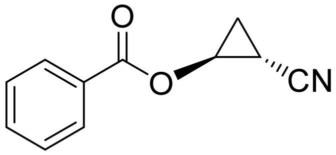

Following the general procedure E, *trans* isomer of **7a** was isolated as a white solid. (133 mg, 53% yield).

^{1}H NMR (500 MHz, $CDCl_3$) δ 7.97 (dt, J = 8.5, 1.1 Hz, 2H), 7.60 (td, J = 7.4, 1.3 Hz, 1H), 7.45 (ddd, J = 8.7, 7.5, 1.2 Hz, 2H), 4.74 (ddt, J = 6.8, 4.9, 2.7 Hz, 1H), 1.84 – 1.75 (m, 1H), 1.64 – 1.53 (m, 2H). ^{13}**C{1H} NMR** (126 MHz, $CDCl_3$) δ 166.8, 134.5, 130.4, 129.3, 119.4, 53.4, 14.5, 5.3. **HRMS** (ESI) *m/z* calculated for $C_{11}H_{10}NO_2^+$ [M+H]$^+$188.0707, found 188.0705.

Ethyl phenylglycinate (8a)

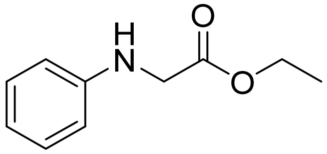

Following the general procedure D, **8a** was isolated as a colorless oil. (41 mg, 43% yield).

The analytical data are in accord with those reported in literature.[4]

^{1}H NMR (500 MHz, CDCl$_3$) δ 7.19 (td, *J* = 8.7, 2.5 Hz, 2H), 6.75 (t, *J* = 7.3 Hz, 1H), 6.61 (dd, *J* = 8.7, 2.2 Hz, 2H), 4.24 (q, *J* = 7.1 Hz, 2H), 3.89 (s, 2H), 1.29 (t, *J* = 7.1 Hz, 3H)

Ethyl phenylalaninate (8b)

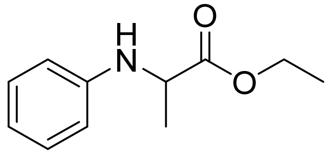

Following the general procedure D, **8b** was isolated as a colorless oil. (39 mg, 38% yield).

The analytical data are in accord with those reported in literature.[4]

^{1}H NMR (500 MHz, CDCl$_3$) δ 7.17 (td, *J* = 7.3, 1.4 Hz, 2H), 6.74 (td, *J* = 7.3, 1.0 Hz, 1H), 6.61 (d, *J* = 8.6 Hz, 2H), 4.18 (q, *J* = 7.1 Hz, 2H), 4.13 (q, *J* = 6.9 Hz, 1H), 1.47 (d, *J* = 6.9 Hz, 2H), 1.25 (t, *J* = 7.1 Hz, 3H).

Ethyl 2-(phenylthio)acetate (9a)

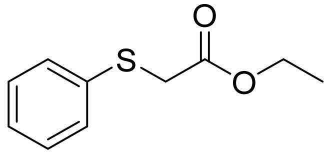

Following the general procedure D, **9a** was isolated as a colorless oil. (44 mg, 50% yield).

The analytical data are in accord with those reported in literature.[5]

^{1}H NMR (500 MHz, CDCl$_3$) δ 7.46 – 7.31 (m, 2H), 7.32 – 7.25 (m, 2H), 7.25 – 7.19 (m, 1H), 4.14 (q, *J* = 7.1 Hz, 2H), 3.62 (s, 2H), 1.20 (t, *J* = 7.1 Hz, 3H).

Ethyl 2-(phenylthio)propanoate (9b)

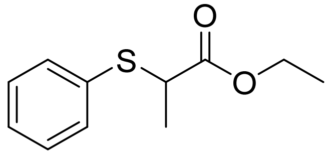

Following the general procedure D, **9b** was isolated as a colorless oil. The analytical data are in accord with those reported in literature.[5]

^{1}H NMR (400 MHz, CDCl$_3$) δ 7.46 (d, J = 6.8 Hz, 2H) 7.31-7.28 (m, 3H), 4.13 (q, J = 7.2 Hz, 2H), 3.80 (q, J = 7.2 Hz, 1H), 1.48 (d, J = 7.2 Hz, 3H), 1.18 (t, J = 7.2 Hz, 3H) ppm

Ethyl (S)-2-(phenylthio)pent-4-enoate (10a)

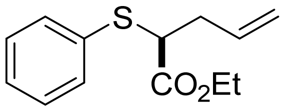

Following the general procedure D, **10a** was isolated as a colorless oil. (26 mg, 21% yield).

The analytical data are in accord with those reported in literature.[6]

^{1}H NMR (500 MHz, CDCl$_3$) δ 7.49 – 7.42 (m, 2H), 7.35 – 7.22 (m, 3H), 5.79 (ddt, *J* = 17.1, 10.2, 6.9 Hz, 1H), 5.18 – 5.02 (m, 2H), 4.10 (pq, *J* = 6.4, 4.0 Hz, 2H), 3.68 (dd, *J* = 8.7, 6.3 Hz, 1H), 2.61 (dddt, *J* = 15.6, 8.3, 7.0, 1.3 Hz, 1H), 2.50 (dtt, *J* = 14.3, 6.3, 1.3 Hz, 1H), 1.15 (t, *J* = 7.1 Hz, 3H).

2-hydroxy-2-isopropylcyclopropane-1-carboxylic acid (11)

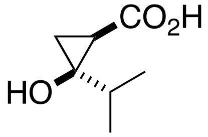

Following the general procedure F, **11** was isolated as a colorless oil. (2 mg, 29% yield).

^{1}H NMR (500 MHz, CDCl$_3$) δ 3.86 (s, 2H), 2.77 (s, 2H), 2.63 (d, *J* = 6.7 Hz, 2H), 1.12 (d, *J* = 6.9 Hz, 6H). ***^{13}C{1H}* NMR** (126 MHz, CDCl$_3$) δ 166.2, 40.8, 34.7, 32.9, 29.7, 1 **HRMS** (ESI) *m/z* calculated for $C_7H_{13}O_3^+$ [M+H]$^+$ 145.0865, found 145.0854.

NMR Spectra

4e. 3-Methylbut-1-en-2-yl Benzoate
^{1}H NMR (500 MHz, $CDCl_3$)

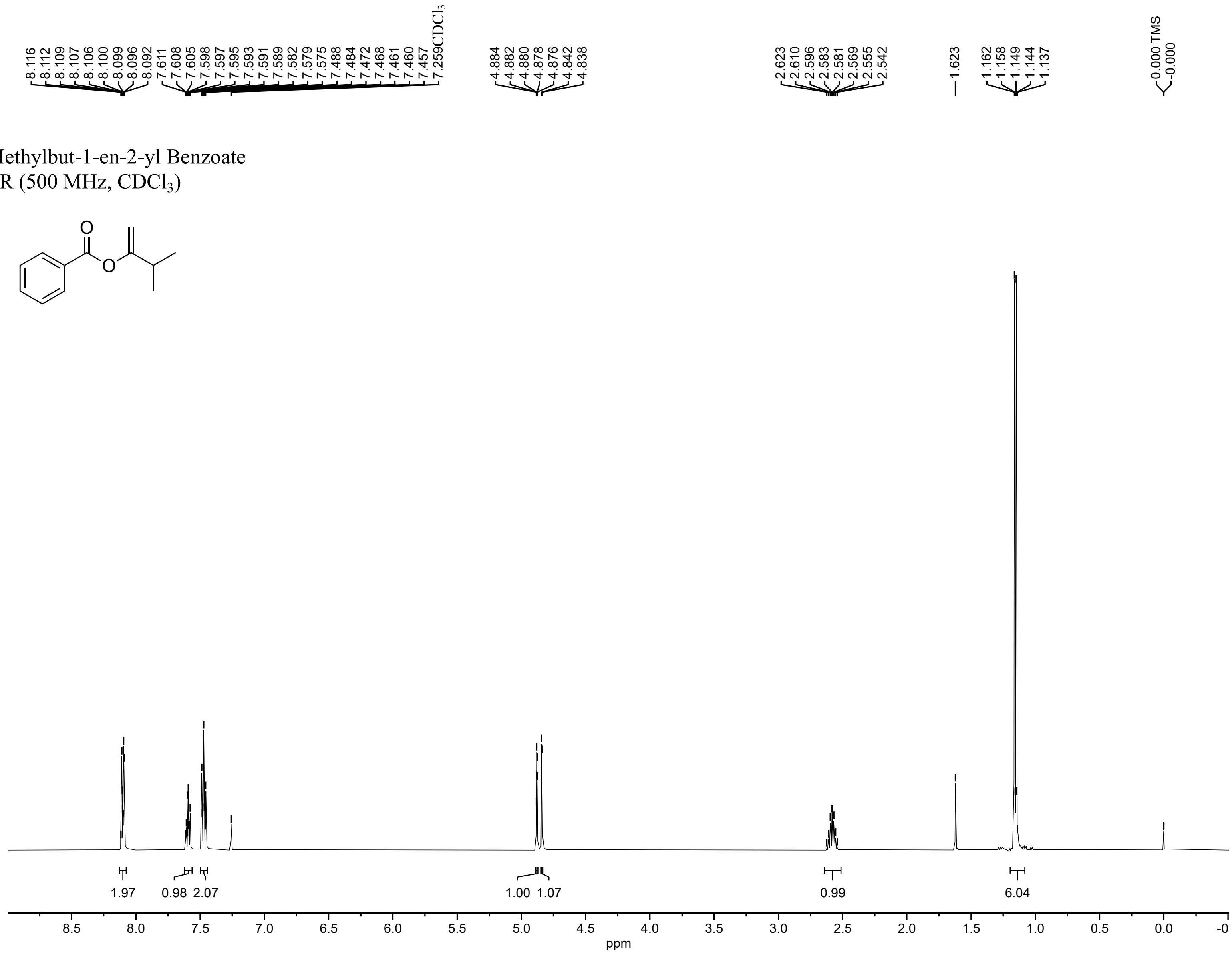

4e. 3-Methylbut-1-en-2-yl Benzoate
$^{13}C\{1H\}$ NMR (126 MHz, $CDCl_3$)

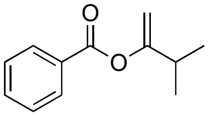

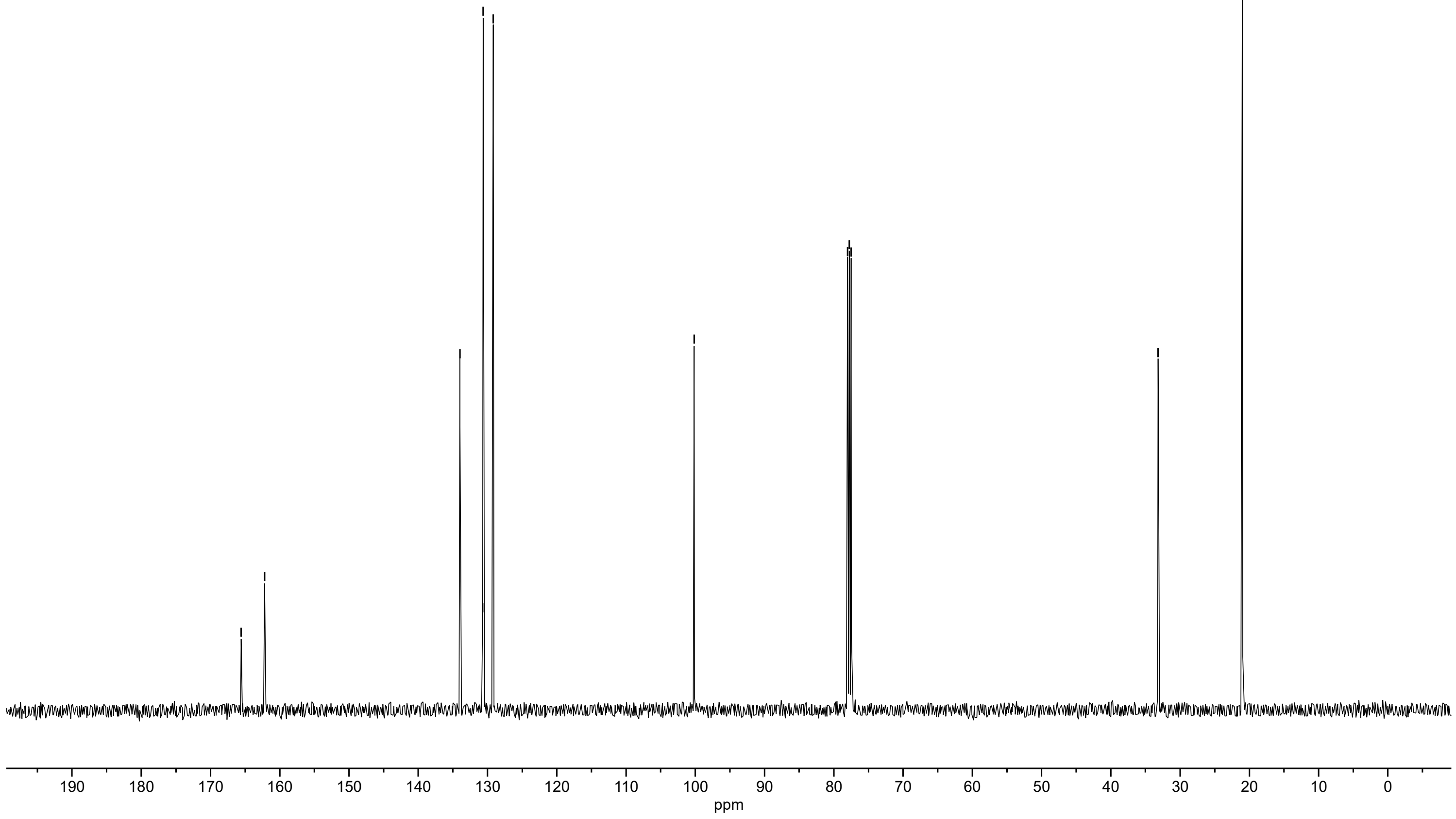

4g. 1-Cyclopentylvinyl Benzoate
^{1}H NMR (500 MHz, $CDCl_3$)

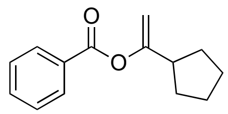

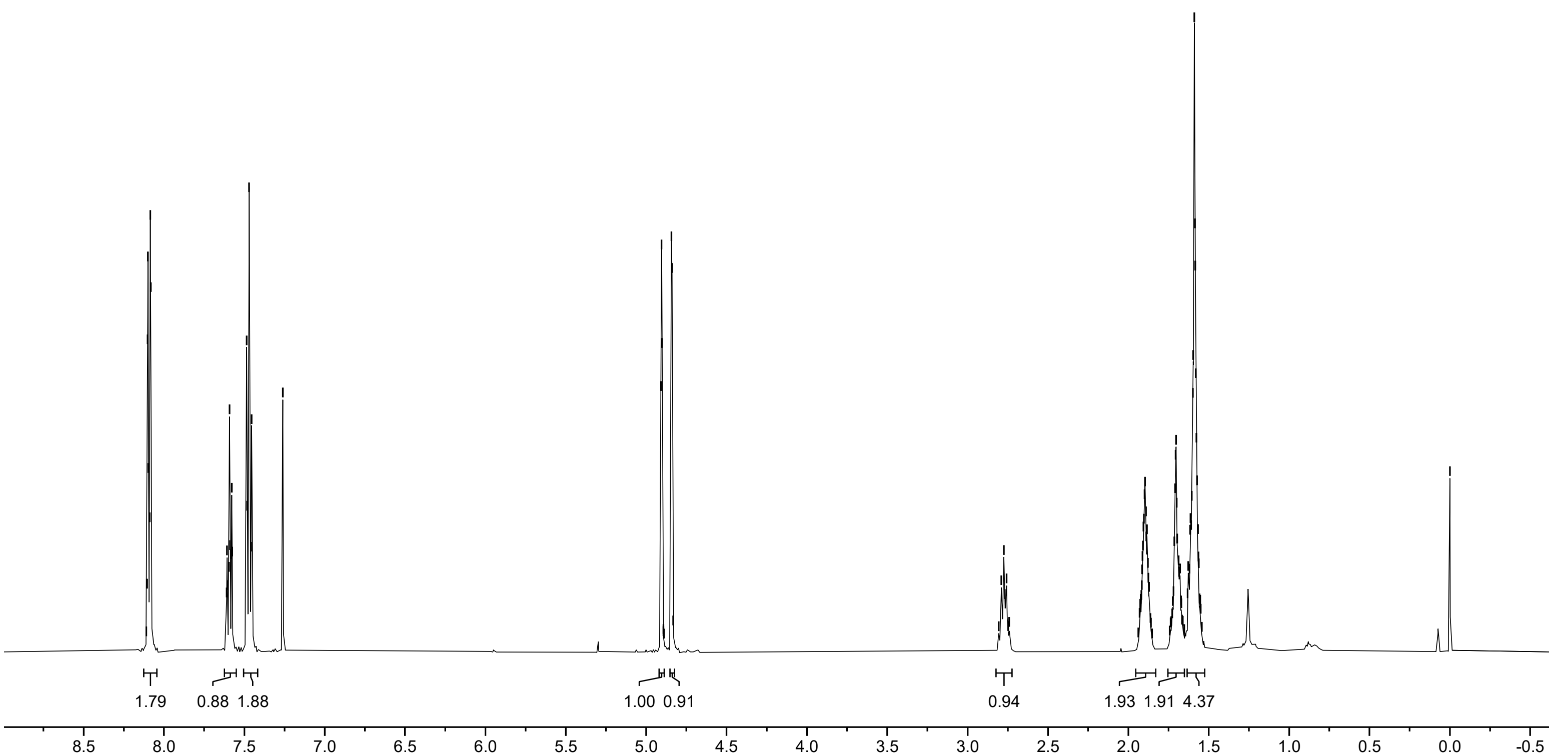

4g. 1-Cyclopentylvinyl Benzoate
$^{13}C\{1H\}$ NMR (126 MHz, $CDCl_3$)

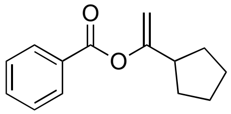

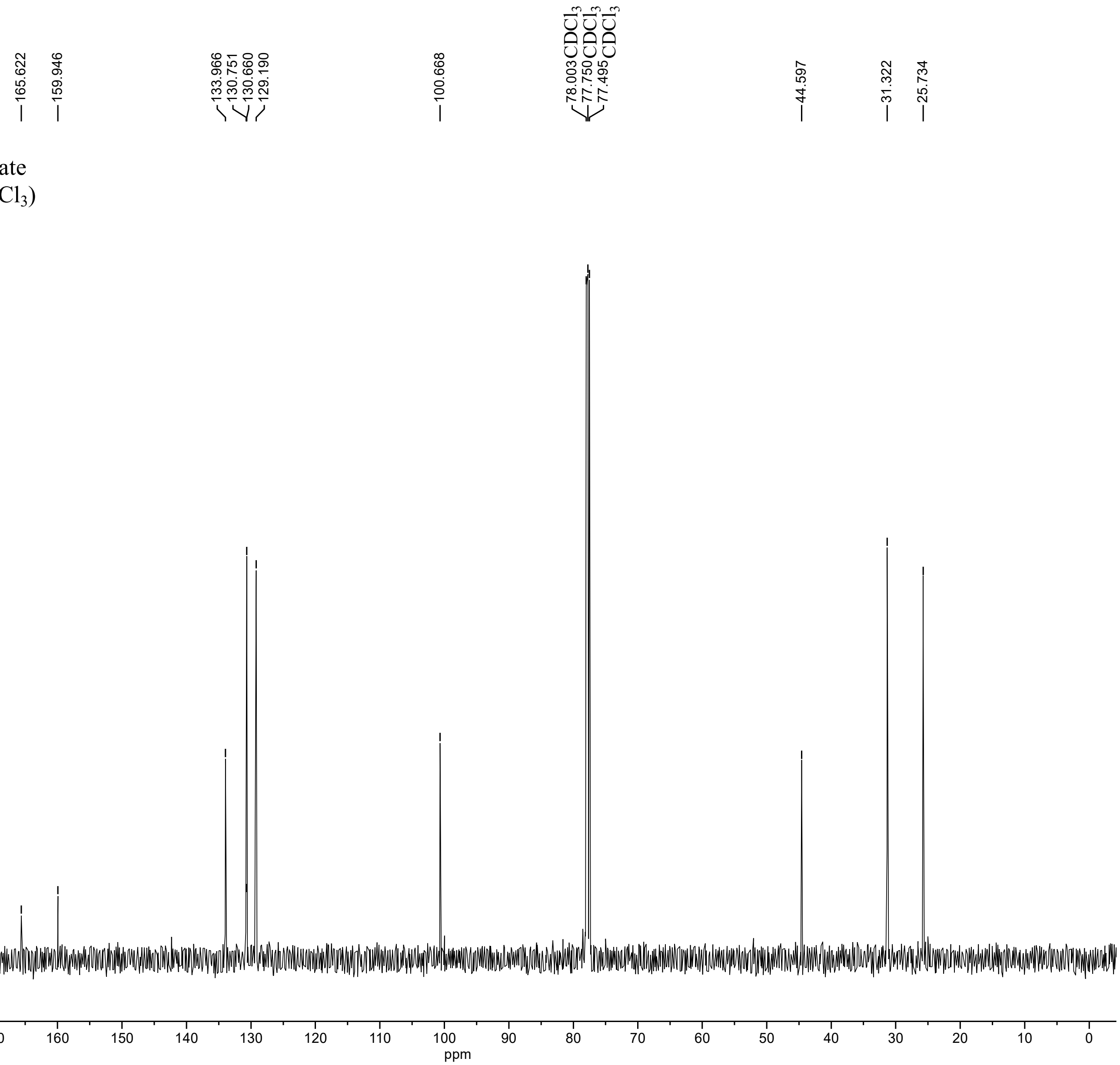

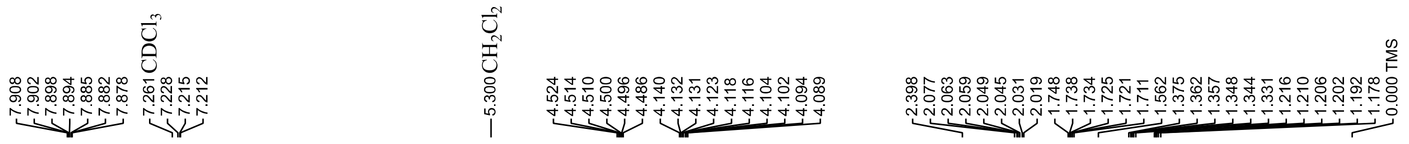

5b. (1S,2S)-2-(ethoxycarbonyl)cyclopropyl 4-methylbenzoate

^{1}H NMR (500 MHz, $CDCl_3$)

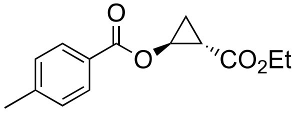

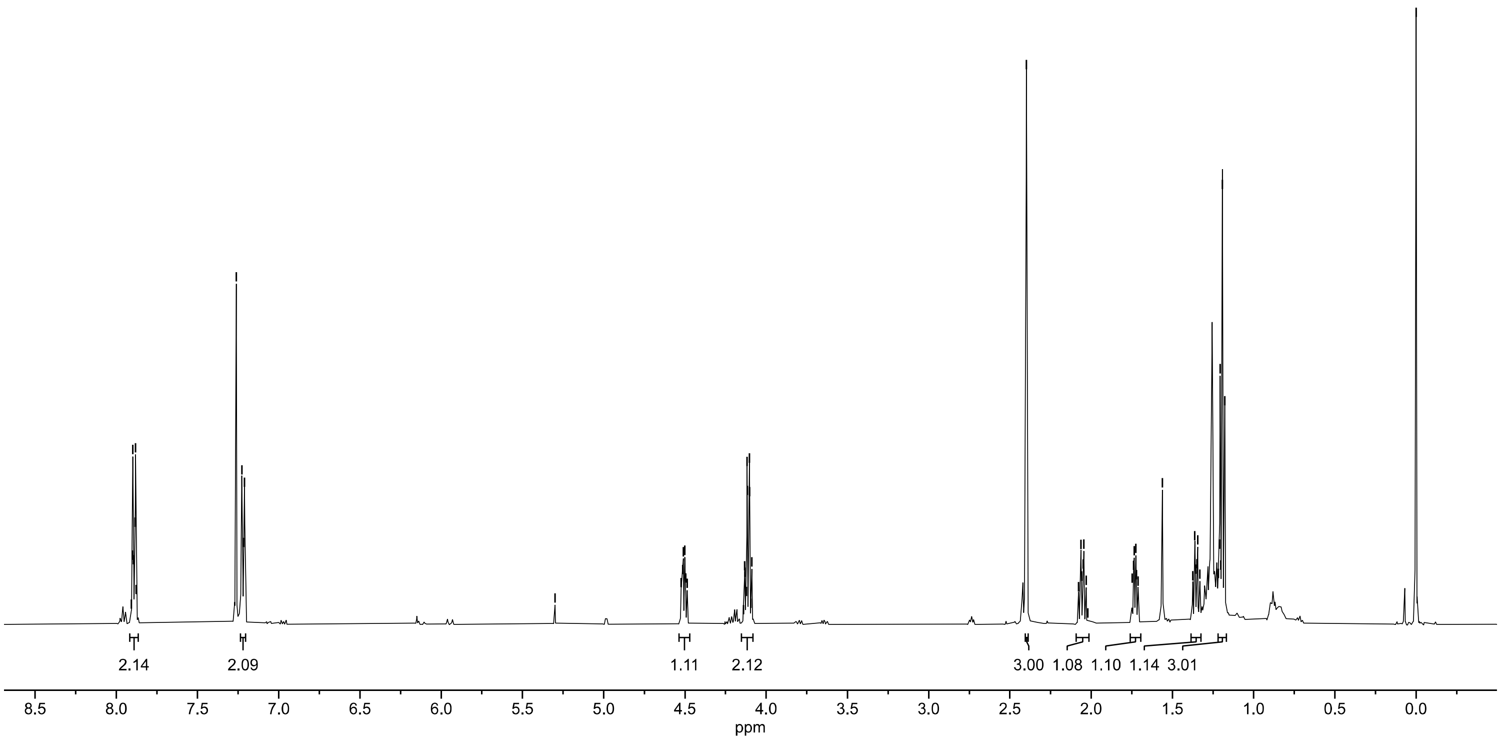

5b. (1S,2S)-2-(ethoxycarbonyl)cyclopropyl 4-methylbenzoate
$^{13}C\{1H\}$ NMR (126 MHz, $CDCl_3$)

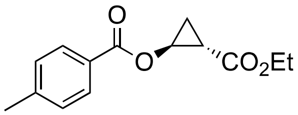

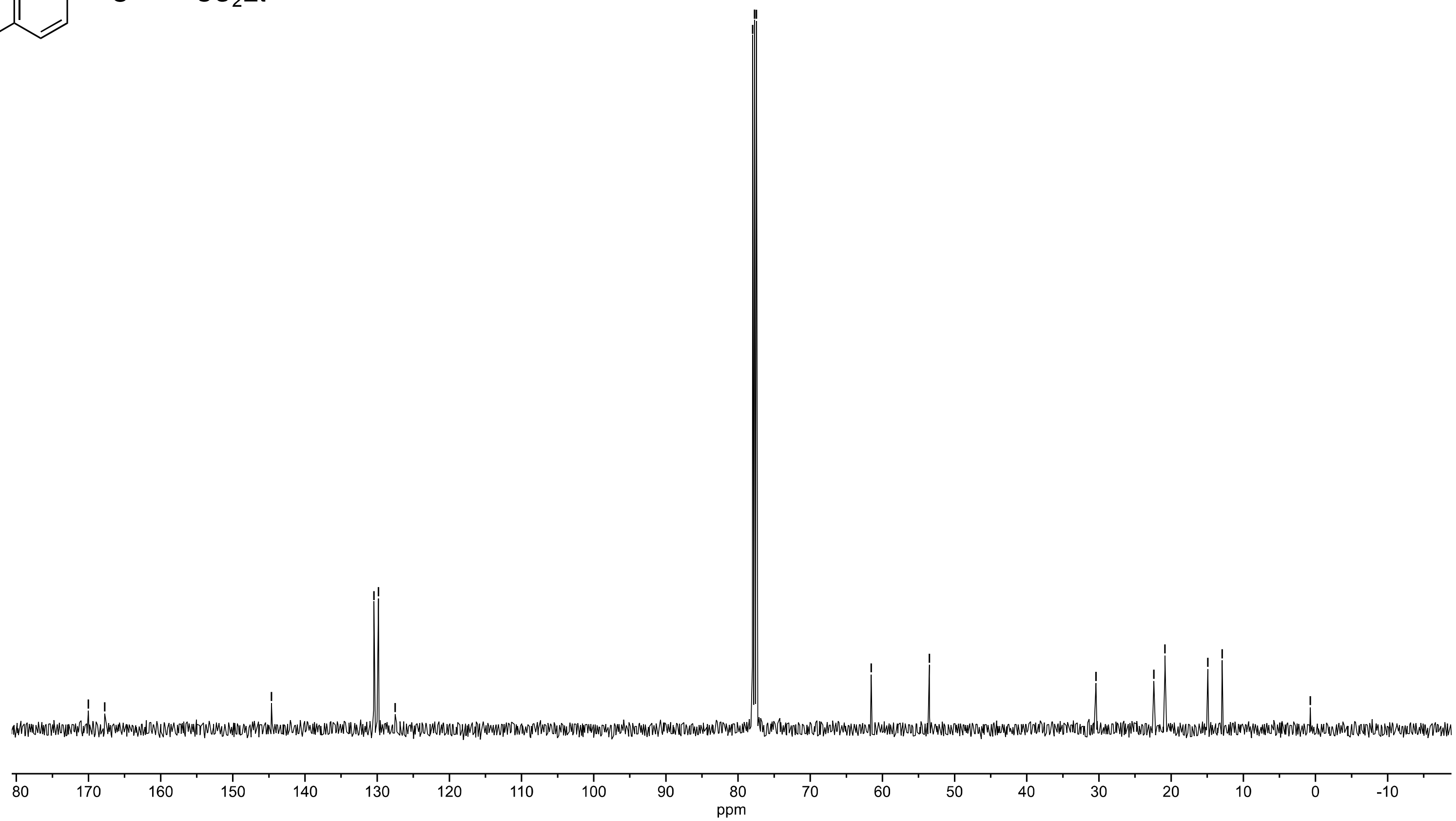

5c. (1S,2S)-2-(ethoxycarbonyl)cyclopropyl 4-methoxybenzoate
^{1}H NMR (500 MHz, $CDCl_3$)

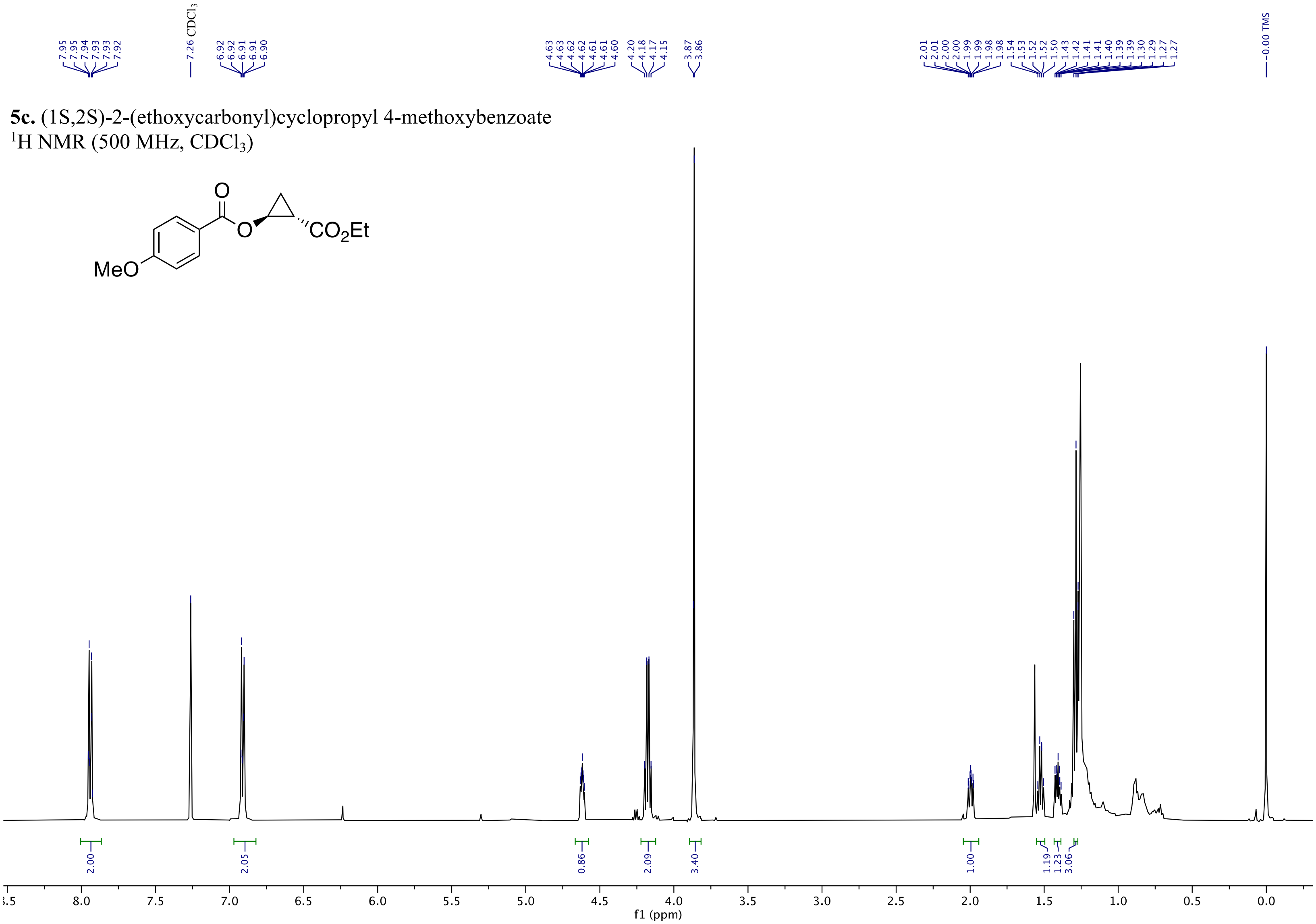

5c. (1S,2S)-2-(ethoxycarbonyl)cyclopropyl 4-methoxybenzoate
$^{13}C\{^{1}H\}$ NMR (126 MHz, $CDCl_3$)

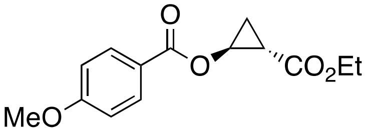

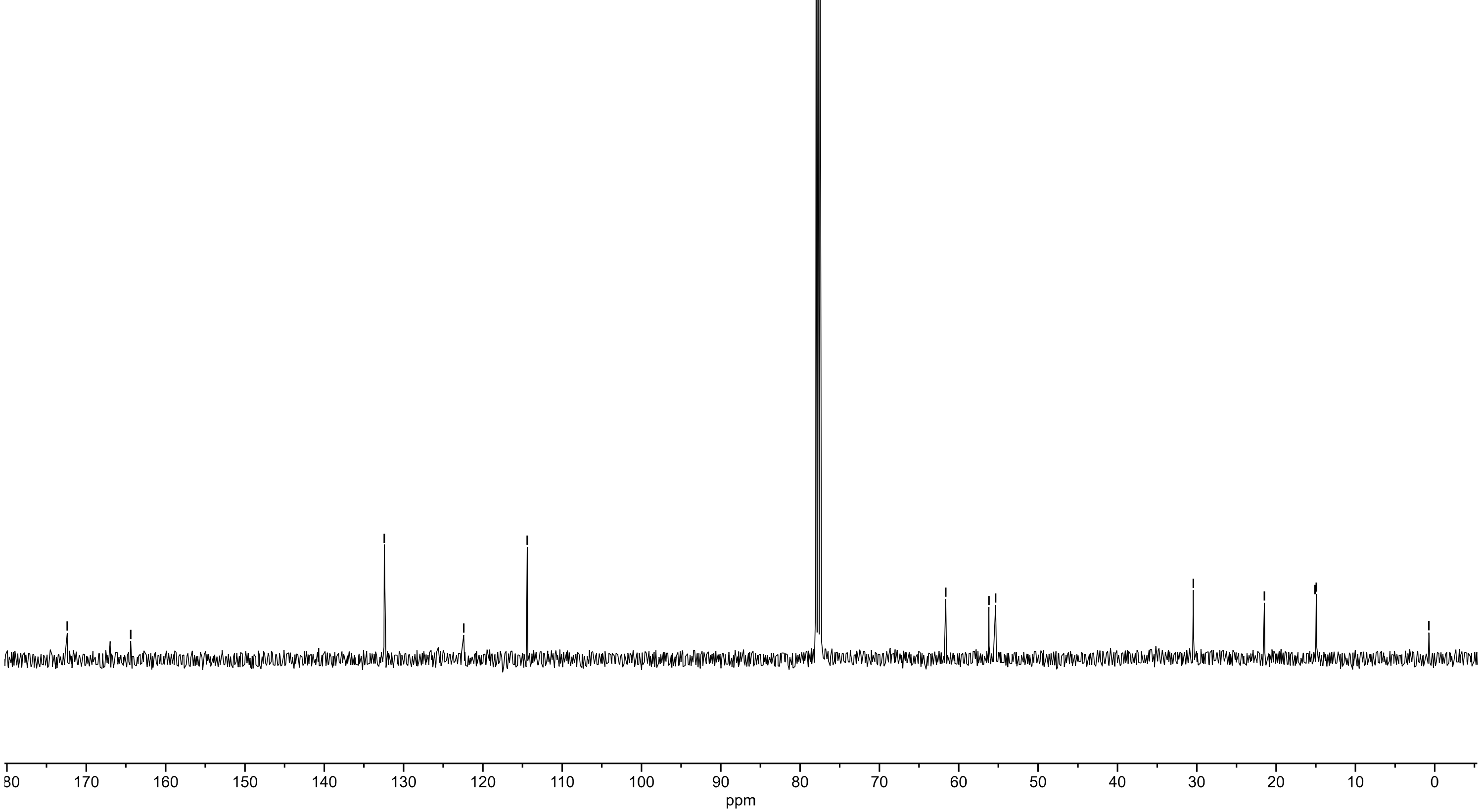

5d. (1*S*,2*R*)-1-butyl-2-(ethoxycarbonyl)cyclopropyl benzoate

^{1}H NMR (500 MHz, $CDCl_3$)

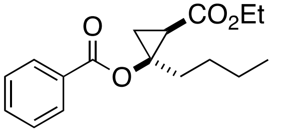

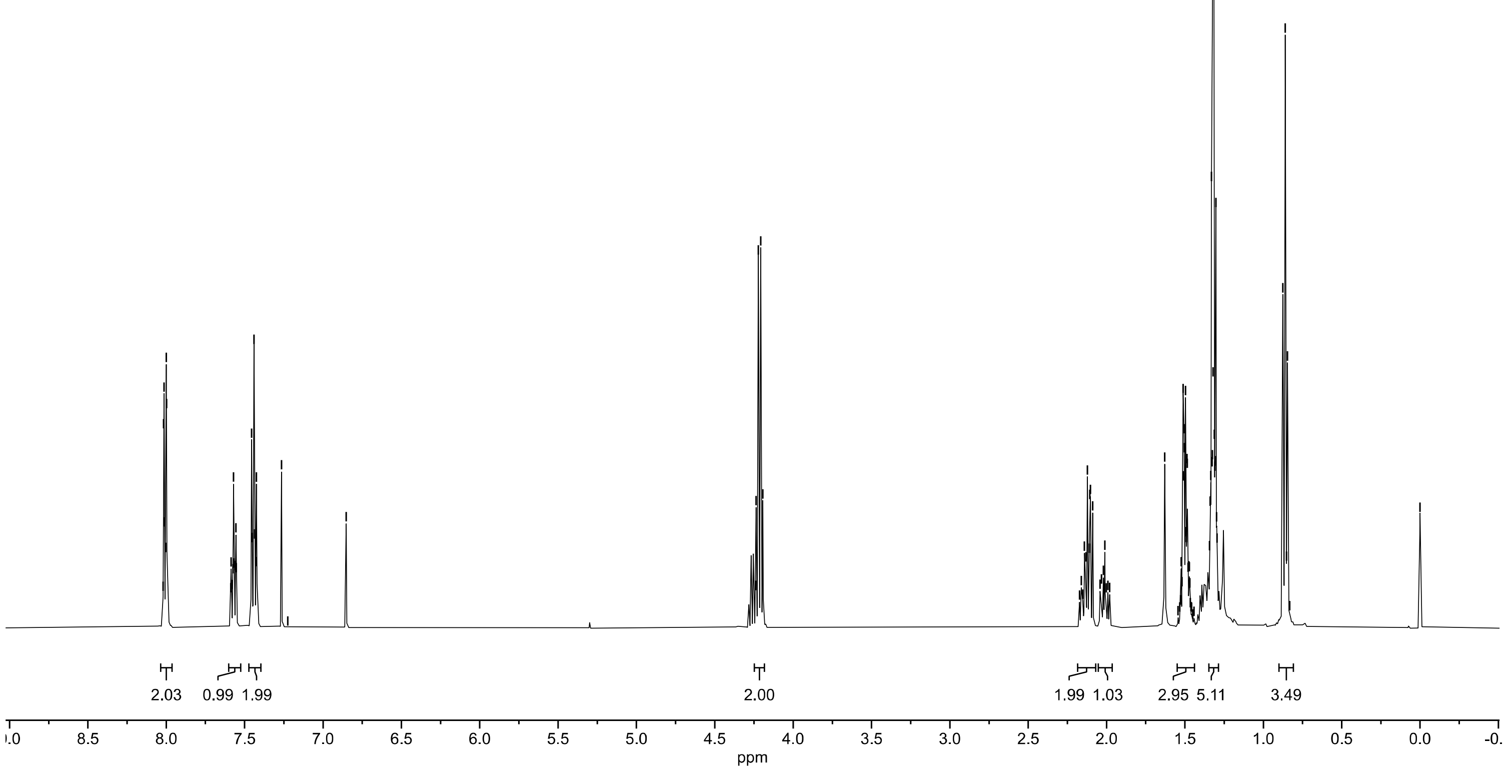

5d. (1*S*,2*R*)-1-butyl-2-(ethoxycarbonyl)cyclopropyl benzoate
^{13}C{*1H*} NMR (126 MHz, $CDCl_3$)

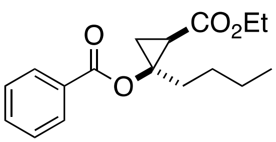

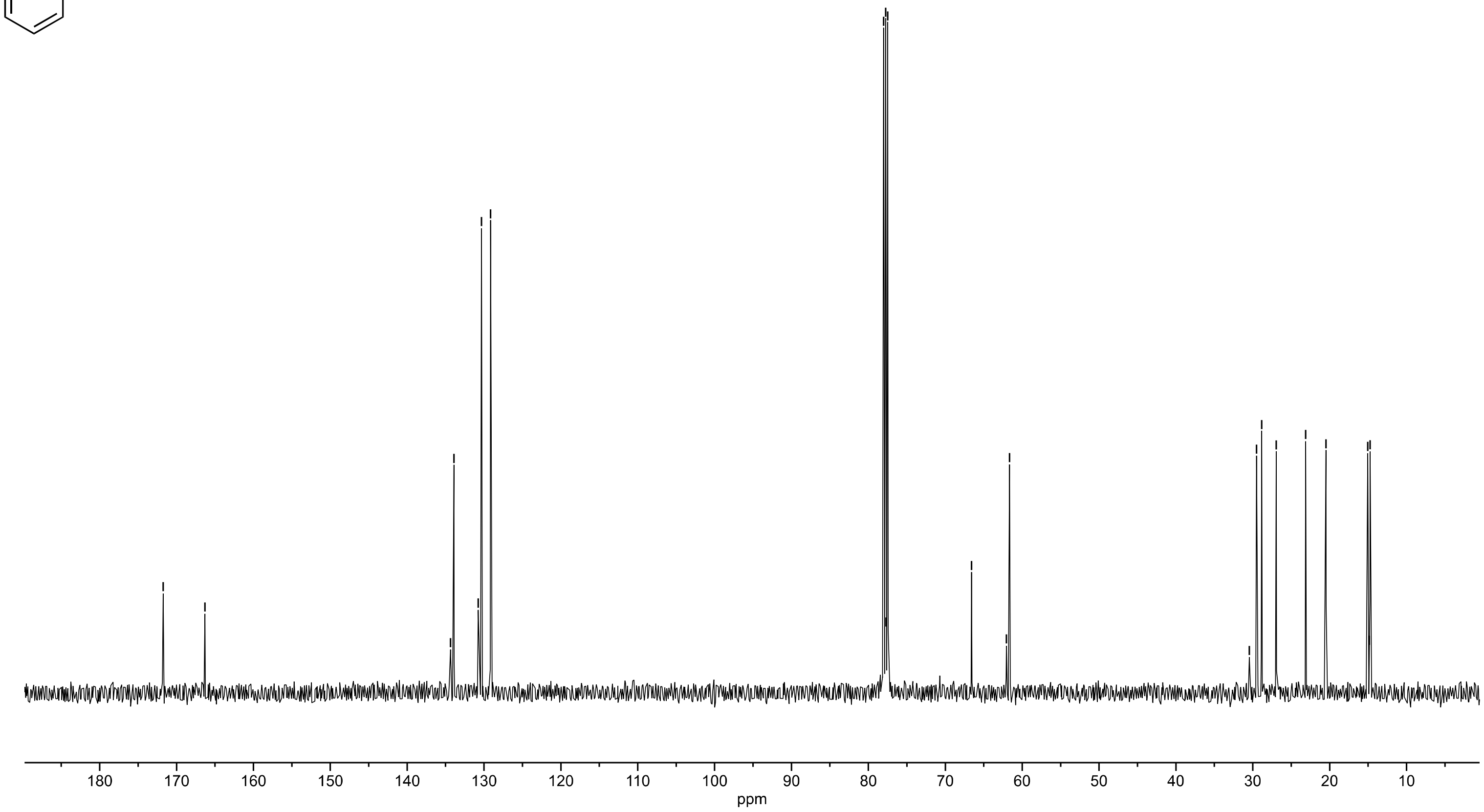

5e. (1R,2R)-2-(ethoxycarbonyl)-1-isopropylcyclopropyl benzoate
^{1}H NMR (500 MHz, $CDCl_3$)

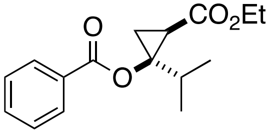

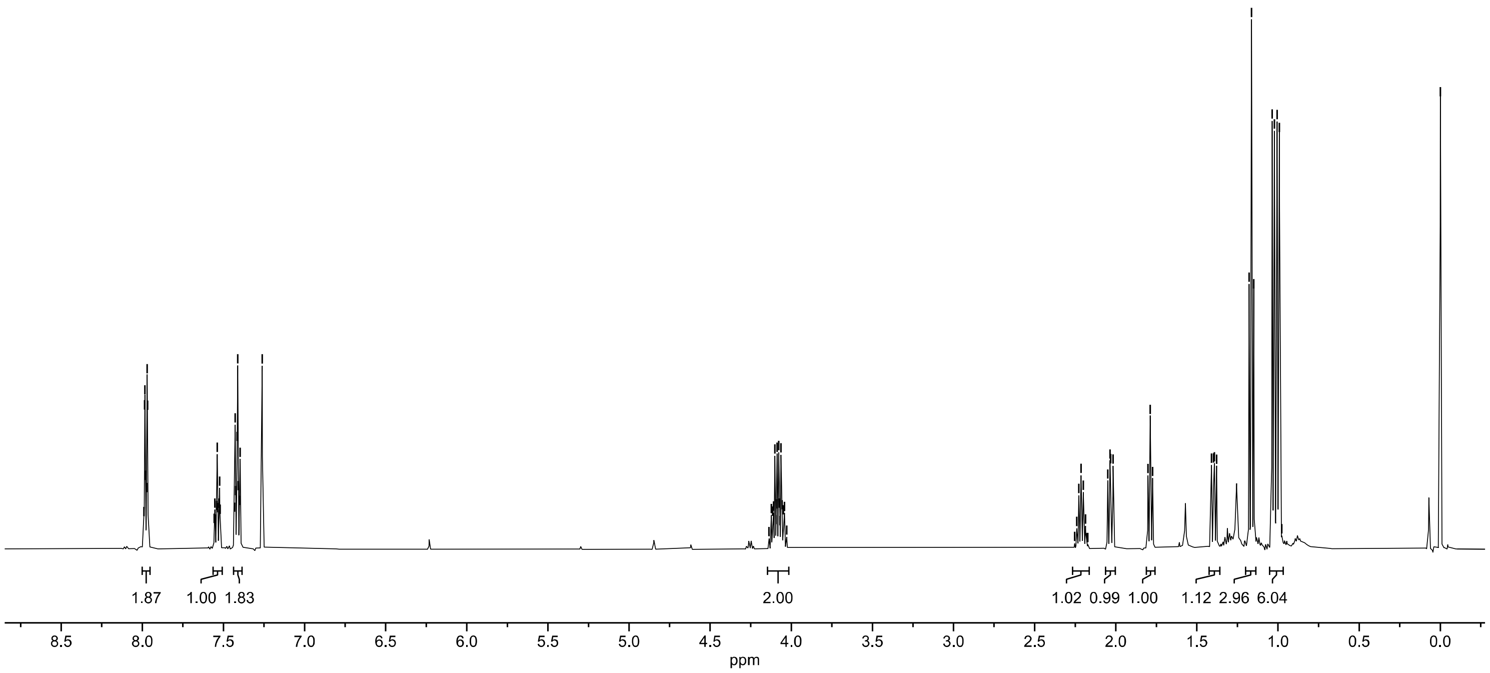

5e. (1R,2R)-2-(ethoxycarbonyl)-1-isopropylcyclopropyl benzoate
$^{13}C\{1H\}$ NMR (126 MHz, $CDCl_3$)

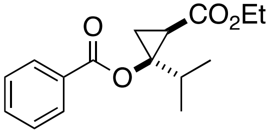

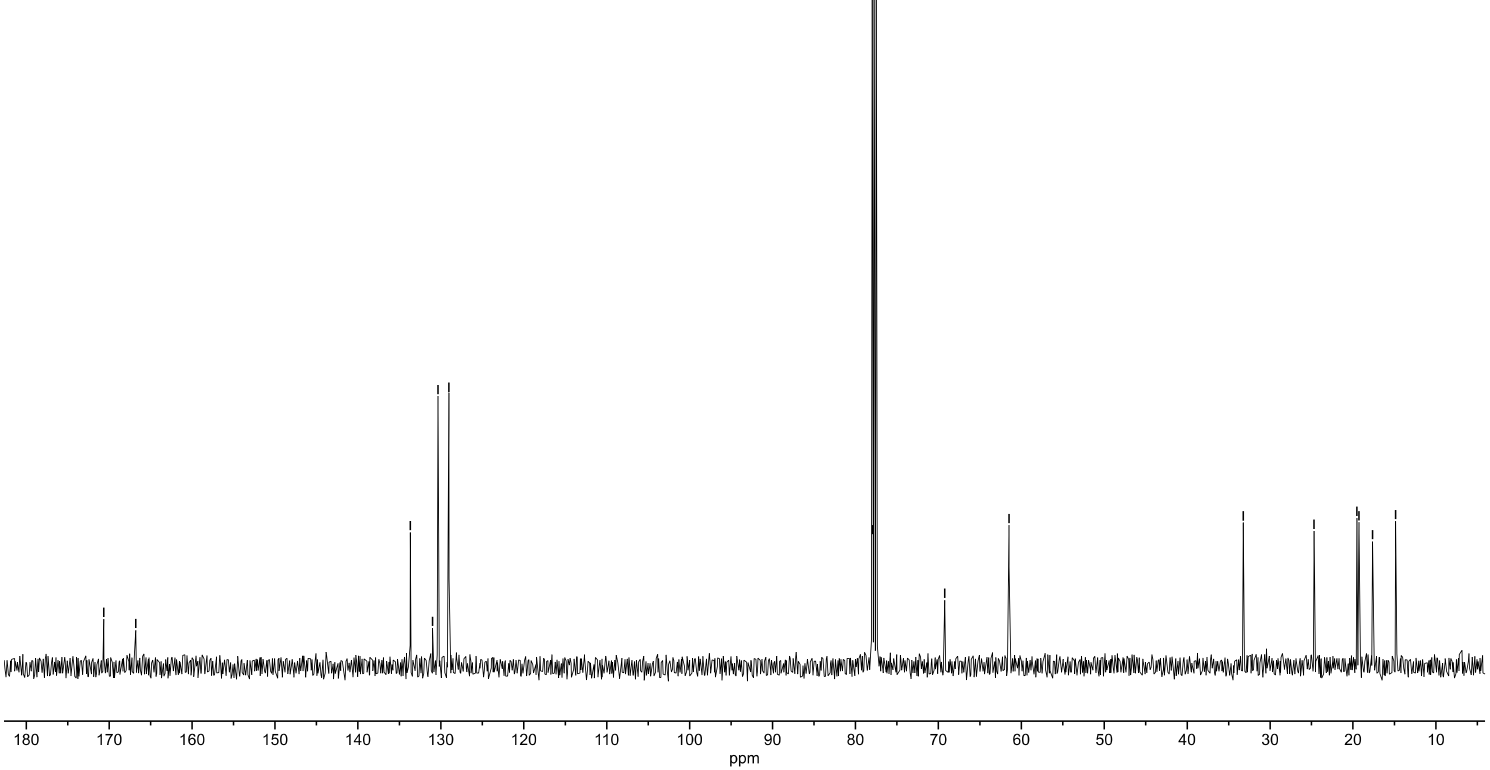

5f. (1R,2R)-2-(ethoxycarbonyl)-[1,1'-bi(cyclopropan)]-1-yl benzoate
^{1}H NMR (500 MHz, $CDCl_3$)

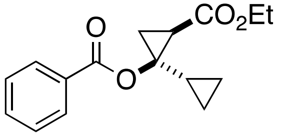

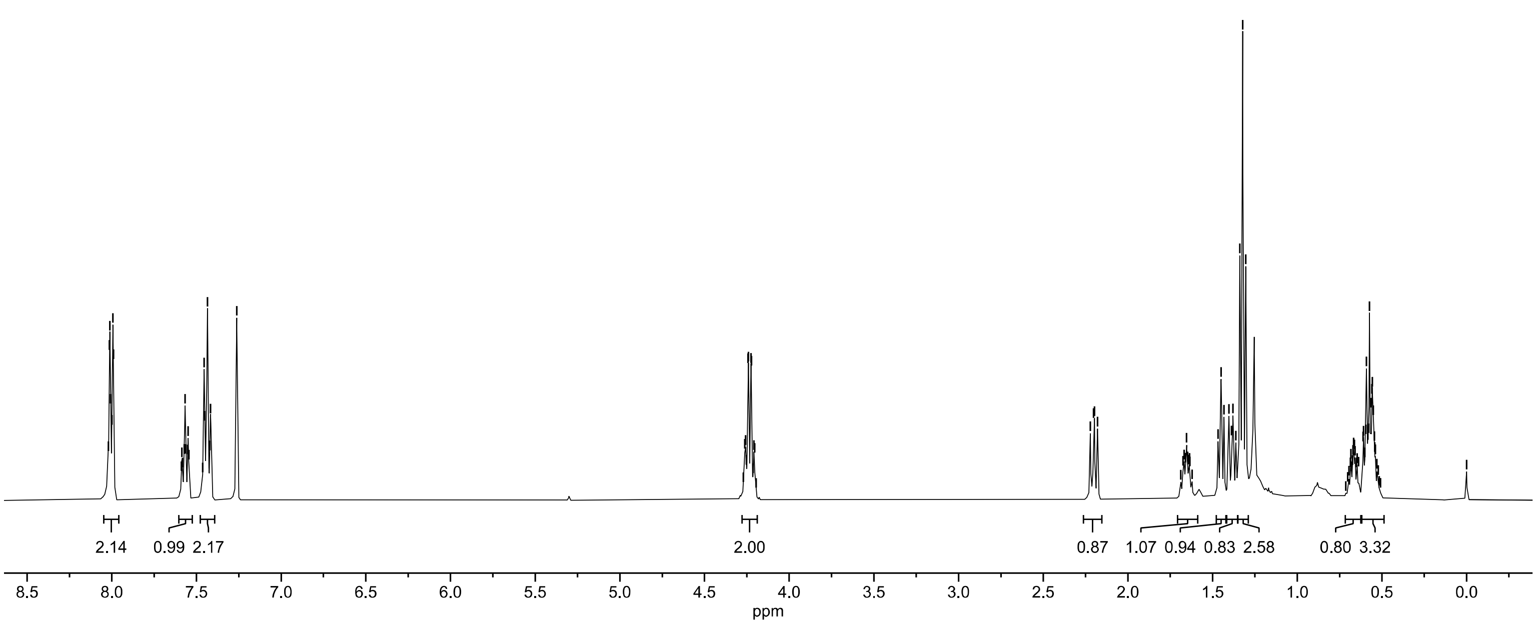

5f. (1R,2R)-2-(ethoxycarbonyl)-[1,1'-bi(cyclopropan)]-1-yl benzoate

$^{13}C\{1H\}$ NMR (126 MHz, $CDCl_3$)

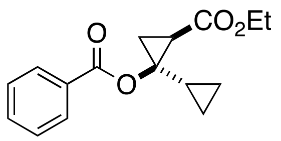

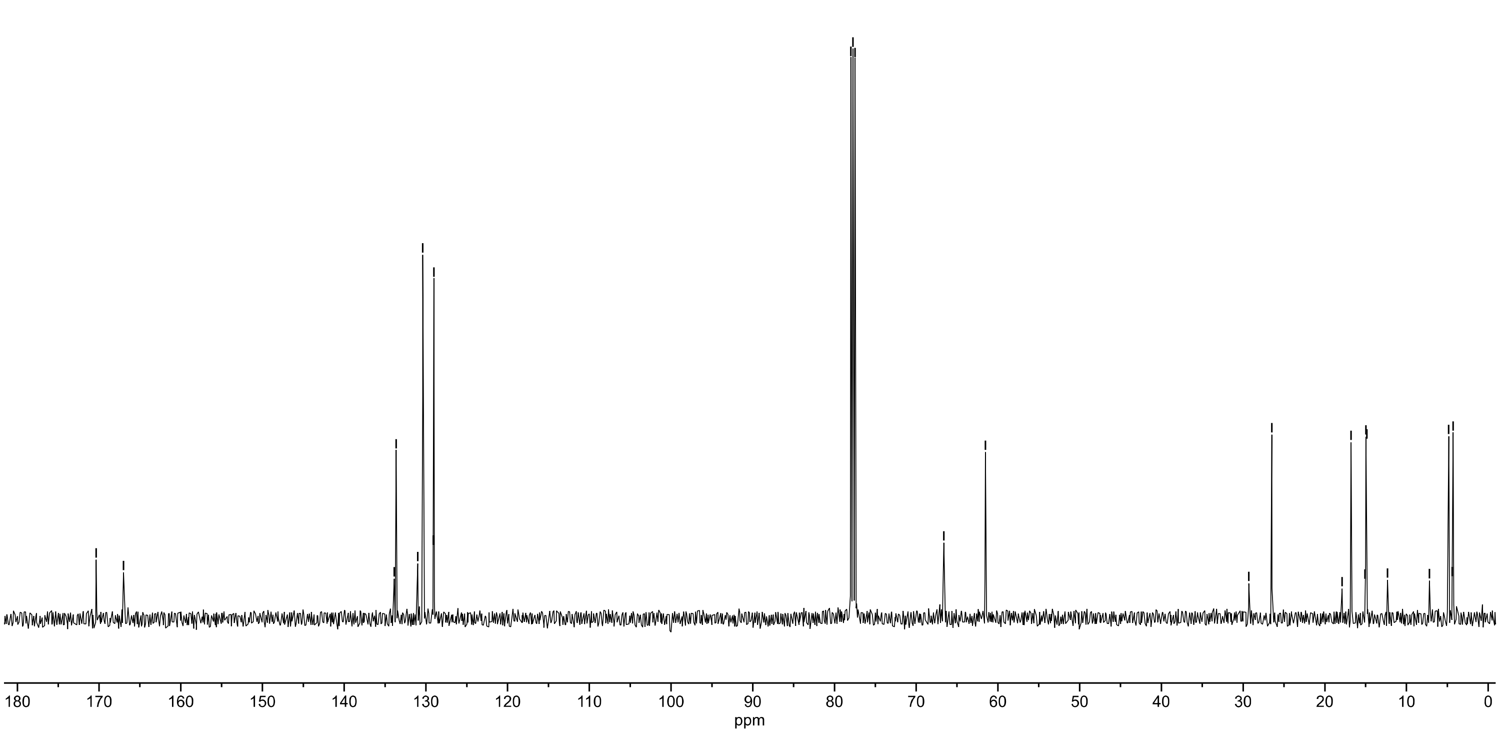

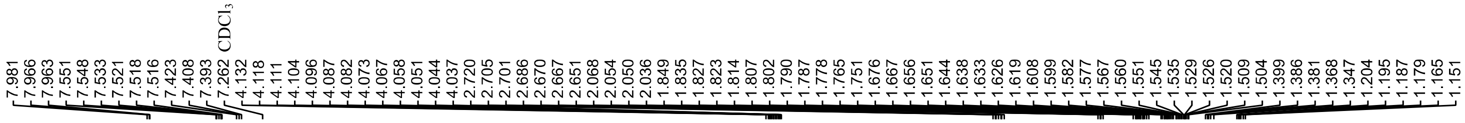

5g. (1R,2R)-1-cyclopentyl-2-(ethoxycarbonyl)cyclopropyl benzoate

^{1}H NMR (500 MHz, $CDCl_3$)

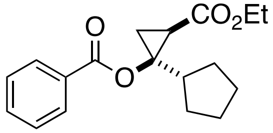

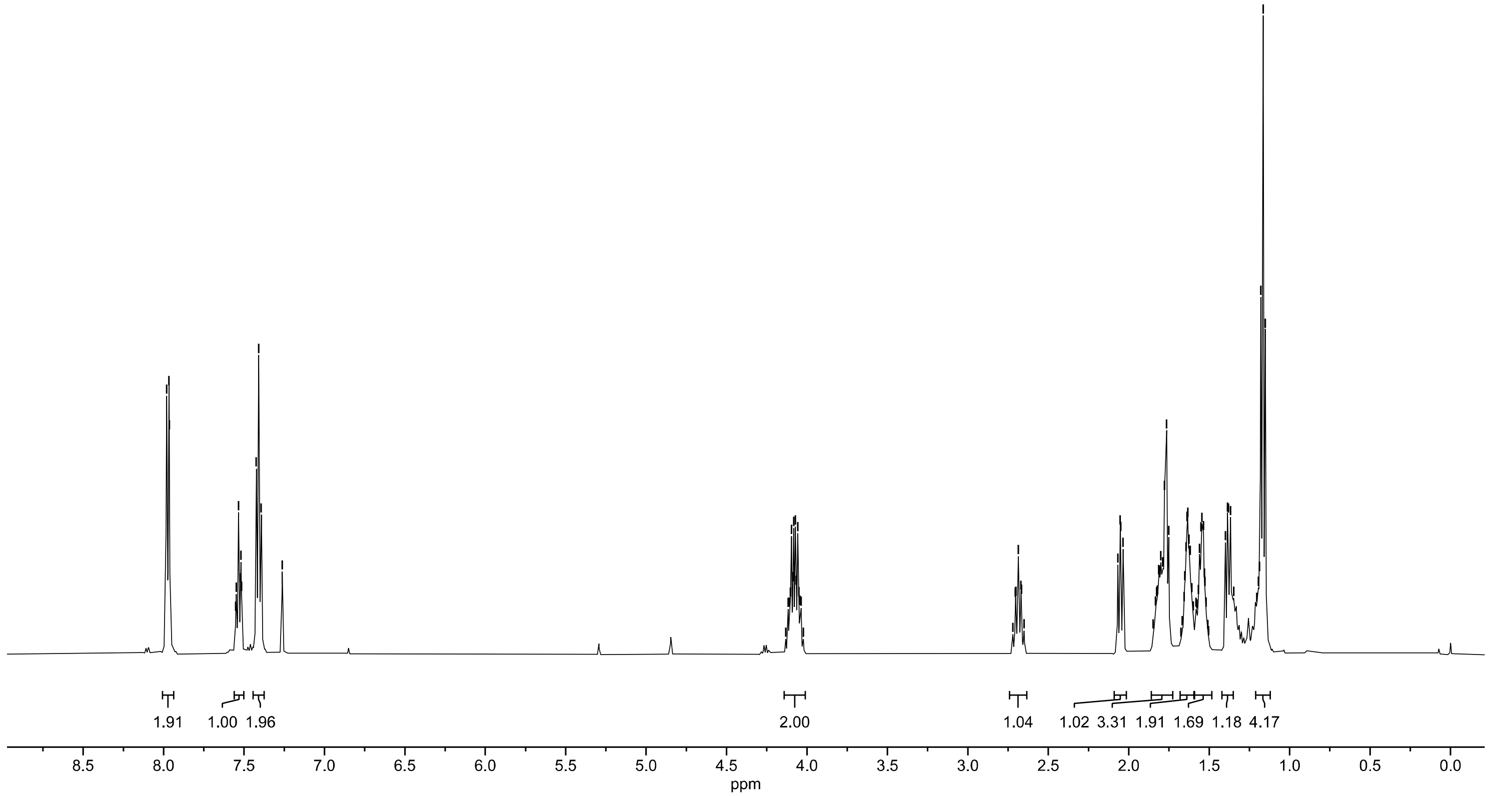

5g. (1R,2R)-1-cyclopentyl-2-(ethoxycarbonyl)cyclopropyl benzoate

$^{13}C\{^1H\}$ NMR (126 MHz, $CDCl_3$)

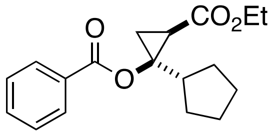

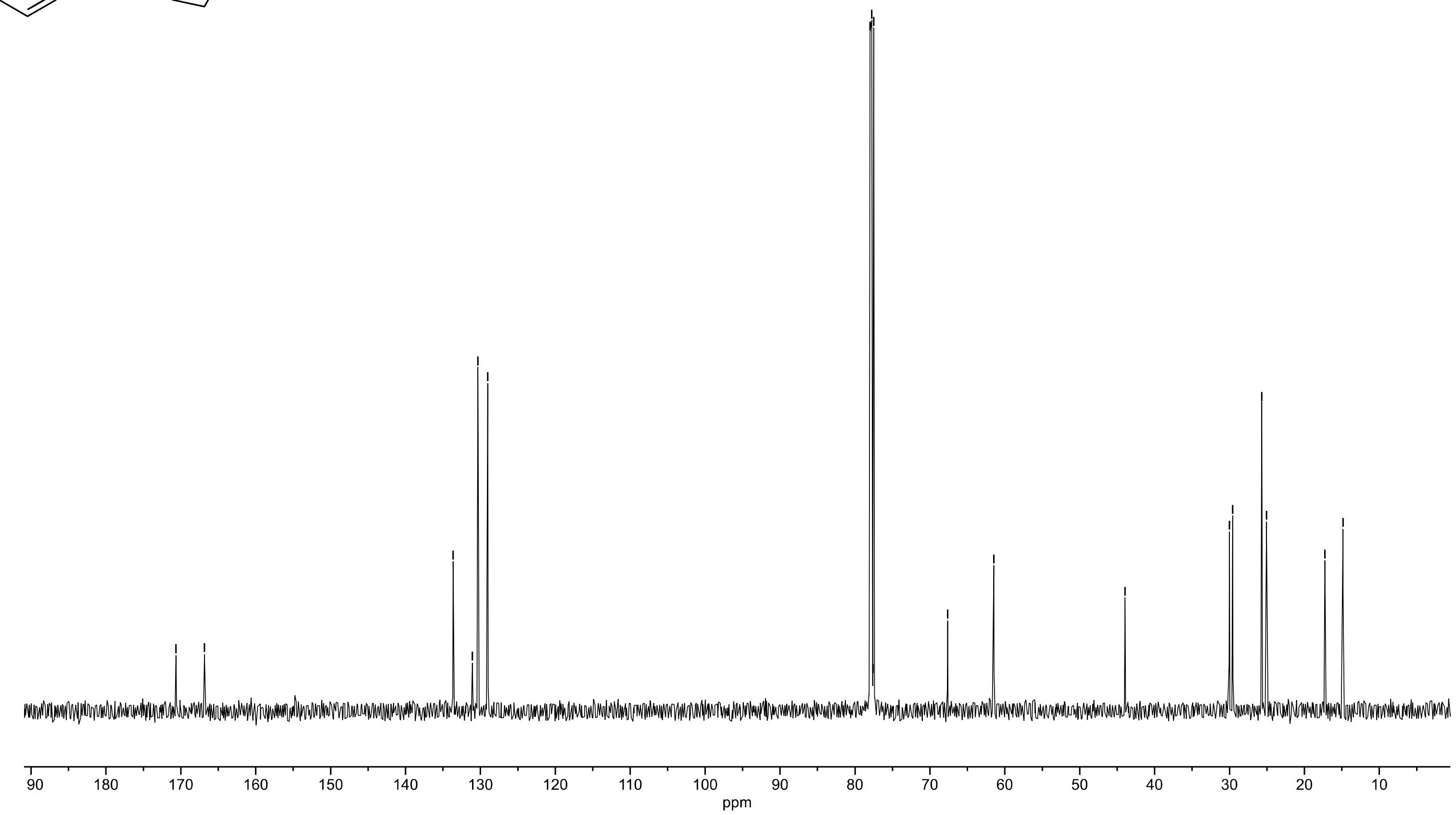

5h. (1R,2R)-1-cyclohexyl-2-(ethoxycarbonyl)cyclopropyl benzoate
^{1}H NMR (500 MHz, $CDCl_3$)

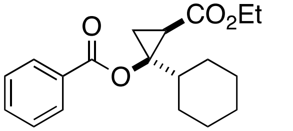

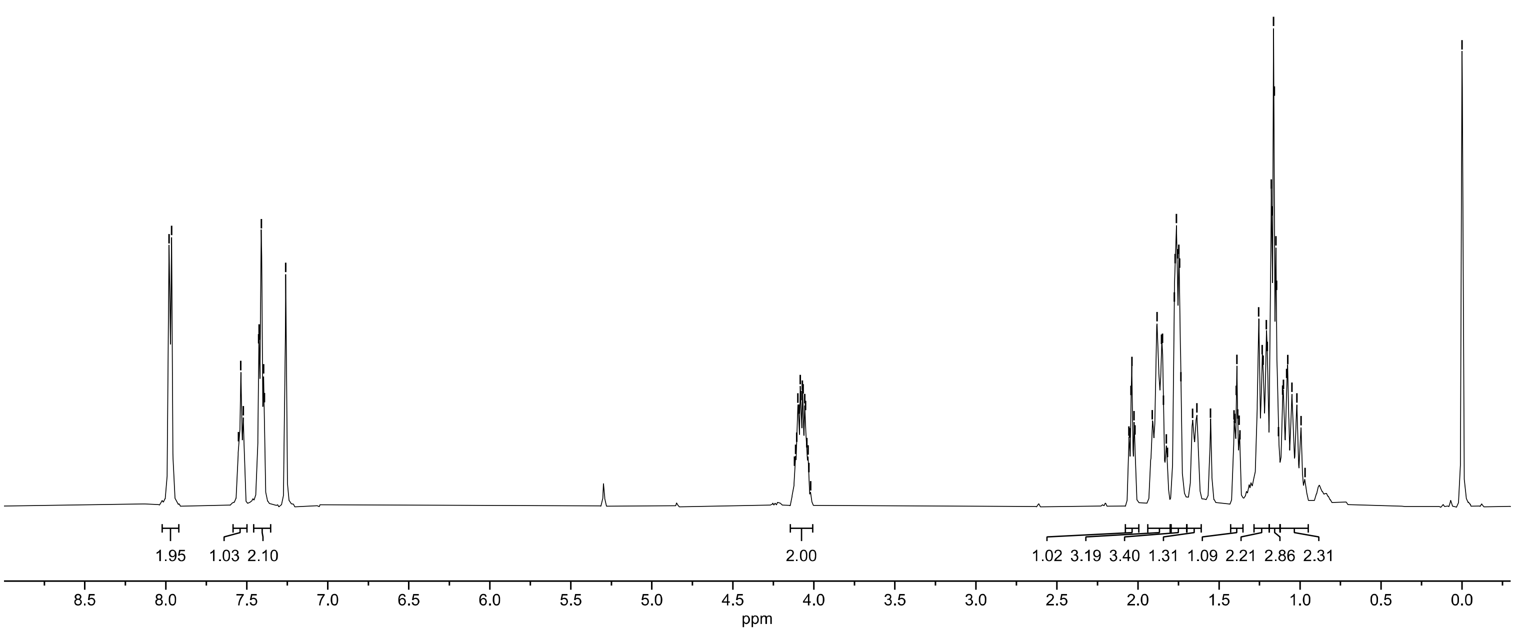

5h. (1R,2R)-1-cyclohexyl-2-(ethoxycarbonyl)cyclopropyl benzoate
$^{13}C\{^{1}H\}$ NMR (126 MHz, $CDCl_3$)

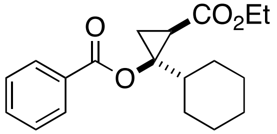

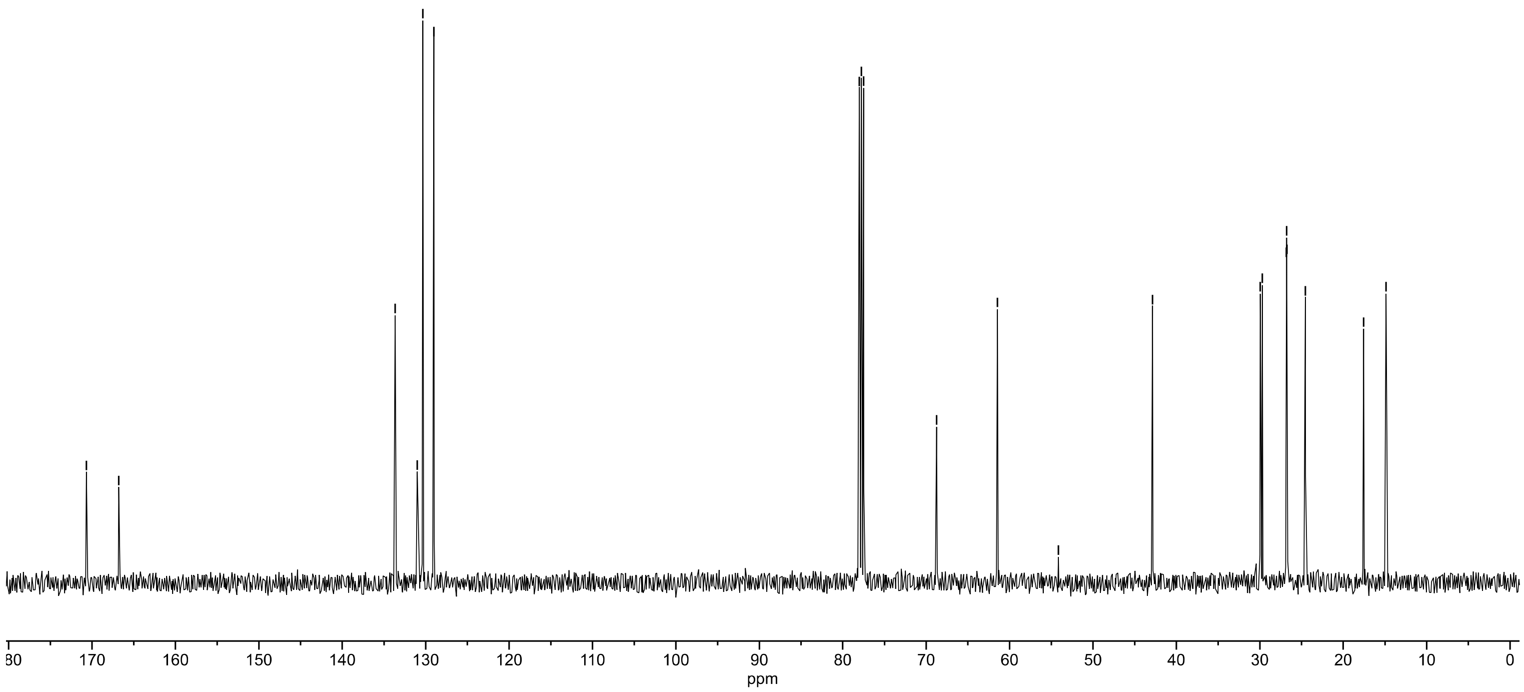

5i. 2-(ethoxycarbonyl)-1-phenylcyclopropyl benzoate
^{1}H NMR (500 MHz, $CDCl_3$)

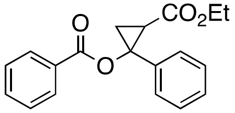

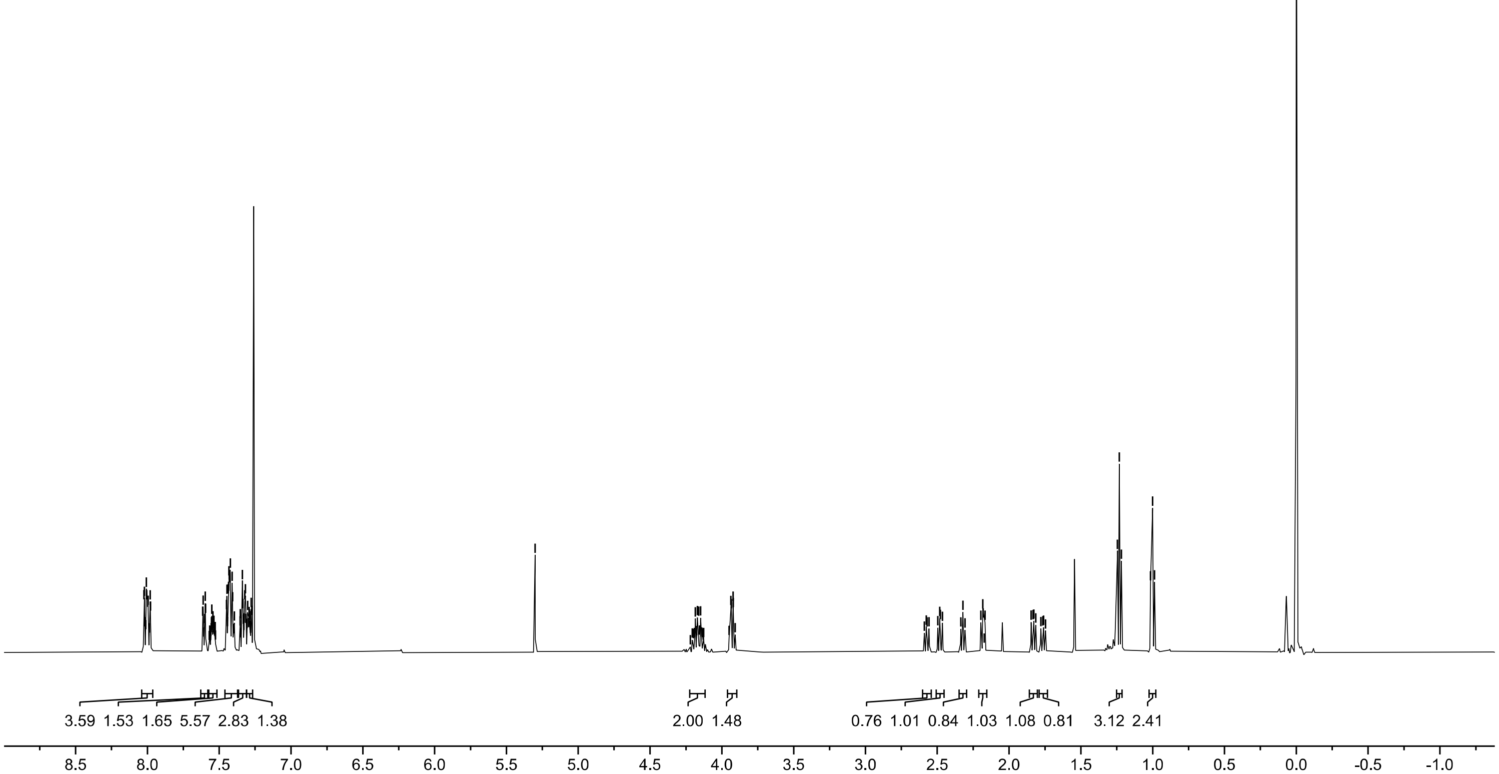

5j. Ethyl (1R,2S)-2-acetoxy-2-phenylcyclopropane-1-carboxylate
^{1}H NMR (500 MHz, $CDCl_3$)

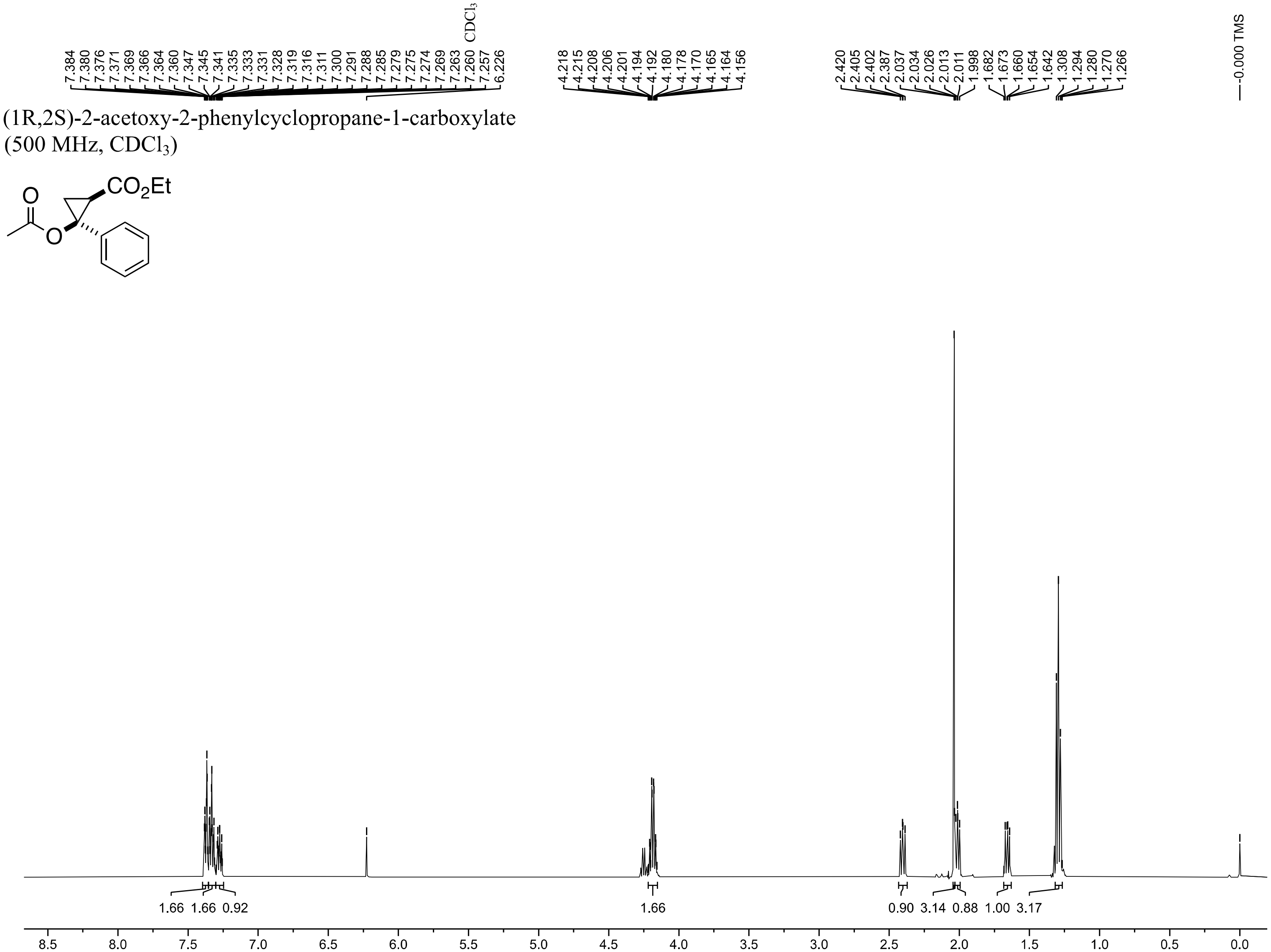

5j. Ethyl (1R,2S)-2-acetoxy-2-phenylcyclopropane-1-carboxylate

$^{13}C\{1H\}$ NMR (126 MHz, $CDCl_3$)

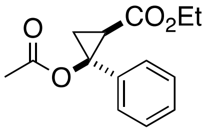

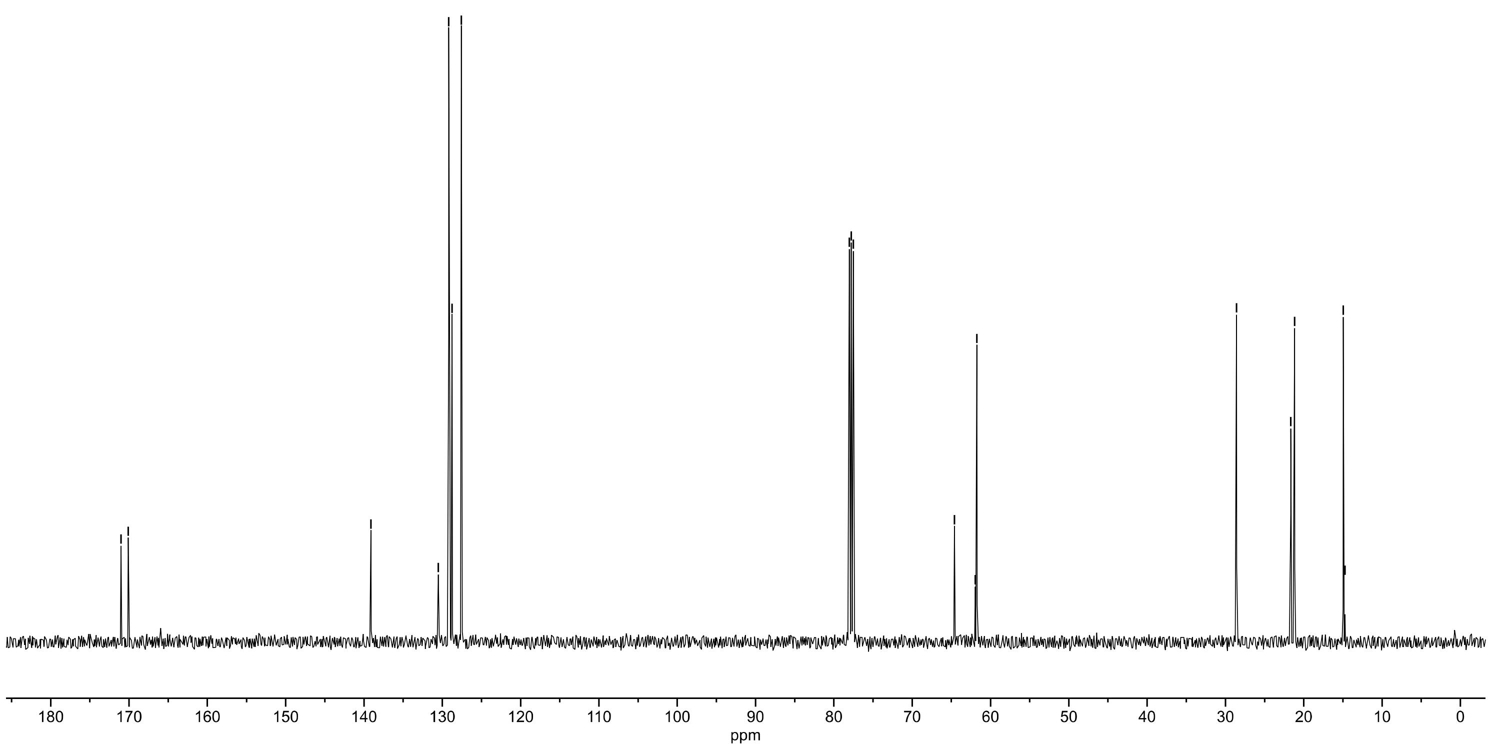

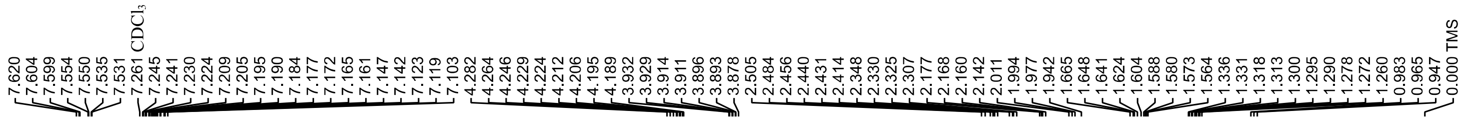

5k. Ethyl 2-acetoxy-2-(*o*-tolyl)cyclopropane-1-carboxylate

^{1}H NMR (500 MHz, $CDCl_3$)

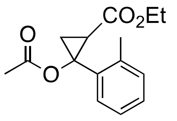

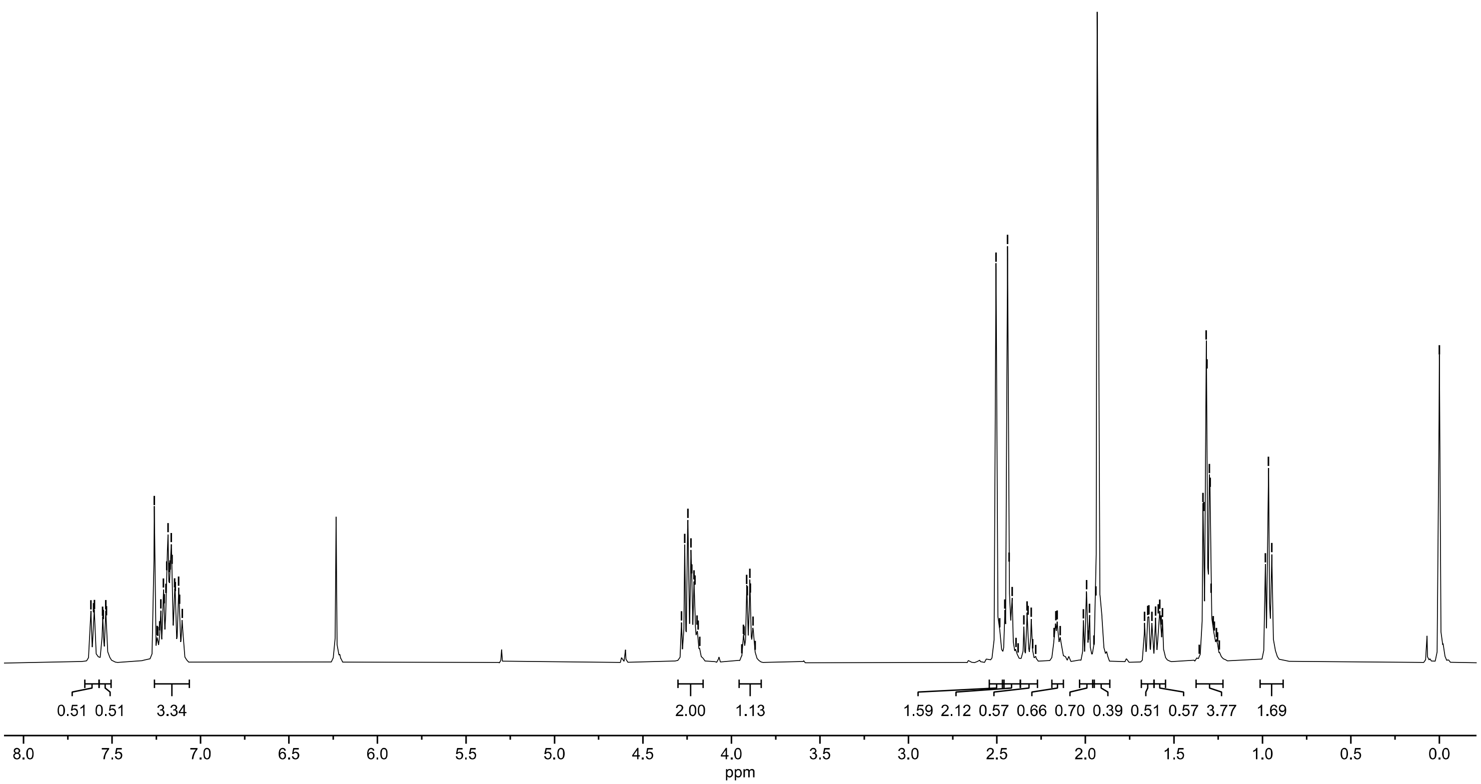

5m. Ethyl (1R,2S)-2-acetoxy-2-(p-tolyl)cyclopropane-1-carboxylate
^{1}H NMR (500 MHz, $CDCl_3$)

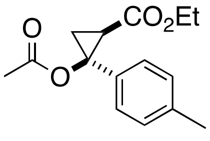

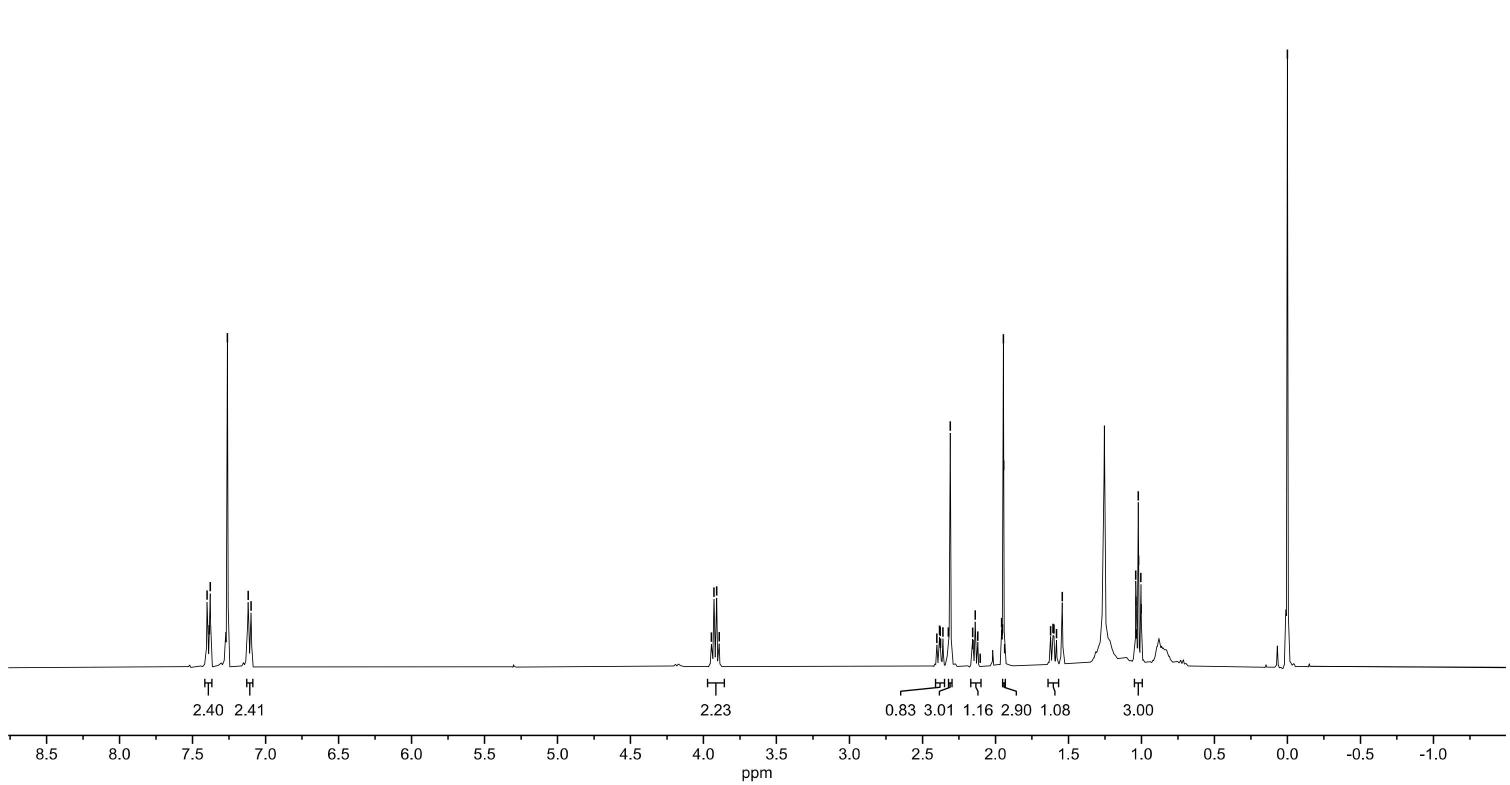

5m. Ethyl (1R,2S)-2-acetoxy-2-(p-tolyl)cyclopropane-1-carboxylate
$^{13}C\{^1H\}$ NMR (126 MHz, $CDCl_3$)

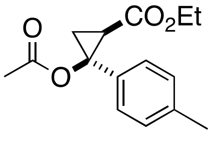

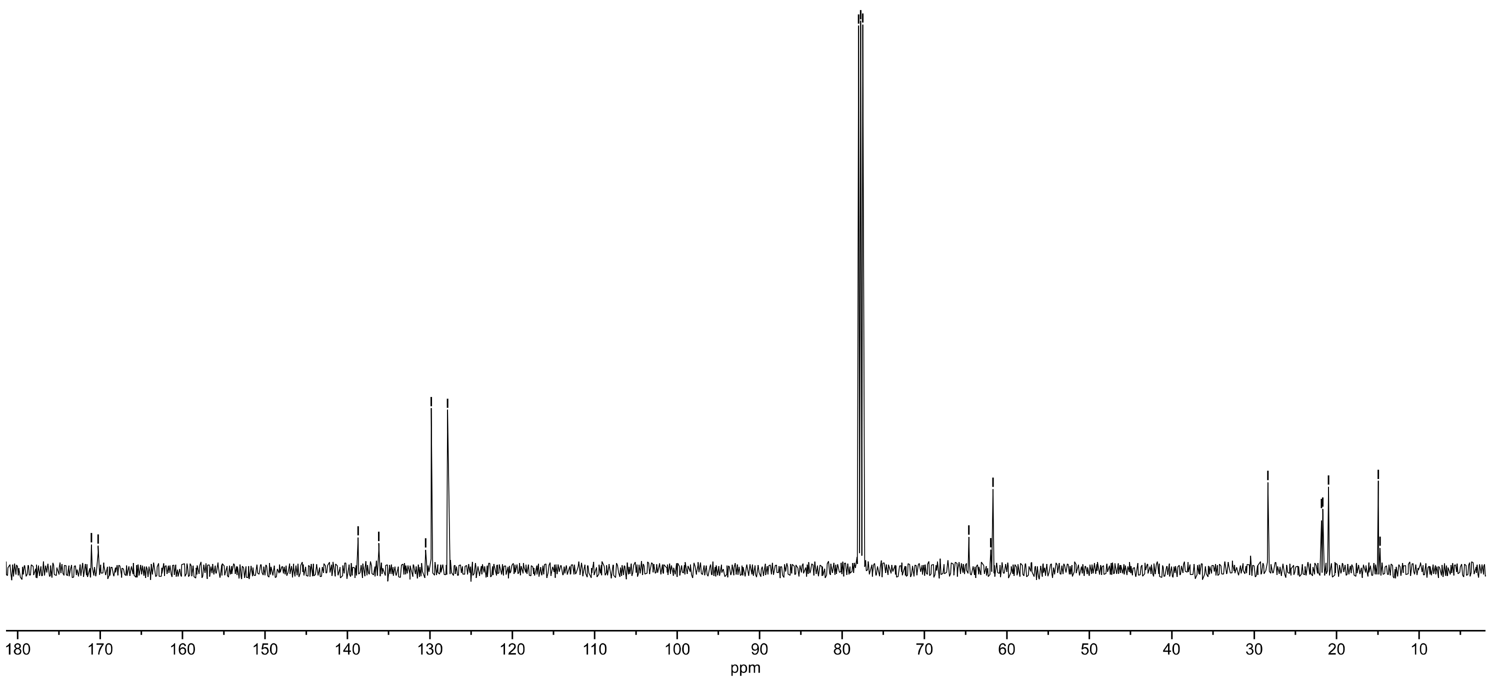

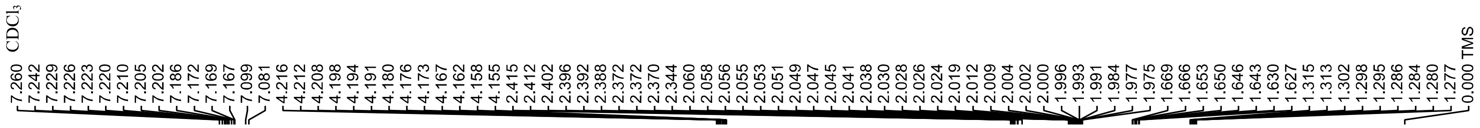

5l. Ethyl (1R,2S)-2-acetoxy-2-(m-tolyl)cyclopropane-1-carboxylate

^{1}H NMR (500 MHz, $CDCl_3$)

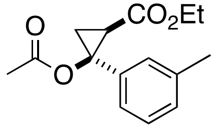

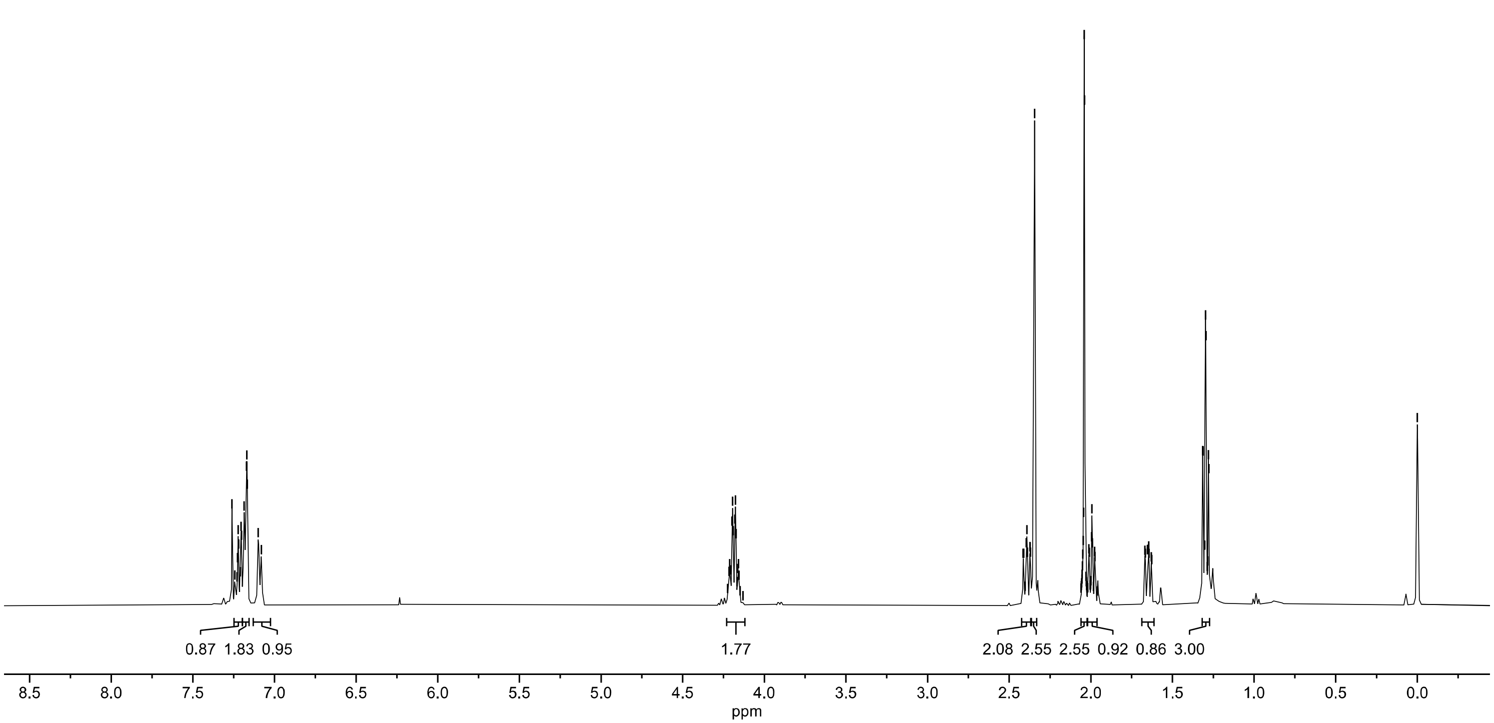

5l. Ethyl (1R,2S)-2-acetoxy-2-(m-tolyl)cyclopropane-1-carboxylate
$^{13}C\{1H\}$ NMR (126 MHz, $CDCl_3$)

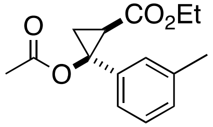

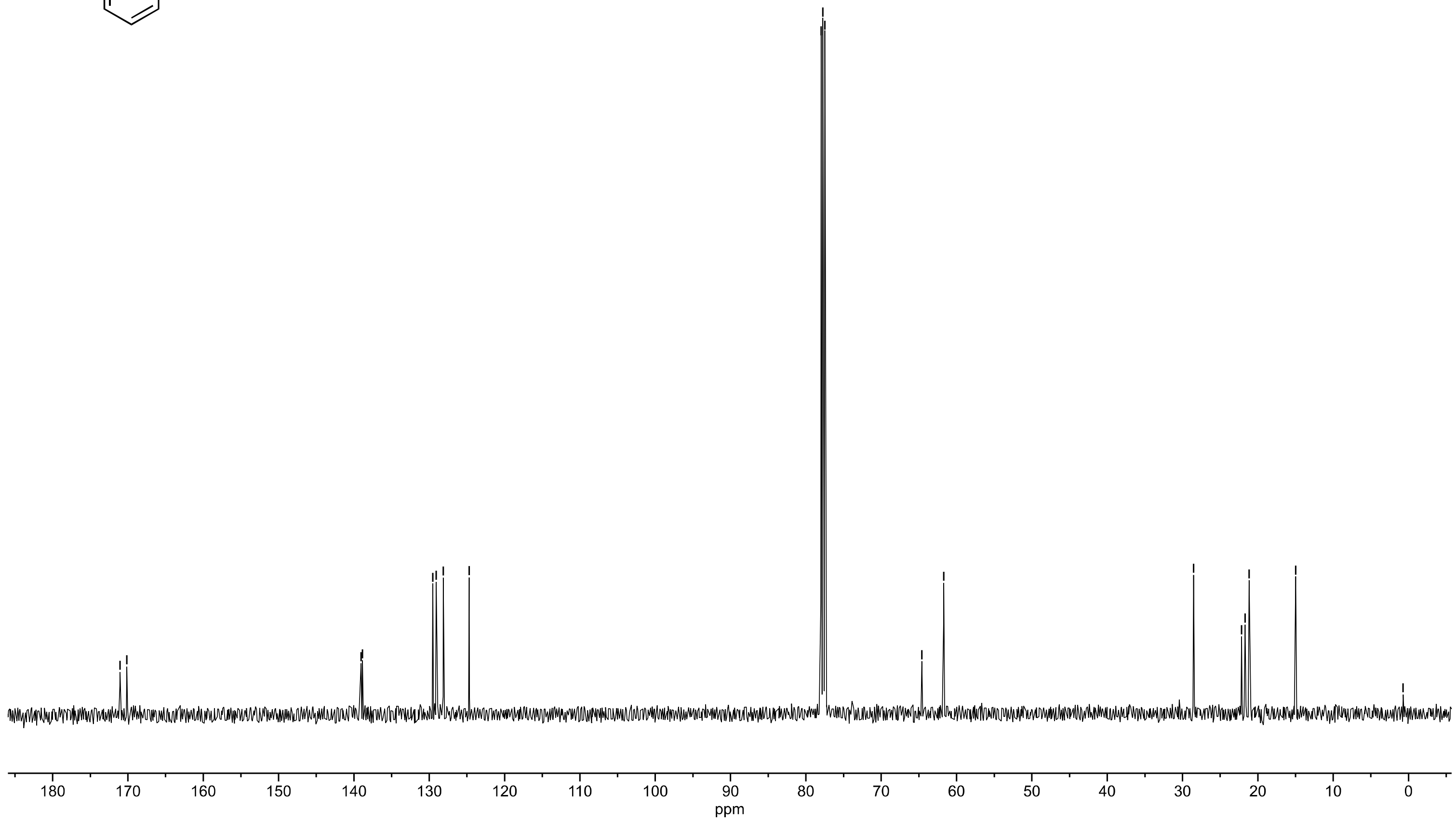

5q. Ethyl (1R,2S)-2-acetoxy-2-(4 methoxyphenyl)cyclopropane-1-carboxylat**e**
^{1}H NMR (500 MHz, $CDCl_3$)

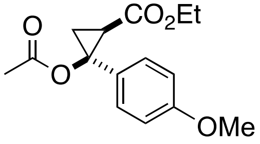

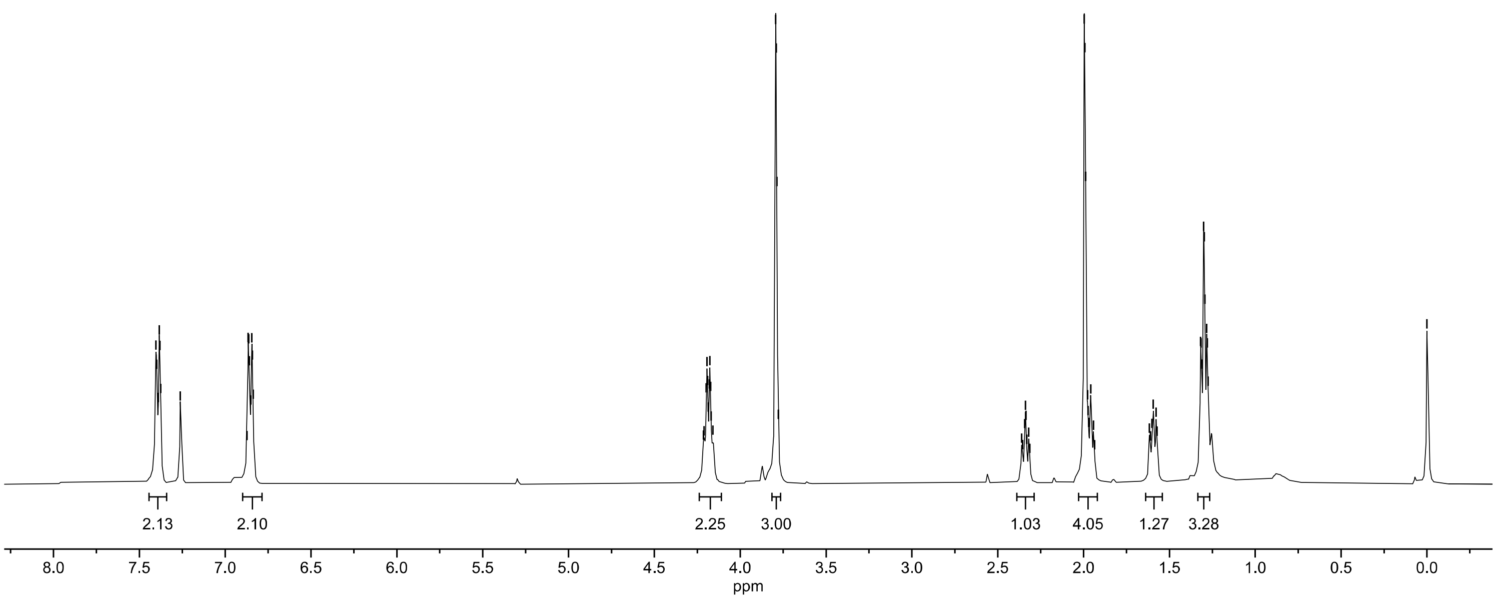

5q. Ethyl (1R,2S)-2-acetoxy-2-(4 methoxyphenyl)cyclopropane-1-carboxylat**e**
$^{13}C\{1H\}$ NMR (126 MHz, $CDCl_3$)

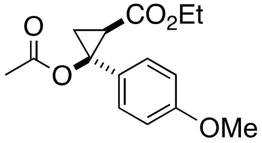

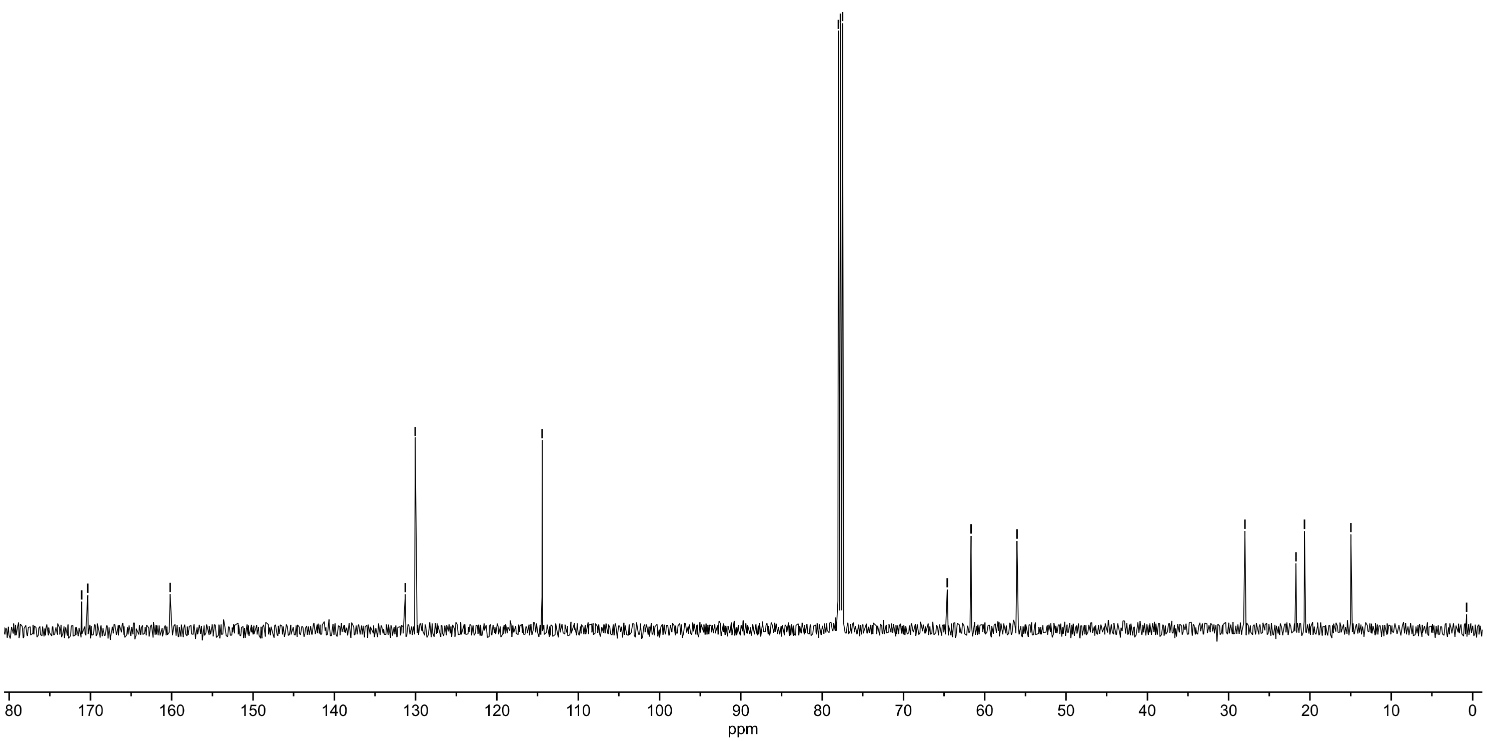

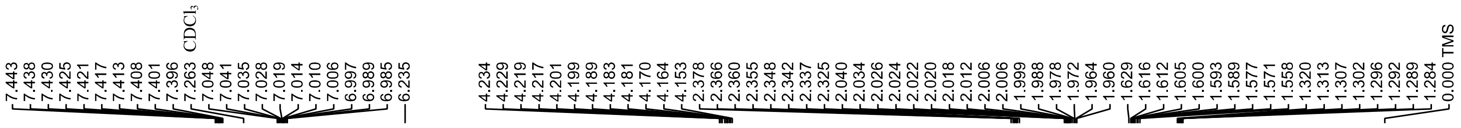

5n. Ethyl (1R,2S)-2-acetoxy-2-(4-fluorophenyl)cyclopropane-1-carboxylate
^{1}H NMR (500 MHz, $CDCl_3$)

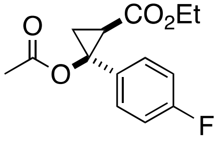

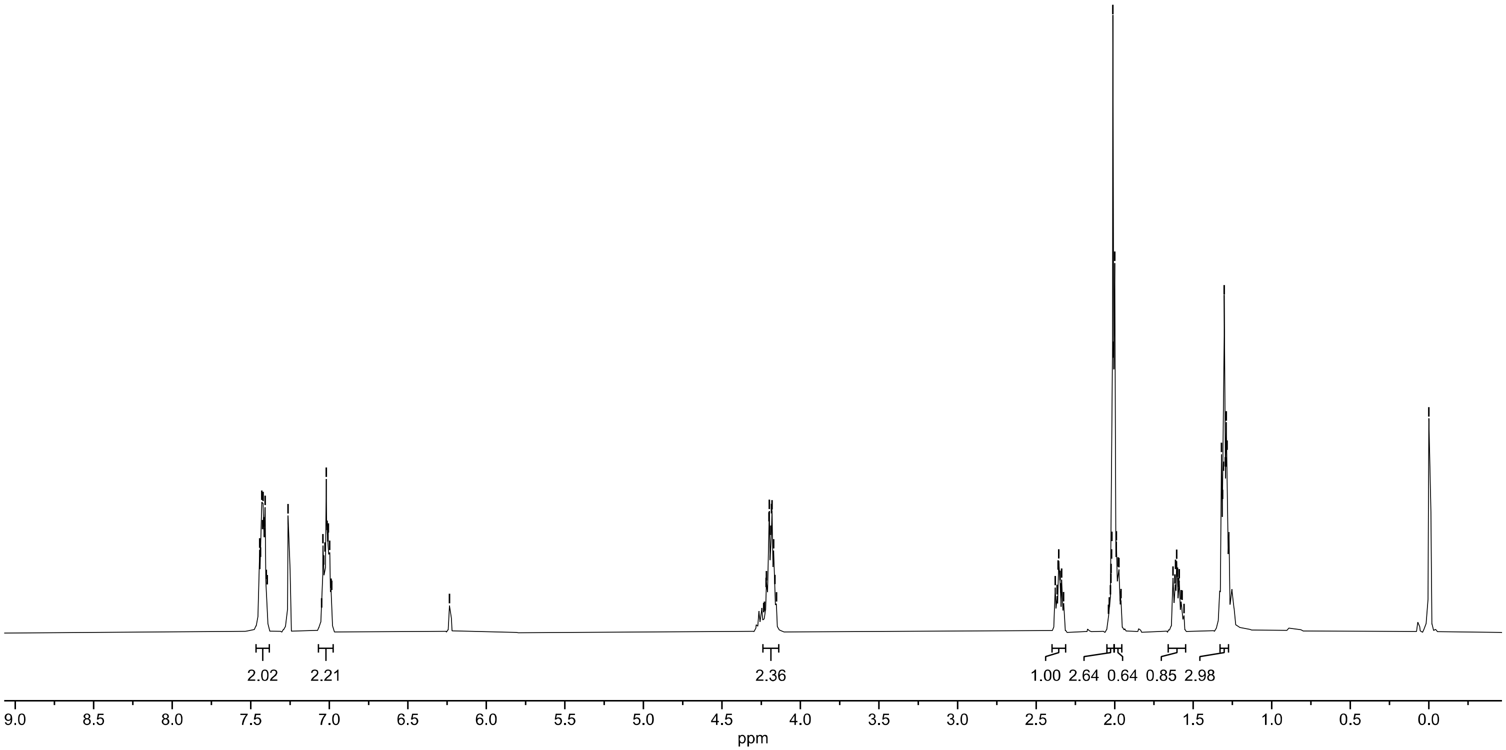

5n. Ethyl (1R,2S)-2-acetoxy-2-(4-fluorophenyl)cyclopropane-1-carboxylate
$^{13}C\{1H\}$ NMR (126 MHz, $CDCl_3$)

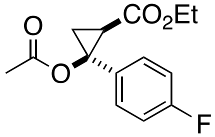

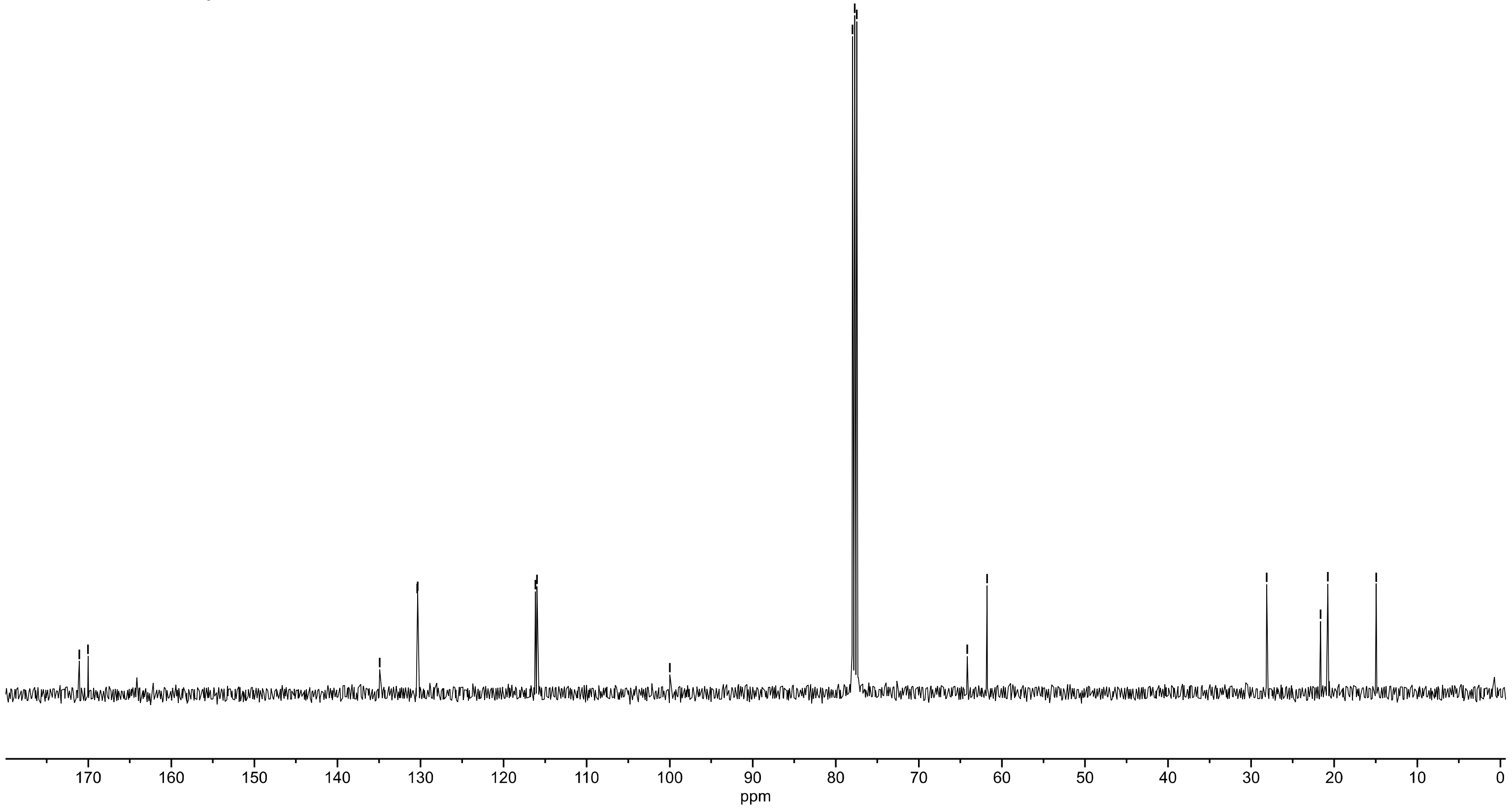

5n. Ethyl (1R,2S)-2-acetoxy-2-(4-fluorophenyl)cyclopropane-1-carboxylate
^{19}F NMR 376 MHz

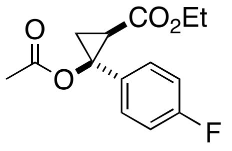

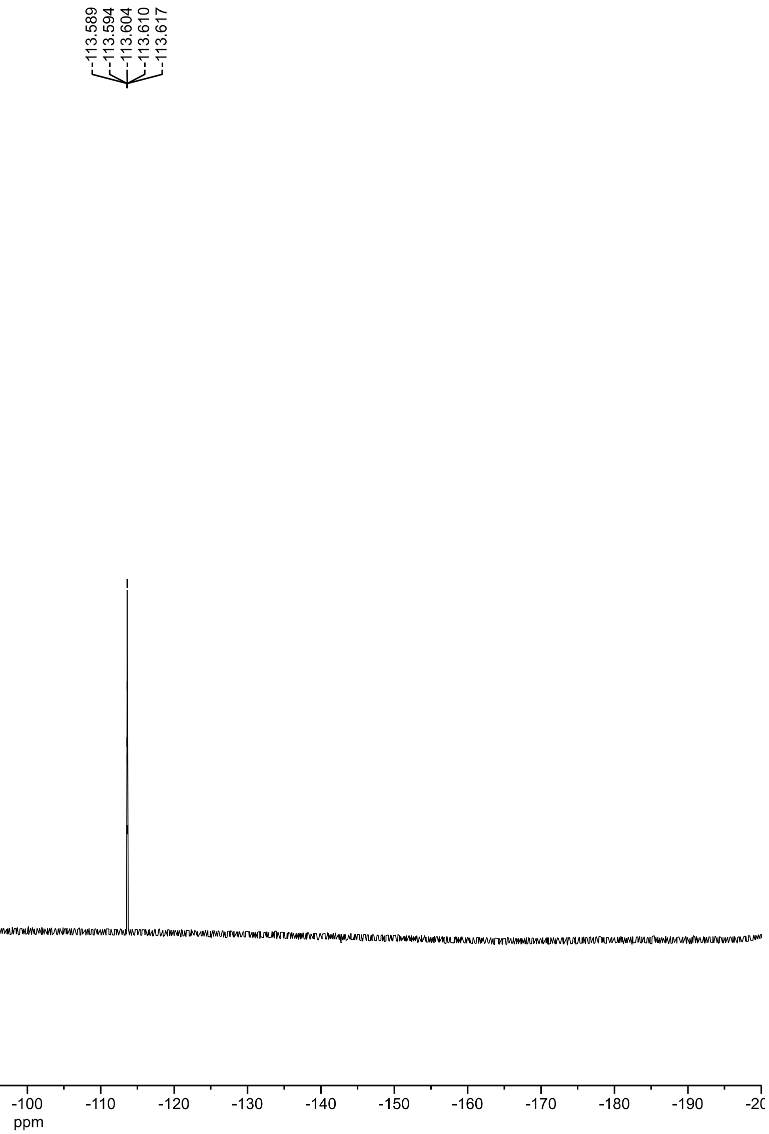

5o. Ethyl (1R,2S)-2-acetoxy-2-(4-bromophenyl)cyclopropane-1-carboxylate
^{1}H NMR (500 MHz, $CDCl_3$)

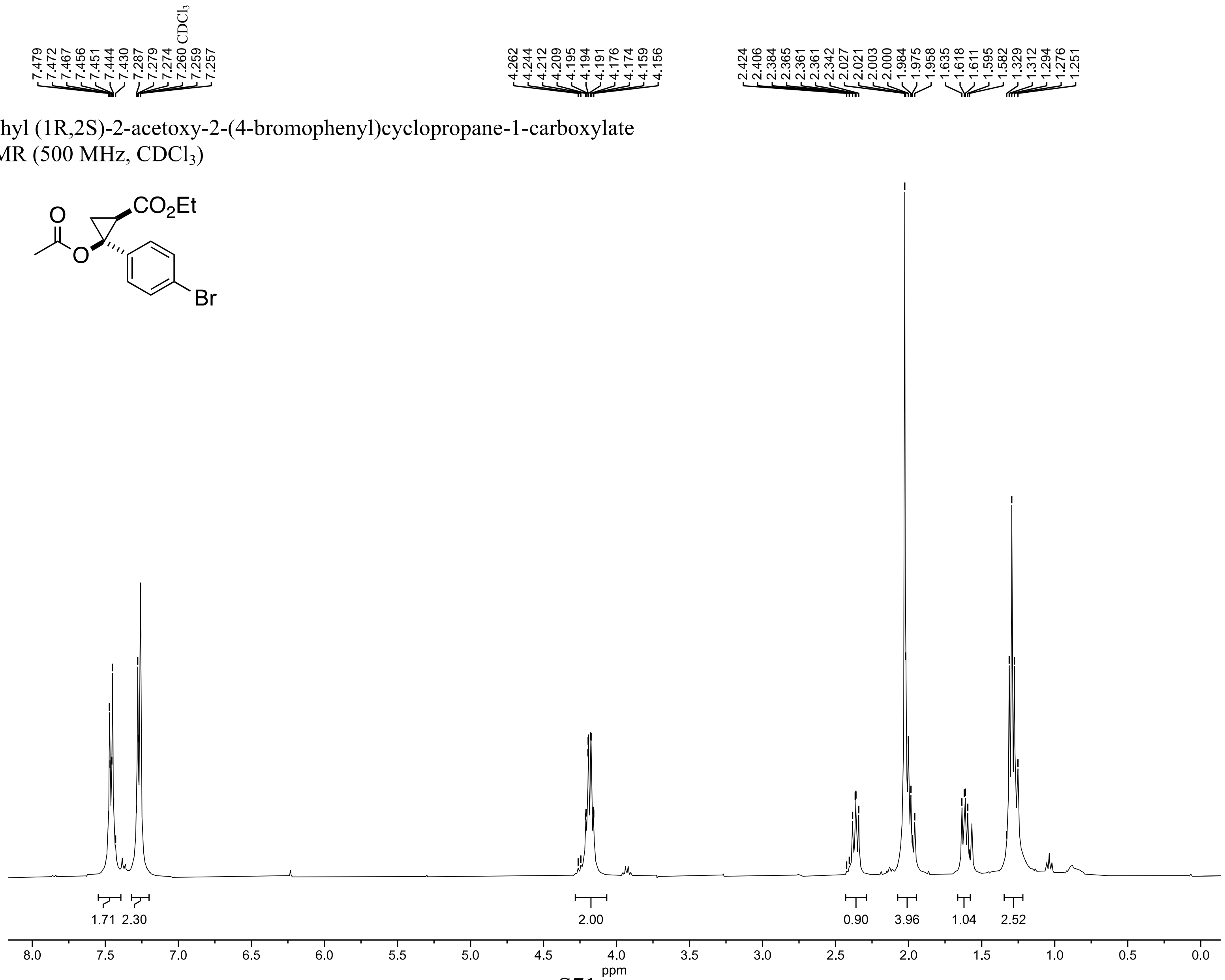

5o. Ethyl (1R,2S)-2-acetoxy-2-(4-bromophenyl)cyclopropane-1-carboxylate
$^{13}C\{^1H\}$ NMR (126 MHz, $CDCl_3$)

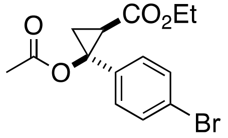

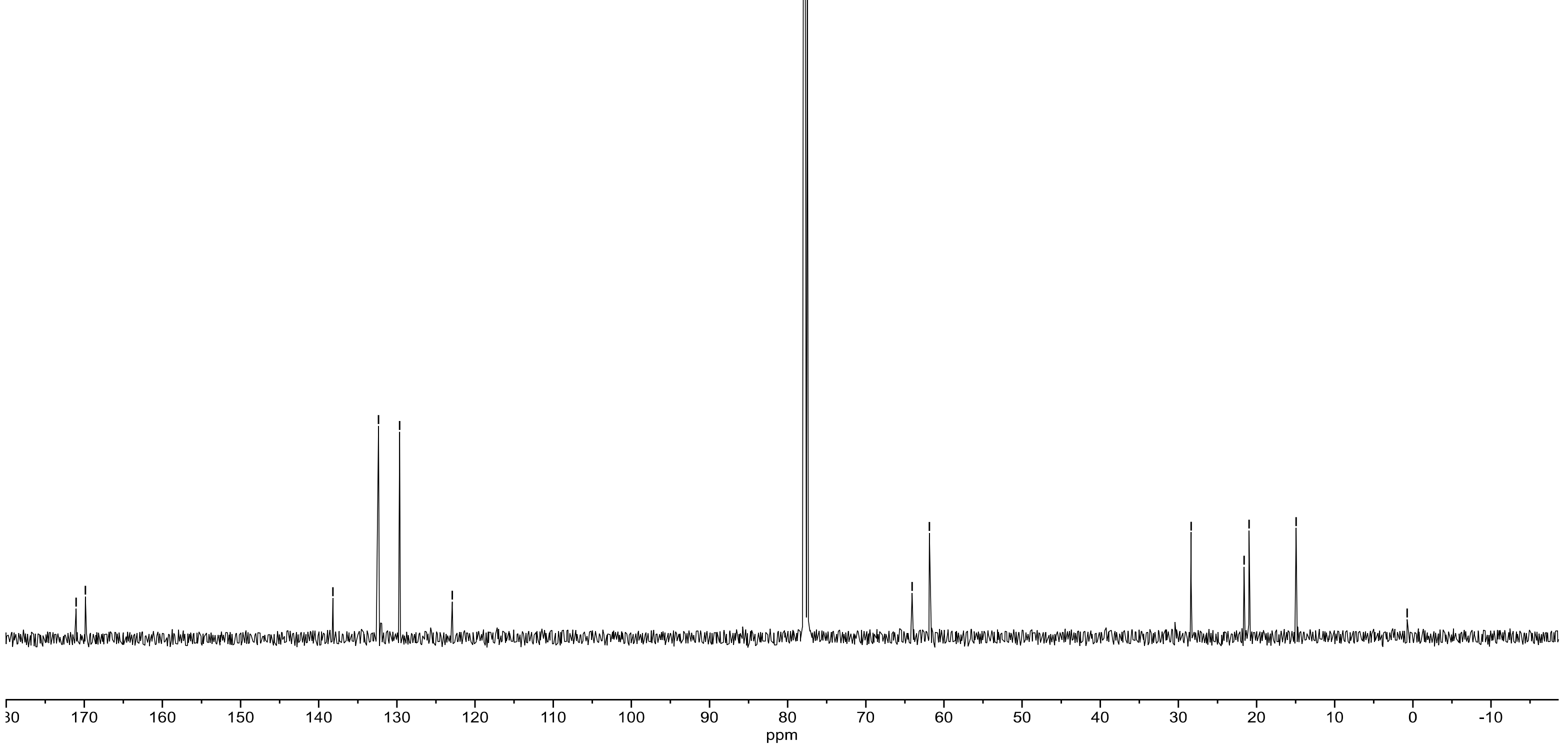

5p. Ethyl (1R,2S)-2-acetoxy-2-(4-(trifluoromethyl)phenyl)cyclopropane-1-carboxylate
^1H NMR (500 MHz, $CDCl_3$)

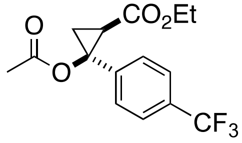

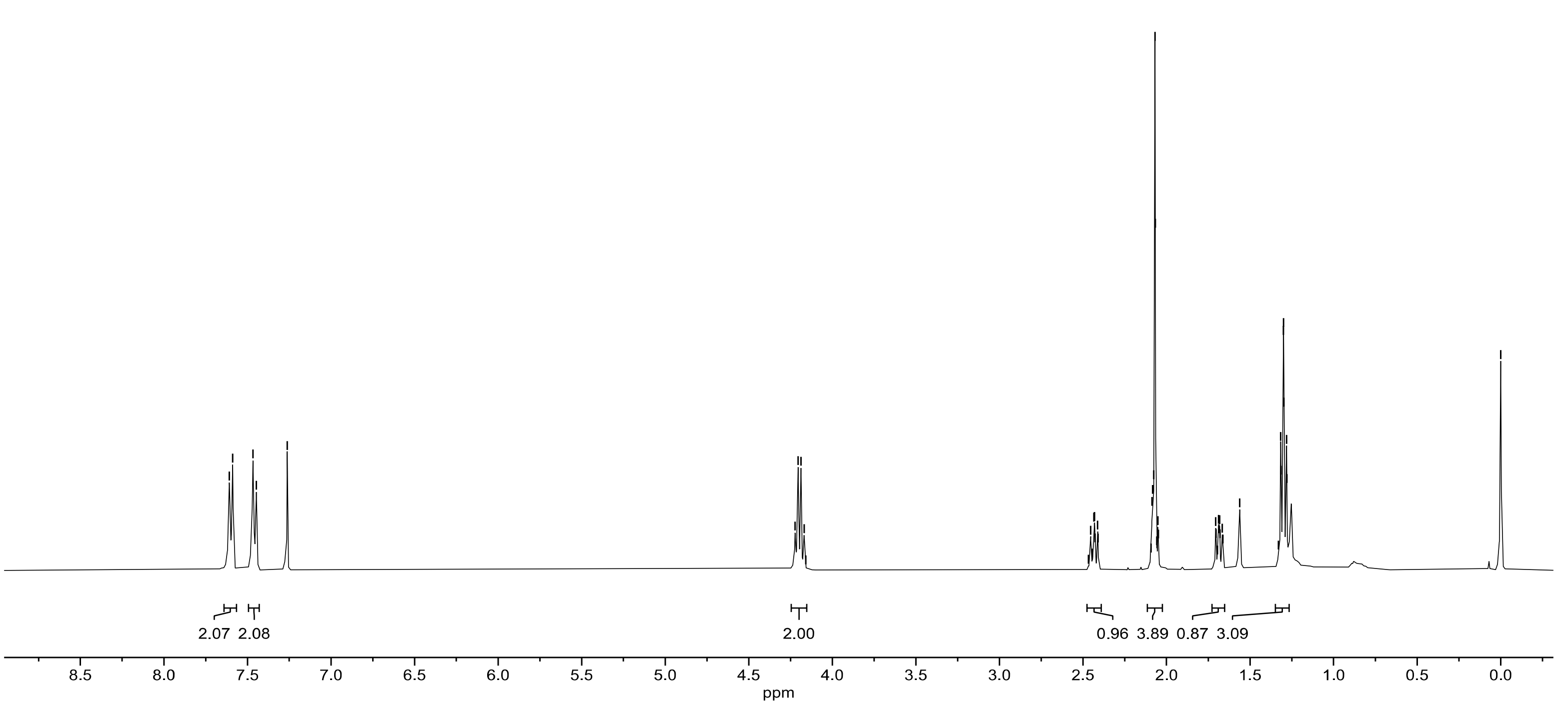

5p. Ethyl (1R,2S)-2-acetoxy-2-(4-(trifluoromethyl)phenyl)cyclopropane-1-carboxylate

$^{13}C\{^1H\}$ NMR (126 MHz, $CDCl_3$)

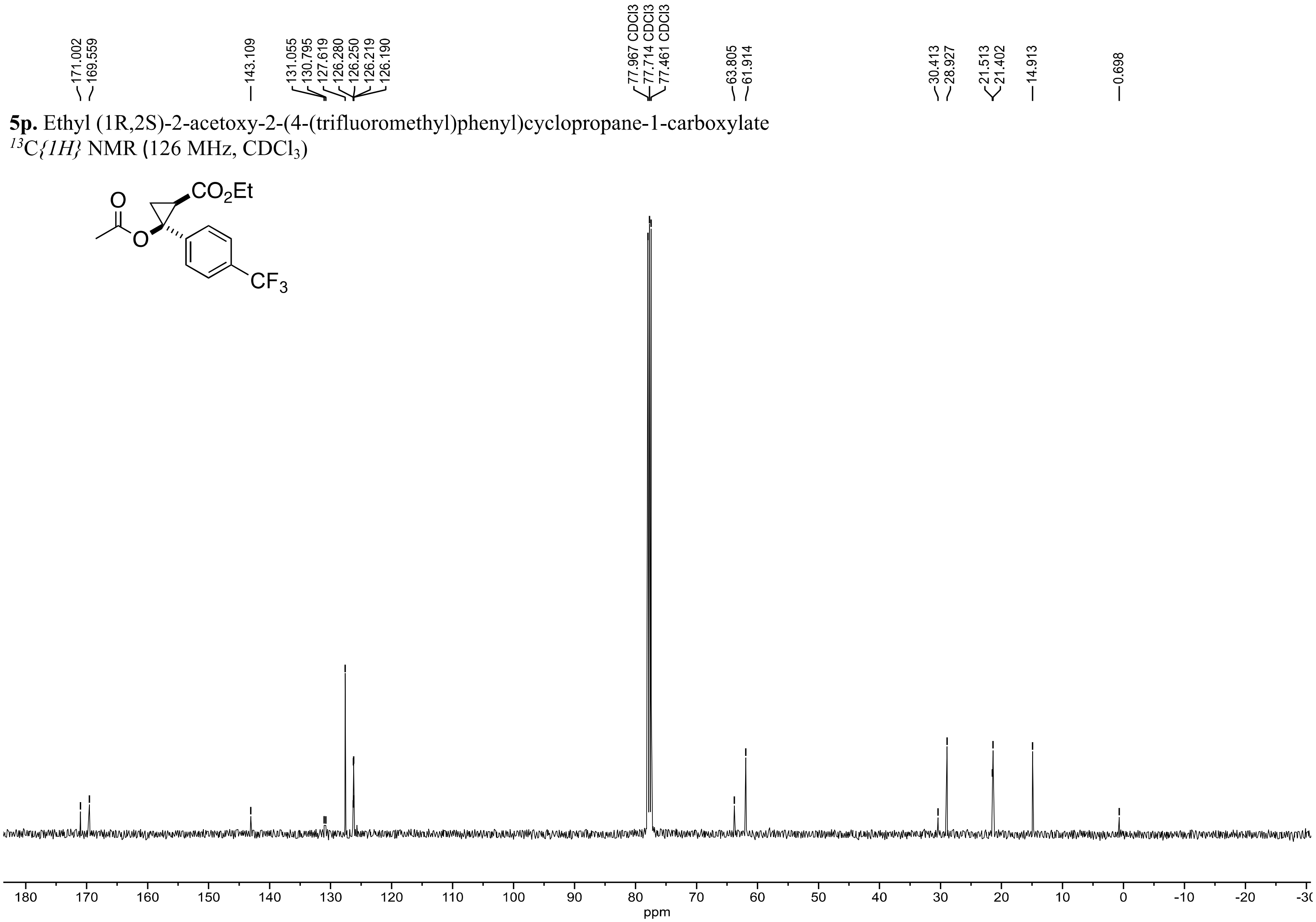

5p. Ethyl (1R,2S)-2-acetoxy-2-(4-(trifluoromethyl)phenyl)cyclopropane-1-carboxylate
^{19}F NMR 376 MHz

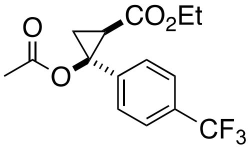

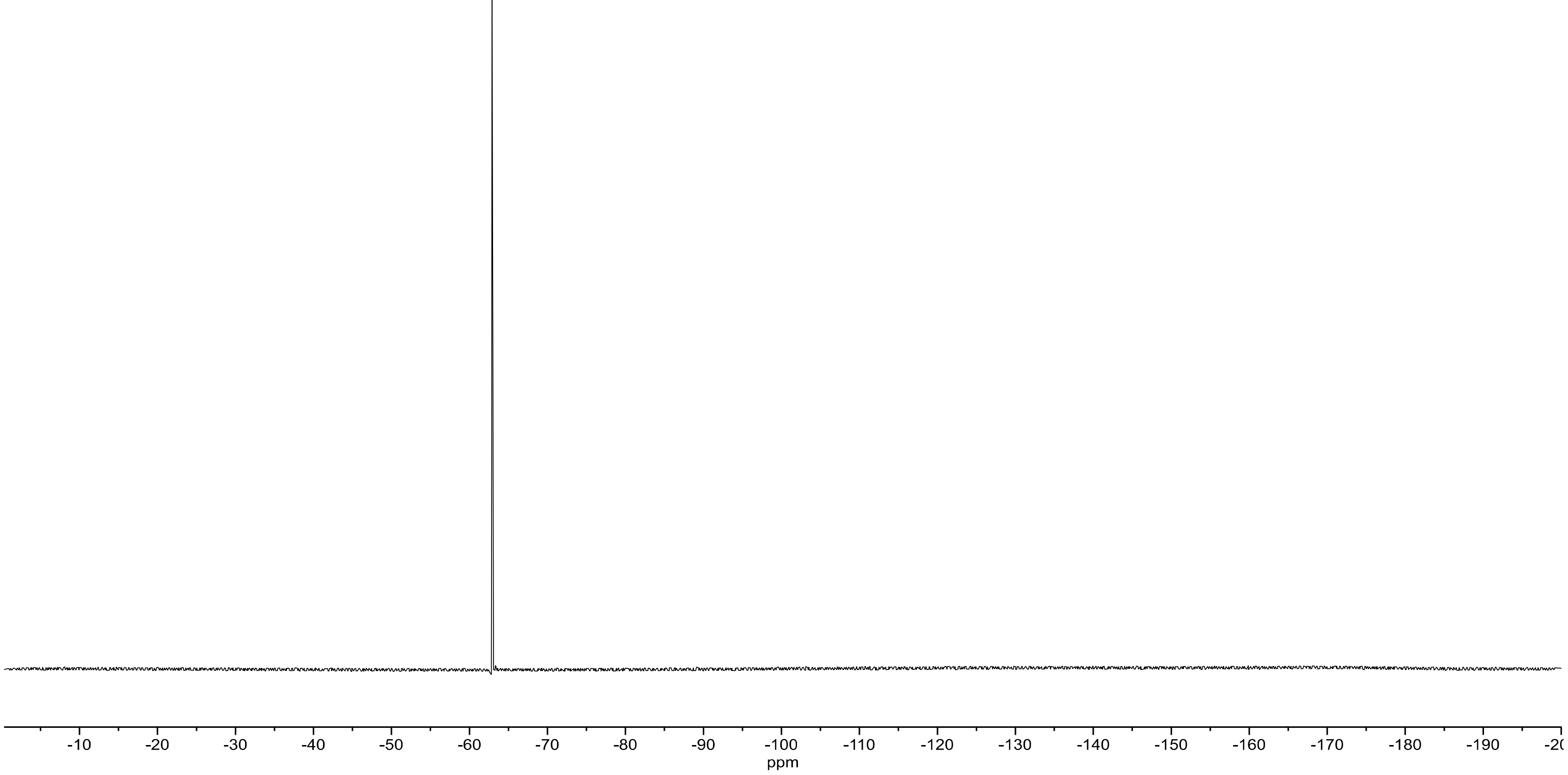

7a. (1S,2R)-2-cyanocyclopropyl benzoate
^{1}H NMR (500 MHz, $CDCl_3$)

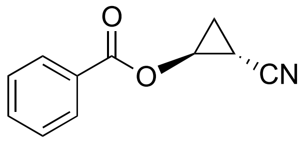

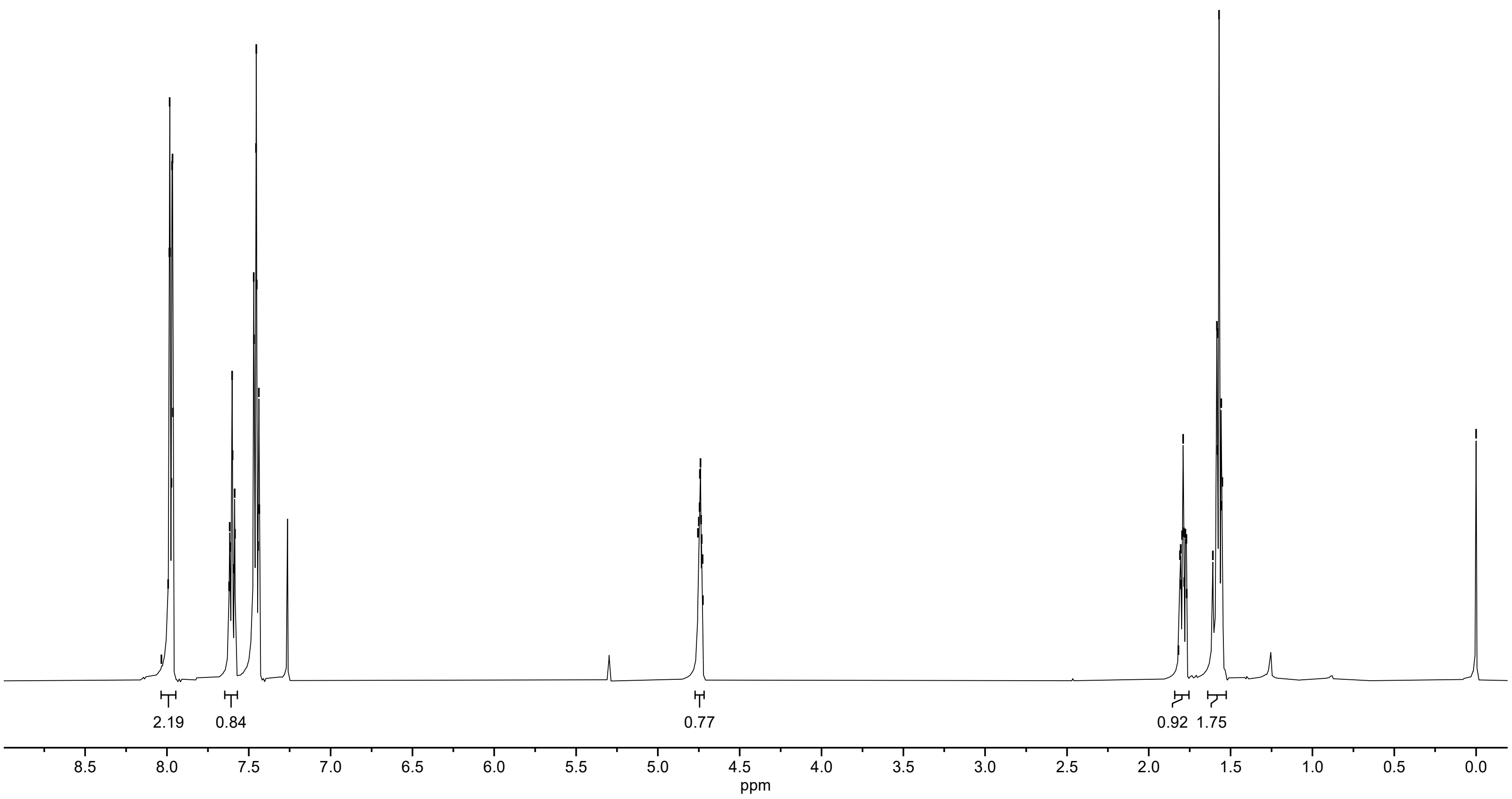

7a. (1S,2R)-2-cyanocyclopropyl benzoate
$^{13}C\{^{1}H\}$ NMR (126 MHz, $CDCl_3$)

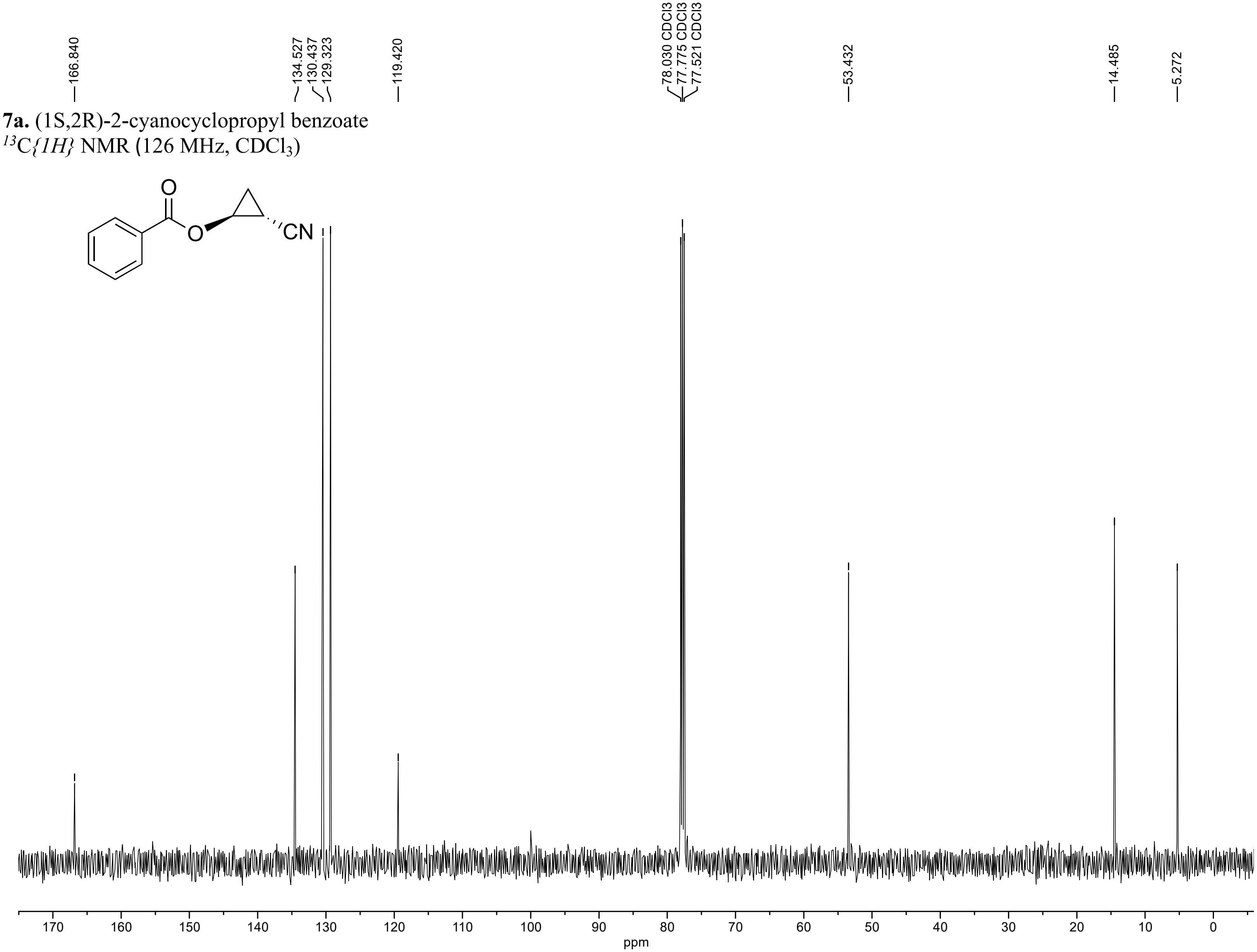

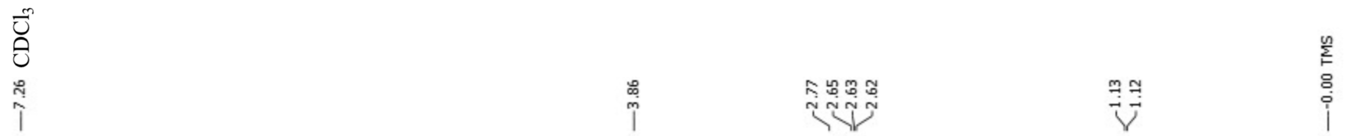

11. (1R,2R)-2-hydroxy-2-isopropylcyclopropane-1-carboxylic acid
^{1}H NMR (500 MHz, $CDCl_3$)

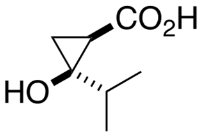

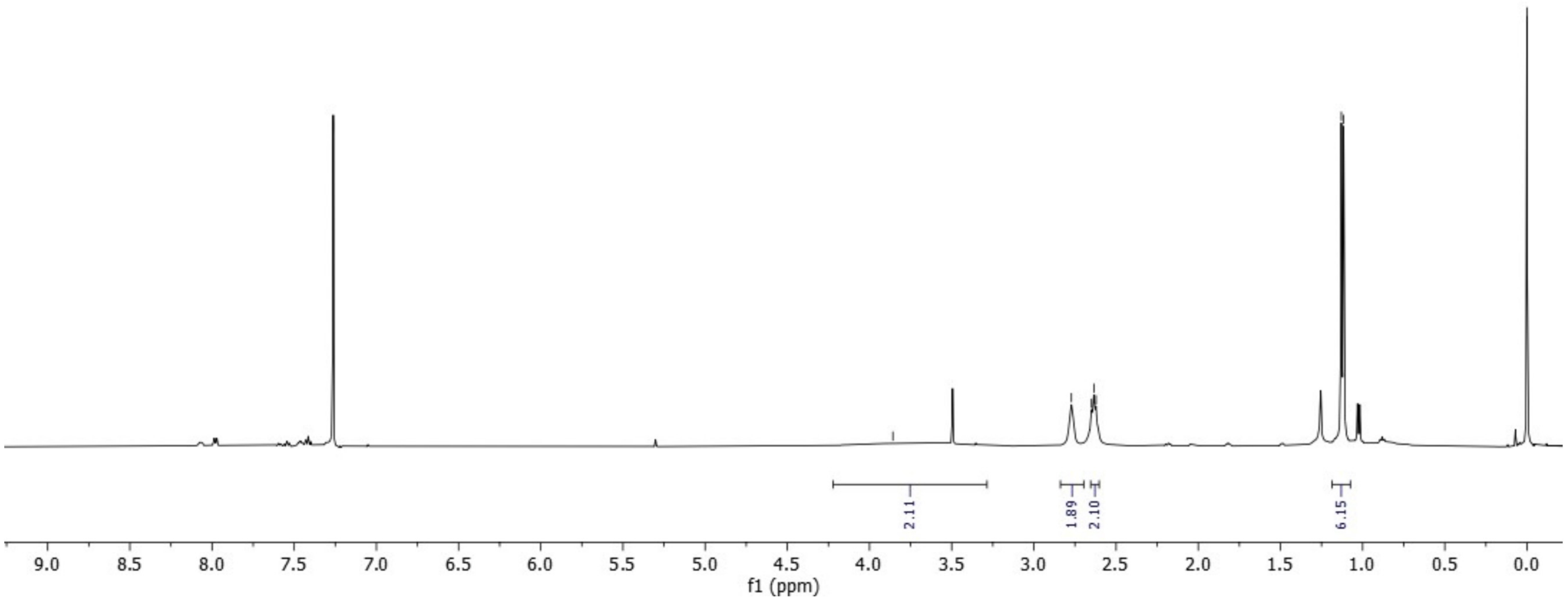

11. (1R,2R)-2-hydroxy-2-isopropylcyclopropane-1-carboxylic acid
$^{13}C\{^{1}H\}$ NMR (126 MHz, $CDCl_3$)

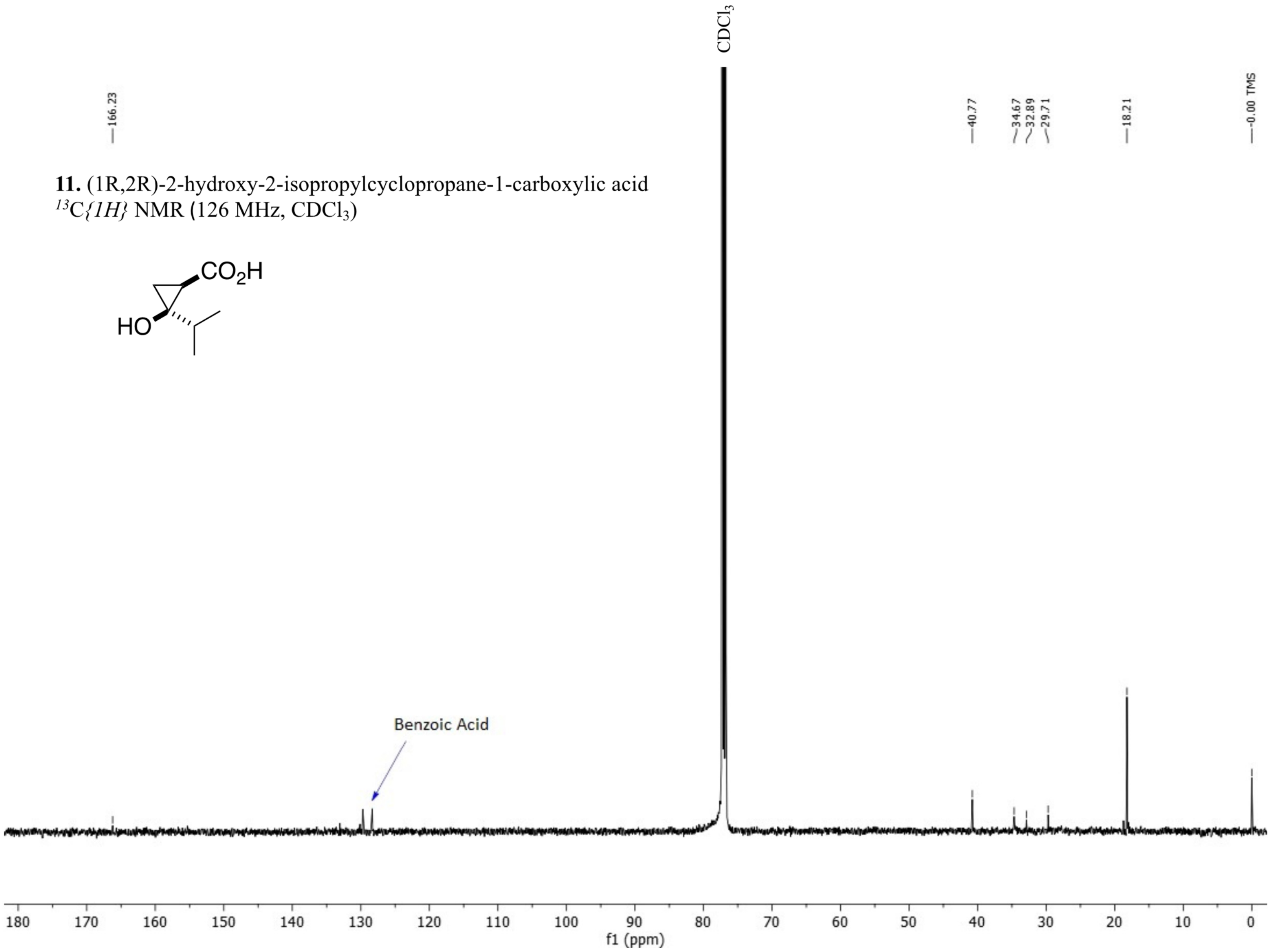

References

1. Martinez-Montero, S.; Fernandez, S.; Sanghvi, Y. S.; Gotor, V.; Ferrero, M. An Expedient Biocatalytic Procedure for Abasic Site Precursors Useful in Oligonucleotide Synthesis. *Org. Biomol. Chem.* **2011**, *9*, 5960-5966

2. Goossen, L. J.; Paetzold, J.; Koley, D. Regiocontrolled Ru-Catalyzed Addition of Carboxylic Acids to Alkynes: Practical Protocols for the Synthesis of Vinyl Esters. *Chem. Commun.* **2003**, 706-707.

3. Li, W. P.; Zhu, Y. C.; Zhou, Y. J.; Yang, H. W.; Zhu, C. J. Visible Light Induced C-H Monofluoroalkylation to Synthesize 1,4-Unsaturated Compound. *Tetrahedron* **2019**, *75*, 1647-1651.

4. Sreenilayam, G.; Fasan, R. Myoglobin-Catalyzed Intermolecular Carbene N-H Insertion with Arylamine Substrates. *Chem. Commun.* **2015**, *51*, 1532-1534.

5. Tyagi, V.; Bonn, R. B.; Fasan, R. Intermolecular Carbene S-H Insertion Catalysed by Engineered Myoglobin-Based Catalysts. *Chem. Sci.* **2015**, *6*, 2488-2494.

6. Tyagi, V.; Sreenilayam, G.; Bajaj, P.; Tinoco, A.; Fasan, R. Biocatalytic Synthesis of Allylic and Allenyl Sulfides through a Myoglobin-Catalyzed Doyle-Kirmse Reaction. *Angew. Chem. Int. Ed.* **2016**, *55*, 13562-13566.
